# Microbially Derived P=S and P=Se Bond Formation

**DOI:** 10.1021/jacsau.5c00262

**Published:** 2025-03-29

**Authors:** Connor L. Trotter, Yuta Era, Rory Gordon, Samantha Law, Christopher H. Switzer, Stephen Wallace

**Affiliations:** 1 Institute of Quantitative Biology, Biochemistry and Biotechnology, School of Biological Sciences, University of Edinburgh, Edinburgh EH9 3FF, U.K.; 2 EaStCHEM School of Chemistry, University of Edinburgh, Edinburgh EH9 3FJ, U.K.; 3 NCIMB Ltd., Wellheads Place, Dyce, Aberdeen AB21 7GB, U.K.; 4 Department of Molecular and Cell Biology, University of Leicester, Leicester LE1 7RH, U.K.

**Keywords:** microbial chemistry, biotransformation, cellular sulfanes

## Abstract

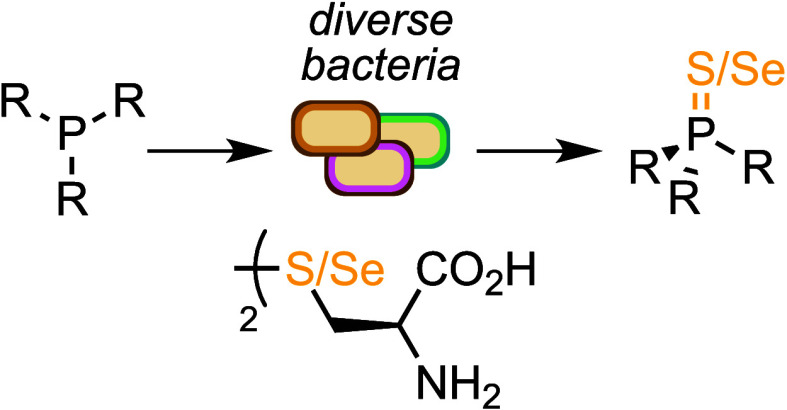

Microbial metabolism is a diverse and sustainable source of synthetic reagents that can be programmed for controlled and high-level production via synthetic biology. However, despite the chemical diversity of metabolism, the chemical utility of metabolites, and the available tools to control metabolic chemistry, there remain few examples of the use of cellular metabolites directly for chemical synthesis. Herein, we report that diverse bacteria perform P=S bond formation (Ph_3_P to Ph_3_PS) via central sulfur metabolism and nonenzymatic chemistry *in vivo*, which can also be applied to affect microbial P=Se bond formation (Ph_3_PSe). To the best of our knowledge, this is the first biochemical and genetic investigation of P=S bond formation in a microbial cell and the first use of microbial metabolites for P=Se bond formation in chemical synthesis.

## Introduction

In Nature, main group sp^2^-sp^2^ bonds are limited to those formed during photosynthesis (O_2_), biosynthesis (C = N, P = O, S = O and N = N), or broken during assimilatory metabolism (N_2_ and SO_2_) and along respiratory electron transport chains (O_2_, S = O and NOR). However, the formation of similar sp^2^-sp^2^ bonds between phosphorus and sulfur (P = S) is rarely observed in biology.^1^ In chemistry, P = S bonds are found in synthetic reagents used for episulfide or thioketene formation^2^ and within phosphorothioate backbones of nucleotide drug candidates to protect against metabolic degradation and enable cell penetration *in vivo*.^3,4^ The lack of established methods to form P = S using biological tools means the functional transformation remains only accessible by chemical synthesis. Existing methods for this largely rely on the rection of P(III) substrates with elemental or electrophilic sulfur reagents in organic solvent under abiotic conditions.^5^ This is despite the wealth of electrophilic S^δ+^ metabolites that exist in biological systems which can be intercepted using biocompatible chemistry or overproduced for delivery into existing reactions using synthetic biology. Herein we report the discovery that diverse microorganisms can be used to perform P = S and P = Se bond formation by interfacing nonenzymatic R_3_P oxidation with native sulfur metabolic pathways *in vivo* and apply this to the synthesis of triphenylphosphine sulfide and triphenylphosphine selenide.

## Results and Discussion

**Figure 1:**
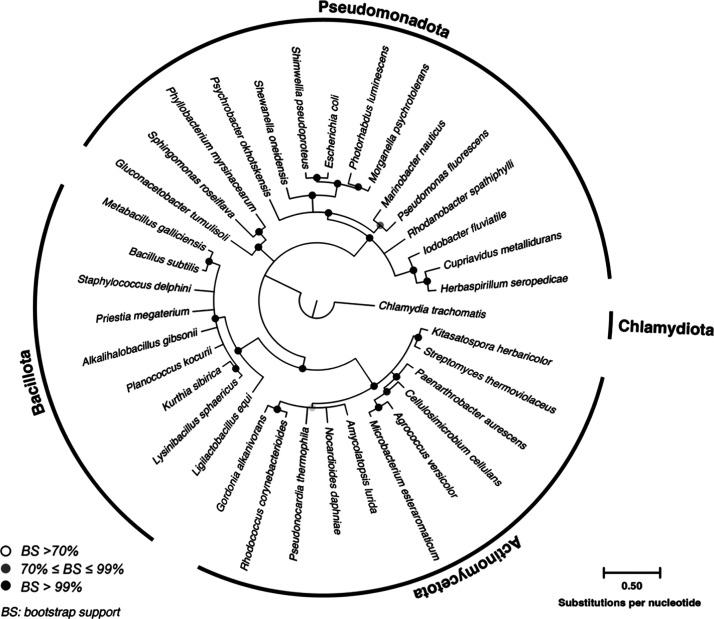
Maximum Likelihood tree showing the phylogenetic diversity of microorganisms used for screening. Tree was constructed using the General Time Reversible (GTR) substitution model with gamma distributed rates (+G) and invariable sites (+I) and shows consensus of nodes with >70% bootstrap support based upon 500 pseudoreplicates. *Chlamydia trachomatis* was used as an outgroup to infer genetic distance. Bar represents 0.5 substitutions per nucleotide position.

Discovery of these unusual microbial reactions began by screening diverse, chemically uncharacterized bacteria from the National Collection of Industrial, Food and Marine Bacteria (NCIMB) for the ability to modify P-containing small molecules. A panel of 35 species were curated from a range of environments with different culturing conditions and varying degrees of established literature (Figure 1 and Table S1). Using a diverse collection of bacteria in this manner enabled a broader screen of microbial metabolisms than what could be captured by using a limited range of model organisms, thus enabling a shotgun approach to identifying novel biochemistries that may have evolved under unique ecological conditions. Trends arising from these data could then be used to identify suitable model systems for characterization. Small molecule targets were limited to triphenylphosphine (Ph_3_P) and triphenylphosphine oxide (Ph_3_PO). While Ph_3_PO is considered a waste product, modification presents an interesting opportunity to regenerate Ph_3_P for further industrial scale use in various named synthesis reactions^6^ or produce other useful phosphines and phospholes.^7^ For screening, cultures were incubated to accumulate significant biomass (OD_600_ > 0.4), after which phosphines were added at 3 mM under aerobic and microaerobic conditions then incubated for 44 h (220 rpm). Cultures were extracted with organic solvent then analyzed by ^31^P NMR spectroscopy.

**Figure 2:**
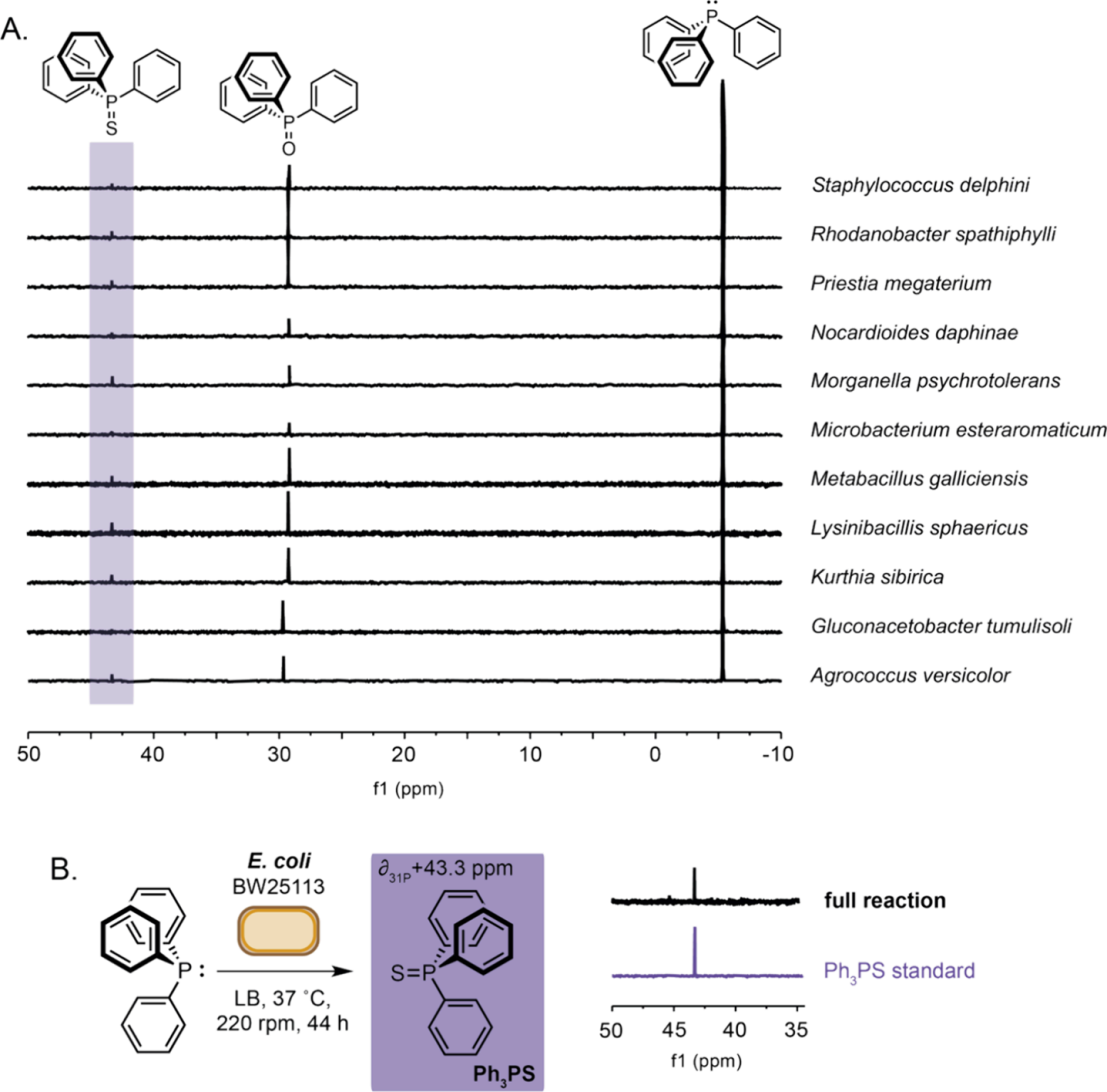
A) ^31^P NMR spectra from extracts of microbial cultures incubated in the presence of triphenylphosphine. B) ^31^P NMR spectra from a culture of *E. coli* BW25113 incubated in the presence of triphenylphosphine. Reaction conditions: Ph_3_P (3 mM) added to culture of *E. coli* BW25113_pET28a(+) (OD_600_ 0.4–0.6) in LB media and incubated at 37 °C (220 rpm) for 44 h before extraction using ethyl acetate and analysis by ^31^P NMR spectroscopy.

Although no NMR signal changes were observed in any Ph_3_PO cultures (Figures S3–S5), a small additional downfield peak at δ + 43.4 ppm was observed in 11 different species in addition to <5% Ph_3_PO autoxidation (Figure 2A and Figures S6–S9). Such a large deshielded shift in ^31^P resonance from δ −5.4 ppm to δ + 43.4 ppm suggested direct modification of the P(III) atom by an electronegative group. Considering established P(V) main group chemistry literature, the peak was identified as triphenylphosphine sulfide (Ph_3_PS).^8^ Intrigued by this modification, we sought to understand it further; however, many screened species lacked detailed metabolic characterization and annotated genomes and presented challenges to identifying the reason(s) why the bacteria could mediate this chemistry.

**Figure 3:**
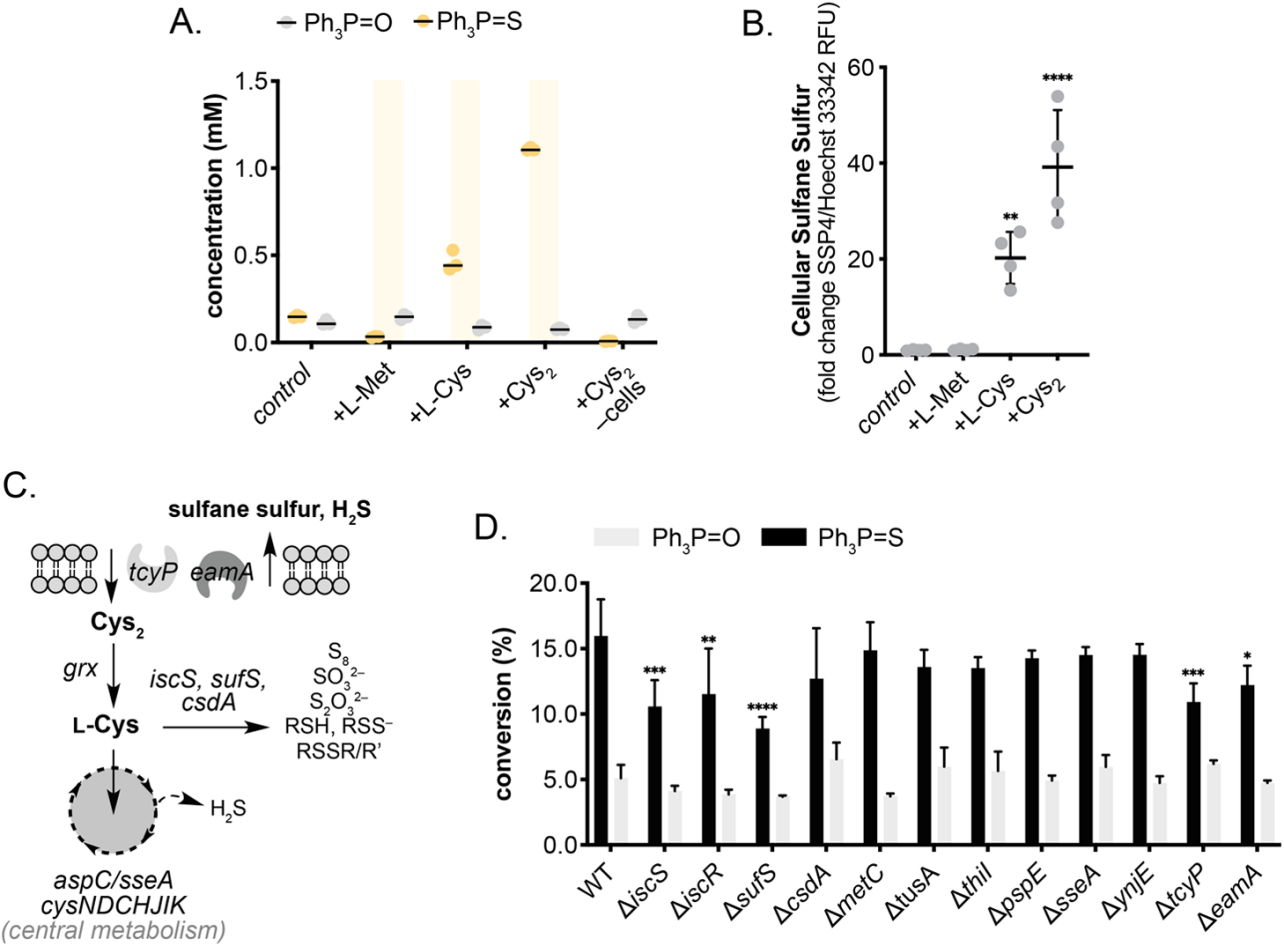
A) Amino acid dependent Ph_3_PS formation by *E. coli*. B) Amino acid dependent sulfane sulfur formation by *E. coli* via SSP4 fluorescence normalized to DNA content (Hoechst 33342). Data represent the mean fold change (±standard error of mean) of four biological replicates, each with two technical replicates. Statistical significance relative to control was determined by one-way ANOVA with Dunnett’s posthoc test. C) Key sulfur metabolism pathways in *Escherichia* sp.. D) Effect of sulfur metabolism gene knockouts on Ph_3_PS formation by *E. coli* BW25113. Reaction conditions: LB media +50 mM l-Cys, OD_600_ 0.4–0.6, 37 °C, 220 rpm, 44 h. Data shown are an average of three replicates to one standard deviation. Symbols indicate statistical significance: *p < 0.05, ** *p* < 0.005, *** *p* < 0.0005, **** *p* < 0.0001.

Phylogenetic analysis showed P = S bond formation was spread throughout the screened phyla – including members of the *Actinomycetota*, *Bacillota*, and *Pseudomonadota*. Given the clustering of P = S formation within closely related clades, such as *Lysinibacillus sphaericus* and *Kurthia sibirica* or *Agrococcus versicolor* and *Microbacterium esteraromaticum*, P = S formation by *Morganella psychrotolerans* suggested this transformation may be completed by *Escherichia coli.* Indeed, we found *E. coli* BW25113 could facilitate Ph_3_PS formation under identical reaction conditions at a variety of growth stages (Figure 2B and Figures S10–S11). *E. coli* is an extremely well characterized Gram-negative bacterium and so further characterization of P = S bond forming chemistry using this model system was deemed appropriate. As such, we first moved to identify the metabolic sulfur source enabling the reaction. Bacterial sulfur metabolism is diverse, multiplexed, and highly regulated but can ultimately be simplified to the biochemical reactions centring around the interplay of cysteine metabolism (Figure S12). Based on this understanding, *E. coli* cultures were supplemented with either 50 mM l-Cys or l-Met or 25 mM Nb. Cys_2_ as a primary sulfur source. While l-Met had no effect on Ph_3_PS conversion, l-Cys and Nb. Cys_2_ resulted in a 3.1- and 7.4-fold increase in Ph_3_PS formation respectively (Figure 3A and Figures S13–S14). Live cells were required for product formation as the addition of Nb. Cys_2_ to LB media in the absence of cells did not increase conversion to Ph_3_PS.

After confirming cellular involvement and identifying suitable primary sulfur sources that improved P = S bond formation, we sought to further identify suitable reactive sulfur species that form during amino acid supplementation. Within cells, electrophilic sulfur is typically restricted to sulfane sulfur (RSS_n_H) species involved in cofactor biosynthesis and cell-to-cell communication. Moreover, increased sulfane sulfur production in *E. coli* has previously been linked to l-Cys supplementation.^9^ By quantifying the relative fluorescence of a sulfane sulfur probe^10^ normalized to a DNA counterstain, we confirmed increased sulfane sulfur production in l-Cys and Nb. Cys_2_ supplemented cultures and no increase in l-Met supplemented cultures (Figure 3B). Observed increases in sulfane sulfur align with trends observed in Ph_3_PS formation under identical conditions, implicating the role of amino acid-derived sulfane sulfur electrophiles in P = S bond formation *in vivo*. This was confirmed by reacting Ph_3_P with K_2_S_n_ and S_8_ as model sulfane sulfur compounds *in vitro*, achieving >95% conversion under reaction conditions (Figure S15).

Despite confirming abiotic reactivity between microbial sulfane sulfurs and Ph_3_P, further characterization of the *E. coli* system was required to pinpoint the biotic origin of these reactive sulfur compounds. To further investigate potential enzymatic sulfane sulfur sources, we screened 12 KEIO *E. coli* knockout strains in the presence of 3 mM Ph_3_P and 50 mM l-Cys. These knockouts examined key points in enzymatic persulfide (enzyme-SSH) biochemistry either directly, by targeting persulfide-generating desulfurases (Δ*iscS*, Δ*sufS*, Δ*csdA*) and sulfur transferases (Δ*thiI*, Δ*tusA,* Δ*sseA,* Δ*ynjE,* and Δ*pspE*), or indirectly by targeting regulators (Δ*iscR*), transport proteins (Δ*tcyP* and Δ*eamA*) and alternative Nb. Cys_2_ utilizing pathways (Δ*metC;*Figure 3C). By restricting persulfide generation, we anticipated knockouts would reduce Ph_3_PS conversion by reducing sulfane sulfur biosynthesis.

Indeed, knockouts of the major cysteine desulfurases, Δ*iscS* and Δ*sufS*, resulted in a significant reduction in isolated Ph_3_PS yield from 16% to 11% and 9% respectively (Figure 3D and Figure S16). Unexpectedly, conversion was also significantly reduced to 12% when the *isc* regulon repressor, *iscR,* was knocked out. Such genomic modifications should increase IscS expression and thus greater Ph_3_PS formation. However, the observed decrease is likely due to upregulation of other genes in the *isc* operon enabling greater sulfur flux through the *isc* persulfide relay toward [Fe–S] cluster formation and subsequently away from Ph_3_PS formation. While no other persulfides network knockout significantly impacted P = S formation, disrupting the major modes of cysteine import and sulfur export predictably decreased isolated Ph_3_PS yields (by 32% and 24% respectively) by altering l-Cys availability. While conversion was not entirely eliminated in any screened knockout, this was unsurprising given functional overlap and degeneracy in sulfane sulfur metabolism – particularly between IscS and SufS.^11^ Regardless these combined experiments clearly implicate the role of enzymatic l-Cys desulfuration in nonenzymatic P = S formation in bacteria.

Intriguingly, enzyme-bound persulfides may not be the only route through which Ph_3_PS formation may occur. A major metabolic product from l-Cys utilization and degradation is H_2_S.^12,13^ Similarly, metabolic persulfides also degrade to H_2_S *in vivo*,^14^ meaning l-Cys supplemented *E. coli* cultures are rich in gaseous hydrogen sulfide. Interestingly, reactions of H_2_S with O_2_ can also generate sulfane sulfurs via SO_2_ under mild conditions.^15^ We therefore hypothesized that enzymatically derived H_2_S may also serve as a source of abiotic P = S formation. This was confirmed by collecting headspace H_2_S from an active *E. coli* BW25113 culture into a secondary oxygenated reaction vessel and demonstrating Ph_3_PS conversion *in vitro* – supporting the role of H_2_S in P = S formation (Figure S17).

As H_2_S readily diffuses through the cell envelope, O_2_-dependent sulfane sulfur formation and subsequently P = S bond formation may occur *ex vivo*. By adding Ph_3_P to spent culture supernatant isolated from cells grown under analogous conditions in the presence of 50 mM l-Cys, we observed an 8-fold increase in Ph_3_PS formation compared to *E. coli* cultures without l-Cys supplementation. However, this was reduced compared to the 12.5-fold increase observed in actively growing *E. coli* cultures (Figure S18). In contrast, minimal product conversion was detected when the washed cell pellet was incubated with Ph_3_P, confirming the role of secreted sulfur in P = S bond formation. By sparging then reoxygenating cultures prior to the addition of Ph_3_P, Ph_3_PS conversion was reduced by 58%. Sparging replaces both dissolved and headspace gas whereas reoxygenation degrades dissolved sulfane sulfur through reactive oxygen species chemistry.

Results here further demonstrate that H_2_S forms a key sulfur source that can react with Ph_3_P *ex vivo* via sufane sulfur formed by oxidation during the reaction. Although not all of the original species from the NCIMB curation shown to produce Ph_3_PS are known to produce H_2_S, sulfane sulfur remains a key intermediate in [Fe–S] clusters^16^ and tRNA synthesis^17,18^ which are ubiquitous in biology thus we hypothesize elevated concentrations of such reactive sulfur sources (e.g., due to greater cysteine desulfurase expression) are responsible for the detectable conversion observed in these strains. Given the ubiquity of IscS and SufS homologues in microbiology, low-level P = S bond formation may be more widespread than shown here as detected by ^31^P NMR spectroscopy and development of a sensitive Ph_3_PS analysis technique may enable a simple means of detecting metabolically active cells and P = S bond formation *in vivo*. Such a system would be analogous to reported detection methods via high-performance liquid chromatography^19^ and isotope dilution mass spectrometry-based.^20^

**Figure 4:**
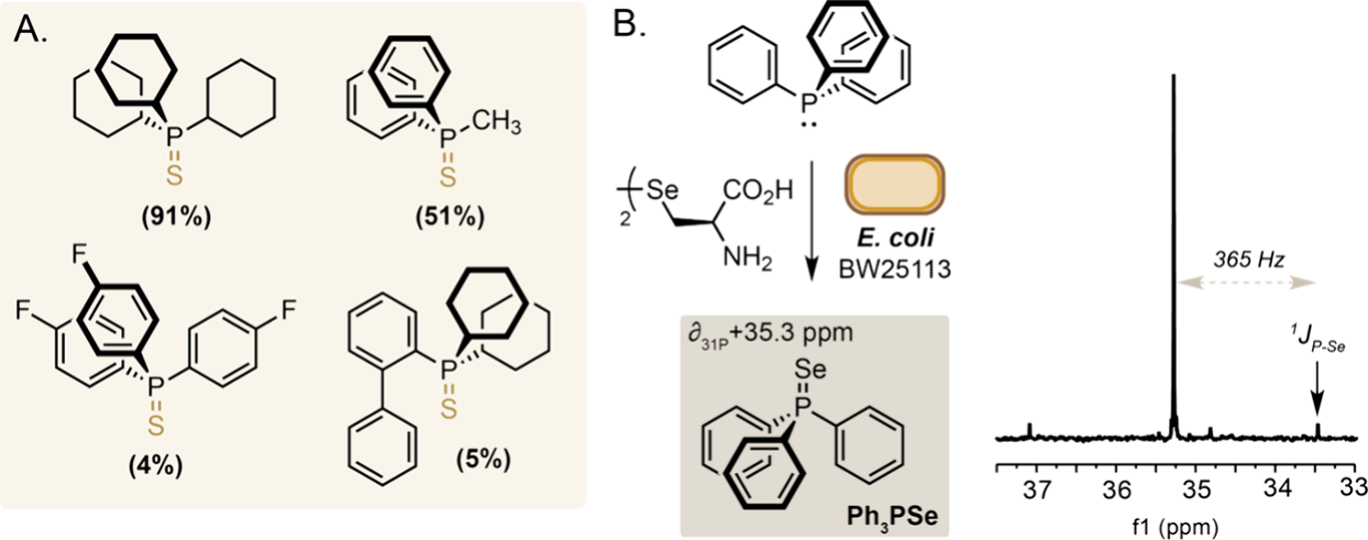
A) Microbial P = S formation using various trialkyl and aryl phosphines. B) Ph_3_PSe formation by *E. coli* BW25113 incubated in the presence of Sec_2_ and triphenylphosphine detected by ^31^P NMR spectroscopy. A 730 Hz^1^J_31P-77Se_ coupling is observed confirming *P* = Se bond formation. Reaction conditions: Sec_2_ (25 mM) added to culture of *E. coli* BW25113_pET28a(+) (OD_600_ 0.4–0.6) in LB media and incubated at 37 °C (220 rpm) for 44 h before extraction using ethyl acetate and analysis by ^31^P NMR spectroscopy.

Having shown P = S formation in *E. coli* was derived from amino acid metabolism via sulfane sufur and H_2_S biosynthesis, we moved on to examine microbial P = S and P = X formation using other P(III) substrates. Under optimized reaction conditions using *E. coli* BW25113 cultures supplemented with 50 mM l-Cys we observed formation of tricyclohexylphosphine sulfide from tricyclohexylphosphine in 91% yield, and methyldiphenylphosphine sulfide from methyldiphenylphosphine in 51% yield (Figure 4A; Figures S19–S22). *Tris*(4-fluorophenyl)phosphine showed low reactivity, with <5% conversion to the corresponding phosphine sulfide likely resulting from decreased reactivity of the P(III) nucleophile due to electronic effects. Interestingly, despite high reactivity of both triphenylphosphine and tricyclohexylphosphine, 2-(dicyclohexylphosphino)biphenyl (CyJohnPhos), was poorly reactive, with only 5% conversion to the corresponding phosphine sulfide observed under the reactions conditions. Although the exact reasons(s) for this are currently unclear, elevated steric hindrance at the P(III) atom in addition to increased delocalization of the phosphorus lone-pair of electrons could explain the low reactivity of this substrate.

Finally, cysteine desulfurases have also been reported to liberate selenide from selenocysteine.^21^ Therefore, we hypothesized that degeneracy in this enzymatic chemistry could also translate to phosphine selenide and P = Se bond formation in bacteria under our optimized reaction conditions. Indeed, addition of selenocystine (Sec_2_) resulted in an additional peak at δ + 35.3 ppm in the ^31^P NMR spectrum corresponding to triphenylphosphine selenide as indicated by comparison to literature^22^ and a large observed 730 Hz^1^J_P–Se_ coupling constant (Figure 4B; Figure S23). To the best of our knowledge, this marks the first example of P = Se bond formation by a bacterial metabolic pathway.

## Conclusions

In conclusion, diverse bacteria have been found to promote nonenzymatic P = S bond formation through the generation of reactive sulfur metabolites derived from l-Cys. This microbial chemistry was found in 11 distinct species within an uncharacterized national culture collection and can also be observed and optimized to occur in preparative yields and on a panel of substrates using laboratory strains of *Escherichia coli*. Genetic and biochemical studies indicate the source of reactive sulfur is multifaceted and likely stems from metabolic H_2_S formation and sulfane sulfur generation under aerobic conditions, followed by abiotic P = S bond formation. This principle can also be applied to form P = Se bonds *in vivo* when *E. coli* is provided Sec_2_. Overall, this work highlights the diverse metabolic chemistry of microorganisms and how microbial metabolism can be coerced and applied to create new synthetic methods for sustainable chemical synthesis.

## Supporting Information Available

Supplementary data, general materials and methods, species, strain and culturing conditions, ^31^P NMR characterization data (PDF)
