# Supplementary material for: Microbially Derived P=S and P=Se Bond Formation
Source: JACS Au. 2025 Mar 29;5(4):2027–32. doi: 10.1021/jacsau.5c00262 (PMC12042036; doi:10.1021/jacsau.5c00262)
Supplement: Supplementary file 1 — au5c00262_si_001.pdf [file au5c00262_si_001.pdf]

Supplementary Information

Microbially derived P=S and P=Se bond formation

Connor L. Trotter[1], Yuta Era[1], Rory Gordon[1,2], Samantha Law[3], Christopher Switzer[4] and Stephen Wallace[1*]

[1]Institute of Quantitative Biology, Biochemistry and Biotechnology, School of Biological Sciences, University of Edinburgh, Edinburgh, EH9 3FF, UK

[2] EaStCHEM School of Chemistry, University of Edinburgh, Edinburgh, EH9 3FJ, UK

[3]NCIMB Ltd., Wellheads Place, Dyce, Aberdeen, AB21 7GB, UK

[4]Department of Molecular and Cell Biology, University of Leicester, Leicester, LE1 7RH, UK

*Email: stephen.wallace@ed.ac.uk

Table of Contents

S1 Species, strains, and culturing conditions ______ *3*

S1.1 NCIMB Species ______ 3

S1.2 *Escherichia coli* strains ______ 5

S1.3 Growth Media ______ 5

S1.4 Culture conditions ______ 6

S1.5 Phylogenetic Tree Construction ______ 6

S2 Experimental Methods ______ *7*

S2.1 Initial triphenylphosphine oxide screen ______ 7

S2.2 *Escherichia coli* phosphine sulfide screens ______ 7

S2.3 *Escherichia coli* sulfane sulfur measurements ______ 8

S2.4 *In vitro* reactions between sulfane sulfur and triphenylphosphine ______ 8

S2.5 Separated *E. coli* culture screens ______ 8

S3 Supplementary Figures ______ *10*

S3.1 Phylogenetic analysis of 16s rRNA gene in curated NCIMB collection ______ 10

S3.2 ^{31}P NMR standards ______ 27

S3.3 Triphenylphosphine oxide reduction ^{31}P NMR traces ______ 28

S3.4 Triphenylphosphine modification ^{31}P NMR traces ______ 31

S3.5 Effect of sulfur metabolism on P=S formation in *E. coli* ______ 36

S3.6 Identifying the interactions between biotic and abiotic P=S formation ______ 38

S4 Phosphine Substrate Scope ______ *41*

S5 References ______ *45*

All starting materials and reagents were obtained from commercial suppliers and were used without further purification. Additionally, all water used experimentally was purified with a Suez Select purification system (18 MΩ.cm, 0.2 μM filter). All steps completed under sterile conditions using sterilised equipment. The following abbreviations are used throughout: triphenylphosphine (Ph_3P), triphenylphosphine oxide (Ph_3PO), triphenylphosphine sulfide (Ph_3PS), cyclohexyldiphenylphosphine oxide ($CyPh_2PO$) L-cysteine (L-Cys), L-cystine (Cys_2), L-methionine (L-Met), L-selenocystine (Sec_2) and hydrogen sulfide (H_2S).

S1 Species, strains, and culturing conditions

S1.1 NCIMB Species

Table S1. List of bacterial species used in this work including respective growth conditions and isolation locations as given by NCIMB Ltd..

Species name	NCIMB Accession	Temp (°C)	Media	Isolated from
Shimwellia pseudoproteus	NCIMB 14534	30	Lab8	Brewing contaminant, Scotland
Cupriavidus metallidurans	NCIMB 14178	30	TSB	Decantation tank of zinc factory, Belgium
Shewanella oneidensis	NCIMB 14063	30	TSB	Lake sediment, Japan
Rhodococcus corynebacterioides	NCIMB 9433	30	NB	Air contaminant of culture medium
Lysinibacillus sphaericus	NCIMB 9370	30	TSB	Unknown
Priestia megaterium	NCIMB 9376	30	NB	Unknown
Staphylococcus delphini	NCIMB 13206	30	Lab8	Purulent material of a dolphin in captivity
Alkalihalobacillus gibsonii	NCIMB 11495	30	NB	Pots of dune sand containing marram grass, although possibly lab contaminant, Wales
Rhodanobacter spathiphylli	NCIMB 14290	30	TSB	Rhizospheres of *Spathiphyllum* plants grown on a compost-amended potting mix
Bacillus subtilis	NCIMB 15199	30	NB	Rhizosphere of wild plants
Iodobacter fluviatile	NCIMB 2053	25	¼ NB	River Wey, England
Sphingomonas roseiflava	NCIMB 14155	25	NB	Ears of plants in the family *Gramineae roseiflava*
Paenarthrobacter aurescens	NCIMB 8912	25	NB	Unknown

Phyllobacterium myrsinacearum	NCIMB 12127	25	NB	Leaf nodule of *Ardisia crispa* in tropical greenhouse
Cellulosimicrobium cellulans	NCIMB 11440	25	NB	Soil
Marinobacter nauticus	NCIMB 1967	25	SW	Sea water by enrichment on acetate + nitrate, coast of Oahu, Hawaii, U.S.A.
Kurthia sibirica	NCIMB 13254	25	TSB	Large intestine of mammoth, Russia
Agrococcus versicolor	NCIMB 14817	25	TYSE	Phyllosphere of potato plants, Germany
Kitasatospora herbaricolor	NCIMB 9837	25	ISP2	Soil, Nishi Park, Fukuoka City, Japan
Photorhabdus luminescens	NCIMB 12670	25	BB	Nematode worm (*Heterorhabditis bacteriophora*)
Gordonia alkanivorans	NCIMB 13615	25	NB	Tar and phenol contaminated soil, Germany
Nocardioides daphniae	NCIMB 14676	25	TSB	*Daphnia cucullata*, Hugary
Morganella psychrotolerans	NCIMB 14755	30	TSB	Cold smoked tuna, Denmark
Gluconacetobacter tumulisoli	NCIMB 14861	30	YG	Clay soil, Japan
Streptomyces thermoviolaceus	NCIMB 10076	45	ISP2	Mixed fresh horse and swine manure, Germany
Microbacterium esteraromaticum	NCIMB 8748	25	NB	Foul brood of bees
Planococcus kocurii	NCIMB 628	20	SW	Skin of North Sea cod, UK
Psychrobacter okhotskensis	NCIMB 28	20	BB	Skin of Cod
Pseudomonas fluorescens	NCIMB 129	30	NB	Skin of Cod, Norway
Ligilactobacillus equi	NCIMB 14042	37	MRS	Horse faeces, Japan
Amycolatopsis lurida	NCIMB 9378	30	ISP2	Intestines of *Apis mellifera*
Pseudonocardia thermophila	NCIMB 10079	45	ISP2	Horse manure, Germany
Metabacillus galliciensis	NCIMB 14735	30	NB	Wild seahorse faeces, Spain
Herbaspirillum seropedicae	NCIMB 12540	30	NB	Rhizosphere of rice plants

S1.2 *Escherichia coli* strains

Table S2. List of *E. coli* strains used in this work. *Alternative gene names given as appropriate.*

Strain name	Genotype	Source
E. coli BW25113 pET28b	BD792(F- LAM- rrnB3 DElacZ4787 hsdR514 DE(araBAD)567 DE(rhaBAD)568 rph-1) pET28b	This study
E. coli JW2514	*E. coli* BW25113 Δ*iscS*	Horizon Discovery
E. coli JW2515	*E. coli* BW25113 Δ*iscR*	Horizon Discovery
E. coli JW5287	*E. coli* BW25113 Δ*ynjE*	Horizon Discovery
E. coli JW1670	*E. coli* BW25113 Δ*sufS*	Horizon Discovery
E. coli JW2781	*E. coli* BW25113 Δ*csdA*	Horizon Discovery
E. coli JW0413	*E. coli* BW25113 Δ*thiI*	Horizon Discovery
E. coli JW3455	*E. coli* BW25113 Δ*tusA* (/Δ*yhhp*)	Horizon Discovery
E. coli JW2505	*E. coli* BW25113 Δ*sseA*	Horizon Discovery
E. coli JW1301	*E. coli* BW25113 Δ*pspE*	Horizon Discovery
E. coli JW5250	*E. coli* BW25113 Δ*eamA*	Horizon Discovery
E. coli JW1301	*E. coli* BW25113 Δ*tcyP* (/Δ*ydjN*)	Horizon Discovery
E. coli JW2975	*E. coli* BW25113 Δ*metC*	Horizon Discovery

S1.3 Growth Media

All media were made to volume using MilliQ ddH_2O and sterilised by autoclave at 121 °C for 20 min. When solid media was required, agar (15 g/L; Formedium) was added prior to autoclave sterilisation. After autoclave, media was stored at room temperature.

Luria-Bertani Broth (LB): Tryptone (10 g/L; Sigma-Aldrich), NaCl (10 g/L; Fisher Scientific), and yeast extract (5 g/L; Sigma-Aldrich).

Nutrient Broth (NB): Beef extract (1 g/L; Sigma-Aldrich), yeast extract (2.0 g/L; Sigma-Aldrich), casein peptone (5 g/L; Sigma-Aldrich), and NaCl (5.0 g/L; Fisher Scientific).

Tryptone Soy Broth (TSB): Tryptone (15 g/L; Sigma-Aldrich), soya peptone (5.0 g/L; Oxoid), and NaCl (5.0 g/L; Fischer Scientific).

International Streptomyces Project Media 2 (ISP2): Yeast extract (4.0 g/L; Sigma-Aldrich), malt extract (10.0 g/L; Fisher Scientific), *D*-glucose (4.0 g/L; Sigma-Aldrich). For solid agar, 20 g/L agar was added instead.

Lab8 Broth: Casein peptone (5.0 g/L; Sigma-Aldrich), beef extract (3.0 g/L; Sigma-Aldrich), and NaCl (8.0 g/L; Fisher Scientific).

Sea Water (SW) Broth: Aged Sea Water (750 mL/L; collected from South Queensferry, Scotland (coordinates: 55.9921188, -3.3862419) on 17/04/2021, aged (3 month, dark, room temperature) then filtered (0.45 μm) and stored (dark, room temperature) until needed) was combined with ddH$_2$O (250 mL/L) and thoroughly mixed (30 minutes). To promote growth, beef extract (10.0 g/L; Sigma-Aldrich) and casein peptone (10.0 g/L; Sigma-Aldrich) were dissolved prior to autoclave sterilisation. All species also grew in Marine 2216 media (Fisher Scientific); however, this was not used for screening.

De Man, Rogosa and Sharpe (MRS) Broth: Dehydrated MRS powder (52 g/L; premade by Oxoid).

Tryptone Soya Yeast Extract (TSYE) Broth: Tryptone (15 g/L; Sigma-Aldrich), soya peptone (5 g/L; Oxoid), NaCl (5.0 g/L; Fisher Scientific), K_2HPO_4 (2.5 g/L;), and yeast extract (3 g/L; Sigma-Aldrich).

Blood Base (BB) Broth: Beef extract (10 g/L; Sigma-Aldrich), casein peptone (10 g/L; Sigma-Aldrich), and NaCl (5 g/L; Fisher Scientific). All species also grew in NB media; however, this was not used for screening.

Yeast Glucose (YG) Broth: *D*-glucose (20 g/L; Sigma-Aldrich) and yeast extract (4.0 g/L; Sigma-Aldrich).

S1.4 Culture conditions

Overnight culturing of all NCIMB species was performed at 10 mL scale under optimal growth conditions as identified by NCIMB Ltd. (Supplementary Table S1). Cultures were inoculated from a single colony grown on solid media to ensure axenic culturing. *E. coli* strains were inoculated directly from glycerol stocks stored at –70 °C into 10 mL LB containing 50 μg/mL kanamycin unless specified.

For day culturing, overnight cultures were diluted 1:100 into fresh media and incubated under optimal conditions until desired optical density was obtained. All measurements of cell density were determined by absorbance at 600 nm (OD_{600}) using a DS-11 UV/Vis spectrophotometer (DeNovix) blanked with the corresponding liquid media.

S1.5 Phylogenetic Tree Construction

Relationships between species was determined by constructing a 16s rRNA phylogenetic tree using NGPhylogeny.fr[1] and Mega11.[2] Briefly, 16s rRNA sequences (Supplementary Table S3) for the type strain of each species was obtained from the List of Prokaryotic names with Standing in Nomenclature (LPSN[3]; accessible at https://lpsn.dsmz.de)

and aligned using the Multiple Alignment using Fast Fourier Transform (MAFFT) algorithm[4]. Alignment was curated using the Block Mapping and Gathering with Entropy (BMGE) tool[5] to improve the quality of alignment prior to tree construction. The best fitting model for tree construction was identified as General Time Reversible with gamma distributed rates and invariable sites (GTR +G +I) using Mega11. The Maximum Likelihood phylogenetic tree was then inferred using NGPhylogeny.fr (accessible at https://ngphylogeny.fr) with a bootstrap of 500 pseudoreplicates. The inferred Newick tree was then rendered using TreeViewer 4.0[6]. *Chlamydia trachomatis* was used as an outgroup to infer relation. All nodes with <70% bootstrap support were collapsed and accuracy of the inferred tree was confirmed by mapping phyla.

S2 Experimental Methods

S2.1 Initial triphenylphosphine oxide screen

To screen for the reduction of Ph_3PO to Ph_3P, day cultures (50 mL) were grown in 250 mL Erlenmeyer flasks until the culture was established (30-40% final optical density at 600 nm). Once sufficiently grown, culture was aliquot (10 mL) into sterile 15 mL Hungate tubes containing 3 mM Ph_3PO or Ph_3P and sealed with a sterile butyl rubber stopper and screwcap. Where required, anaerobic cultures were generated by bubbling oxygen-free N_2 (BOC) through the culture (~0.5 mL/sec; 15 min) using a 12-inch needle as the inlet and a 25-gauge, 5/8-inch needle as the outlet. Cultures were incubated horizontally to improve mixing (220 rpm, 44 h).

After incubation, samples were worked up for ^{31}P NMR analysis. Briefly, cultures were extracted with ethyl acetate (2x 10 mL) and the organic phase was concentrated *in vacuo* (35 °C, 200 rpm, 115-125 mbar, 15 min or until dry). Dried sample was resuspended in $CDCl_3$ and analysed by H^+-decoupled ^{31}P NMR using a Bruker Ava400 (all NCIMB species) or Pro500 (all *E. coli* work).

S2.2 *Escherichia coli* phosphine sulfide screens

Characterisation of phosphine sulfide formation was completed using *E. coli* BW25113 and derivative knockout strains. Screens were completed under aerobic conditions as described above using *E. coli* cultures at OD_{600} 0.4-0.6 unless specified. All phosphine substrates were added at 3 mM. Samples were also supplemented with 50 mM L-Met, L-Cys or 25 mM Cys_2, L-Sec as required during the sample aliquoting stage. During sample workup for ^{31}P NMR spectroscopy analysis, an internal standard (30 mM $CyPh_2PO$) was included to enable accurate quantification of reaction products.

S2.3 *Escherichia coli* sulfane sulfur measurements

Overnight cultures of *Escherichia coli* BW25113 were produced by inoculating 5 mL LB media containing 50 μg/mL kanamycin with a single colony. Cultures were incubated (37 °C, 220 rpm, 16 h) then diluted to OD_{600} 0.4-0.6. Resulting culture was aliquot (5 mL) and supplemented with 50 mM L-Met, L-Cys or 25 mM Cys_2 then incubated (37 °C, 220 rpm, 41 h). To prepare cells for fluorescent measurements, culture (100 μL) was washed three times with an equal volume phosphate buffered saline then resuspended in PBS containing the fluorescent probes SSP4 (20 μM; Dojindo) and Hoechst 33342 (2 μg/mL; Invitrogen). Resuspended cells were then incubated (37 °C, 200 rpm, 1 h) to enable probe uptake then washed in PBS twice again to removed excess probes. Final washed cultures were then incubated (37 °C, 200 rpm, 2 h) to enable probe binding.

For measurement, cell suspensions were aliquoted into black 96-well plates and fluorescence intensity (SSP4: 485/520 nm; Hoechst 33342: 360/465 nm) measured at 37 °C with a CLARIOstar Plus plate reader (BMG Labtech). SSP4 fluorescence was normalised to Hoechst 33342 fluorescence and data were then normalised to control mean to calculate fold changes. Data shown represent normalised mean values of 4 biological replicates (± standard error of the mean) and measured as technical duplicates.

S2.4 *In vitro* reactions between sulfane sulfur and triphenylphosphine

Reactivity between biologically relevant sulfane sulfur and triphenylphosphine was confirmed *in vitro.* For this, 10 mM Ph_3P (100 μL; aq. 100% acetone) and either 20 mM L-Cys (100 μL; aq. 100% H_2O) or 10 mg/mL K_2S_x (100 μL; aq. 100% H_2O) were mixed in equal volume. Alternatively, 20 mM Ph_3P (100 μL; aq. 100% acetone) and 2 mM S_8 (300 μL; aq. H_2O) were reacted at 60 °C for 30 minutes with agitation. All reactions were cooled to 4 °C, then $CHCl_3$ (200 μL) and H_2O (100 μL) were added, vortexed (3 x 5 seconds) and centrifuged (15,000 x *g*, 5 minutes, room temperature). The lower fraction was removed, mixed with approximately equal volume $CDCl_3$ and analysed by ^{31}P-NMR.

S2.5 Separated *E. coli* culture screens

To A) investigate the role of H_2S in Ph_3PS formation and B) to distinguish between enzymatic and biologically derived non-enzymatic Ph_3PS formation, two-stage culturing and reaction approachs were taken. For the initial sulfur accumulation stage, *E. coli* BW25113

cultures were inoculated 1% (v/v) from overnight cultures and incubated (37 °C, 220 rpm) to desired cell density (OD_{600} 0.4-0.6). Upon sufficient growth, culture was aliquoted (10 mL) into 15 mL Hungate tubes containing 50 mM L-Cys or 25 mM Cys_2 then sealed with a butyl rubber stopper and incubated (37 °C, 220 rpm, 44 h).

After incubation, cultures were prepared for the reaction stage. To screen for H_2S reactivity, headspace gas (5 mL) was drawn into a 20 mL rubber stoppered syringe containing LB media (10 mL) with 250 μg/mL kanamycin, 250 μg/mL streptomycin, 500 μg/mL carbenicillin and 125 μg/mL chloramphenicol (to prevent growth). Headspace gas was drawn up using a sterile 25G needle and 0.22 μm filter to prevent contamination by *E. coli* cultures. Syringes were capped with a Luer lock to reduce H_2S loss then incubated (37 °C, 220 rpm, 44 h).

Similarly, to screen the impact of secreted sulfur metabolites on reactivity, cultures were transferred into sterile 50 mL Falcon tubes and clarified by centrifugation (3,250 *g*, 15 min, 4 °C). The resulting supernatant was transferred into a new sterile Falcon tube. To prevent recovery of planktonic *E. coli* cells, the supernatant was filtered (0.22 μm) thrice and then supplemented with four antibiotics as above. Cell pellet produced during clarification was washed thrice with two volumes (20 mL) phosphate buffered saline then resuspended in the original volume of fresh LB broth containing 50 μg/mL kanamycin. Washed cell pellet, treated supernatant, and a whole culture control were aliquoted (10 mL) into sterile 15 mL Hungate tubes containing 3 mM Ph_3P and sealed with a butyl rubber stopper and screw cap. Dissolved H_2S was removed by bubbling the cultures with oxygen-free N_2 as previous. Oxygen was reintroduced to the cultures by bubbling through 250 mL air (~0.5 mL/min) and then cultures were incubated horizontally (37 °C, 220 rpm, 44 h). Sterility of samples was confirmed after incubation by inoculating solid LB media with sample (100 μL) prior to further processing. Resulting plates were incubated (37 °C, 24 h) and growth was monitored. Samples for both screens were then worked up and prepared for quantitative ^{31}P NMR as described in Section S2.1.

S3 Supplementary Figures

S3.1 Phylogenetic analysis of 16s rRNA gene in curated NCIMB collection

Table S3. Representative type strain 16s rRNA sequences of curated NCIMB collection. All sequences obtained from the List of Prokaryotic Names with Standing in Nomenclature database (LPSN; available at https://lpsn.dsmz.de). Date of access 01/01/2025.

Species	16s rRNA sequence
Shimwellia pseudoproteus	AACACATGCAAGTCGAGCGGCAGCGGGAAGTAGCTTGCTACTTTGCCGGCGA<br>GCGGCGGACGGGTGAGTAATGTCTGGGGAACTGCCTGATGGAGGGGGATAA<br>CTACTGGAAACGGTAGCTAATACCGCATAACGTCGCAAGACCAAAGTGGGGG<br>ACCTTCGGGCCTCATGCCATCAGATGTACCCAGATGGGATTAGCTAGTAGGTG<br>GGGTAACGGCTCACCTAGGCGACGATCCCTAGCTGGTCTGAGAGGATGACCA<br>GCCACACTGGAACTGAGACACGGTCCAGACTCCTACGGGAGGCAGCAGTGG<br>GGAATATTGCACAATGGGCGCAAGCCTGATGCAGCCATGCCGCGTGTATGAA<br>GAAGGCCTTCGGGTTGTAAAGTACTTTCAGCGAGGAGGAAGGTGTTGTGGTTA<br>ATAACCACAGCAATTGACGTTACTCGCAGAAGAAGCACCGGCTAACTCCGTGC<br>CAGCAGCCGCGGTAATACGGAGGGTGCAAGCGTTAATCGGAATTACTGGGCG<br>TAAAGCGCACGCAGGCGGTTGATTAAGTCAGATGTGAAATCCCCGGGCTTAAC<br>CTGGGAACTGCATTTGAAACTGGTCAGCTTGAGTCTCGTAGAGGGGGGTAGA<br>ATTCCAGGTGTAGCGGTGAAATGCGTAGAGATCTGGAGGAATACCGGTGGCG<br>AAGGCGGCCCCCTGGACGAAGACTGACGCTCAGGTGCGAAAGCGTGGGGAG<br>CAAACAGGATTAGATACCCTGGTAGTCCACGCCGTAAACGATGTCAACTTGGA<br>GGTTGTGGTCTTGAACCGTGGCCTCCGGAGCTAACGCGTTAAGTTGACCGCC<br>TGGGGAGTACGGCCGCAAGGTTAAAACTCAAATGAATTGACGGGGGCCCGCA<br>CAAGCGGTGGAGCATGTGGTTTAATTCGATGCAACGCGAAGAACCTTACCTGG<br>TCTTGACATCCATAGAACTTGGCAGAGATGCCTTGGTGCCTTCGGGAGCTATG<br>AGACAGGTGCTGCATGGCTGTCGTCAGCTCGTGTTGTGAAATGTTGGGTTAAG<br>TCCCGCAACGAGCGCAACCCCTATCCTCTGTTGCCAGCACTTCGGGTGGGAA<br>CTCAGGGGAGACTGCCAGTGATAAACTGGAGGAAGGTGGGGATGACGTCAAG<br>TCATCATGGCCCTTACGACCAGGGCTACACACGTGCTACAATGGCGCATACAA<br>AGAGAAGCTACCTCGCGAGAGCAAGCGGATCTCATAAAGTGCGTCGTAGTCC<br>GGATTGGAGTCTGCAACTCGACTCCATGAAGTCGGAATCGCTAGTAATCGTGG<br>ATCAGAATGCCACGGTGAATACGTTCCCGGGCCTTGTACACACCGCCCGTCA<br>CACCATGGGAGTGGGTTGCAAAAGAAGTAGGTAGCTTAACCTTCGGGAGGGC<br>GCTTACCACTT
Cupriavidus metallidurans	AGAGTTTGATCCTGGCTCAGATTGAACGCTGGCGGTATGCCTTACACATGCAA<br>GTCGAACGGCAGCGCGGACTTCGGTCTGGCGGCGAGTGGCGAACGGGTGAG<br>TAATACATTGGAACGTACCCTGTTGTGGGGGATAACTAGTCGAAAGATTAGCT<br>AATACCGCATACGACCTGAGGGTGAAAGTGGGGGACCGCAAGGCCTCACGCA<br>GCAGGAGCGGCCGATGTCTGATTAGCTAGTTGGTGGGGTAAAGGCCCACCAA<br>GGCGACGATCAGTAGCTGGTCTGAGAGGACGATCAGCCACACTGGGACTGAG<br>ACACGGCCCAGACTCCTACGGGAGGCAGCAGTGGGGAATTTTGGACAATGGG<br>GGCAACCTGTATCCAGCAATGCCGCGTGTGTGAAGAAGGCCTTCGGGTTGTA<br>AAGCACTTTTGTCCGGAAAGAAATCGCGCTGGTTAATACCTGGCGTGGATGAC<br>GGTACCGGAAGAATAAGCACCGGCTAACTACGTGCCAGCAGCCGCGGTAATA<br>CGTAGGTGCGAGCGTTAATCGGAATTACTGGGCGTAAAGCGTGCGCAGGCGG<br>TTTTGTAAGACAGGCGTGAAATCCCCGGGCTTAACCTGGGAATTGCGCTTGTG<br>ACTGCAAGGCTAGAGTGCGTCAGAGGGGGGTAGAATTCCACGTGTAGCAGTG<br>AAATGCGTAGAGATGTGGAGGAATACCGGATGGCGAAGGCAGCCCCCTGGGA<br>CGTGACTGACGCTCATGCACGAAAGCGTGGGGAGCAAACAGGATTAGATACC<br>CTGGTAGTCCACGCCCTAAACGATGTCAACTAGTTGTTGGGGATTCATTTTCTC<br>AGTAACGATACTAACGCGTGAAGTTGACCGCCTGGGGAGTACGGTCGCAAGA<br>TTAAACTCAAAGGAATTGAGGGGACCCGCACAAGCGGTGGATGATGTGGATTA<br>ATTCGATGCAACGCGAAAAACCTTACCTACCCTTGACATGCCACTAACGAAGC<br>AGAGATGCATTAGGTGCCCGAAAGGGAAAGTGGACACAGGTGCTGCATGGCT<br>GTCGTCAGCTCGTGTCGTGAGATGTTGGGTTAAGTCCCGCAACGAGNGCAAC<br>CCTTGTCTCTAGTTGCTACGCAAGAGCACTCTAGAGAGACTGCCGGTGACAAA<br>CCGGAGGAAGGTGGGGATGACGTCAAGTCCTCATGGCCCTTATGGGTAGGGC<br>TTCACACGTCATACAATGGTGCGTACAGAGGGTTGCCAACCCGCGAGGGGGA

	GCTAATCCCAGAAAACGCATCGTAGTCCGGATCGTAGTCTGCAACTCGACTAC<br>GTGAAGCTGGAATCGCTAGTAATCGCGGATCAGCATGCCGCGGTGAATACGT<br>TCCCGGGTCTTGTACACACCGCCCGTCACACCATGGGAGTGGGTTTTGCCAG<br>AAGTAGTTAGCCTAACCGCAAGGAGGGCGATTACCACGGCAGGGTTCATGAC<br>TGGGGTGAAGTCGTAACAAGGTAGCCGT
Shewanella oneidensis	GATCTGCCCAGTCGAGGGGGATAACAGTTGGAAACGACTGCTAATACCGCATA<br>CGCCCTACGGGGGAAAGAGGGGGACTTTCGGGCCTCTCGCGATTGGATGAAC<br>CTAGGTGGGATTAGCTAGTTGGTGAGGTAATGGCTCACCAAGGCGACGATCC<br>CTAGCTGTTCTGAGAGGATGATCAGCCACACTGGGACTGAGACACGGCCCAG<br>ACTCCTACGGGAGGCAGCAGTGGGGAATATTGCACAATGGGGGAAACCCTGA<br>TGCAGCCATGCCGCGTGTGTGAAGAAGGCCTTCGGGTTGTAAAGCACTTTCA<br>GTAGGGAGGAAAGGGTAANTCCTAATACGNCTTATCTGTGACGTTACCTACAG<br>AAGAAGGACCGGCTAACTCCGTGCCAGCAGCCGCGGTAATACGGAGGGTCCN<br>AGCGTTAATCGGAATTACTGGGCGTAAAGCGTGCGCAGGCGGTTTGTTAAGC<br>GAGATGTGAAAGCCCTGGGCTCAACCTAGGAATCGCATTTCGAACTGACCAAC<br>TAGAGTCTTGTAGAGGGGGGTAGAATTCCAGGTGTAGCGGTGAAATGCGTAG<br>AGATCTGGAGGAATACCGGTGGCGAAGGCGGCCCCCTGGACAAAGACTGAC<br>GCTCATGCACGAAAGCGTGGGGAGCAAACAGGATTAGATACCCTGGTAGTCC<br>ACGCCGTAAACGATGTCTACTCGGAGTTTGGTGTCTTGAACACTGGGCTCTCA<br>AGCTAACGCATTAAGTAGACCGCCTGGGGAGTACGGCCGCAAGGTTAAAACT<br>CAAATGAATTGACGGGGGCCCGCACAAGCGGTGGAGCATGTGGTTTAATTCG<br>ATGCAACGCGAAGAACCTTACCTACTCTTGACATCCACGGAAGACTGCAGAGA<br>TGCGGTTGTGCCTTCGGGAACCGTGAGACAGGTGCTGCATGGCTGTCGTCAG<br>CTCGTGTTGTGAAATGTTGGGTTAAGTCCCGCAACGAGCGCAACCCCTATCCT<br>TATTTGCCAGCACGTAATGGTGGGAACTCTAGGGAGACTGCCGGTGATAAACC<br>GGAGGAAGGTGGGGACGACGTCAAGTCATCATGGCCCTTACGAGTAGGGCTA<br>CACACGTGCTACAATGGCGAGTACAGAGGGTTGCAAAGCCGCGAGGTGGAGC<br>TAATCTCACAAAGCTCGTCGTAGTCCGGATTGGAGTCTGCAACTCGACTCCAT<br>GAAGTCGGAATCGCTAGTAATCGTGGATCAGAATGCCACGGTGAATACGTTCC<br>CGGGCCTTGTACACACCGCCCGTCACACCATGGGAGTGGGCTGCAAAAGAAG<br>TGGGTAGCTTAACCTTCGGGGGGGCGCTCACCACTTTGTGGTTCATGACTGG<br>GGTGAAGTCGTAACAAGGTAGCCGTAAAT
Rhodococcus corynebacterioides	GACGAACGCTGGCGGCGTGCTTAACACATGCAAGTCGAACGGTAAGGCCCTT<br>CGGGGTACACGAGTGGCGAACGGGTGAGTAACACGTGGGTGATCTGCCCTG<br>CACTTCGGGATAAGCCCGGGAAACTGGGTCTAATACCGGATAGGACTGCGCG<br>GTGCATGCCGTGTGGTGGAAAGATTTATCGGTGCAGGATGGGCCCGCGGCCT<br>ATCAGCTTGTTGGTGGGGTAATGGCCTACCAAGGCGACGACGGGTAGCCGGC<br>CTGAGAGGGCGACCGGCCACACTGGGACTGAGACACGGCCCAGACTCCTAC<br>GGGAGGCAGCAGTGGGGAATATTGCACAATGGGCGCAAGCCTGATGCAGCG<br>ACGCCGCGTGAGGGATGACGGCCTTCGGGTTGTAAACCTCTTTCAGCAGGGA<br>CGAAGCGCAAGTGACGGTACCTGCAGAAGAAGCACCGGCCAACTACGTGCCA<br>GCAGCCGCGGTAATACGTAGGGTGCAAGCGTTGTCCGGAATTACTGGGCGTA<br>AAGAGCTCGTAGGCGGTTTGTCGCGTCGTCTGTGAAAACCAGCAGCTCAACT<br>GTTGGCTTGCAGGCGATACGGGCAGACTTGAGTATTTCAGGGGAGACTGGAA<br>TTCCTGGTGTAGCGGTGAAATGCGCAGATATCAGGAGGAACACCGGTGGCGA<br>AGGCGGGTCTCTGGGAAATAACTGACGCTGAGGAGCGAAAGCGTGGGTAGC<br>GAACAGGATTAGATACCCTGGTAGTCCACGCCGTAAACGGTGGGCGCTAGGT<br>GTGGGTTTCCTTCCACGGGATCCGTGCCGTAGCTAACGCATTAAGCGCCCCG<br>CCTGGGGAGTACGGCCGCAAGGCTAAAACTCAAAGGAATTGACGGGGGCCC<br>GCACAAGCGGCGGAGCATGTGGATTAATTCGATGCAACGCGAAGAACCTTAC<br>CTGGGTTTGACATACACCGGAAAGCCGTGGAGACACGGCCCCCCTTGTGGTC<br>GGTGTACAGGTGGTGCATGGCTGTCGTCAGCTCGTGTCGTGAGATGTTGGGT<br>TAAGTCCCGCAACGAGCGCAACCCTTGTCCTGTGTTGCCAGCACGTAATGGTG<br>GGGACTCGCAGGAGACTGCCGGGGTCAACTCGGAGGAAGGTGGGGACGACG<br>TCAAGTCATCATGCCCCTTATGTCCAGGGCTTCACACATGCTACAATGGCCGG<br>TACAGAGGGCTGCGATACCGTGAGGTGGAGCGAATCCCTTAAAGCCGGTCTC<br>AGTTCGGATCGGGGTCTGCAACTCGACCCCGTGAAGTCGGAGTCGCTAGTAA<br>TCGCAGATCAGCAACGCTGCGGTGAATACGTTCCCGGGCCTTGTACACACCG<br>CCCGTCACGTCATGAAAGTCGGTAACACCCGAAGCCGGTGGCCTAACCCCTT<br>GTGGGAGGGAGCCGTCGAAGGTGGGATCGGCGATTGGGACGAAGTCGTAAC<br>AAGGTAGCCGTACCGGAAGGTGCGGCTGGATCACCTCCTTTCT

Lysinibacillus sphaericus	CCTGGCTCAGGACGAACGCTGGCGGCGTGCCTAATACATGCAAGTCGAGCGA<br>ACAGAGAAGGAGCTTGCTCCTTTGACGTTAGCGGCGGACGGGTGAGTAACAC<br>GTGGGCAACCTACCCTATAGTTTGGGATAACTCCGGGAAACCGGGGCTAATAC<br>CGAATAATCTCTTGTCCCTCATGGGACAATACTGAAAGACGGTTTCGGCTGTC<br>GCTATAGGATGGGCCCGCGGCGCATTAGCTAGTTGGTGAGGTAACGGCTCAC<br>CAAGGCAACGATGCGTAGCCGACCTGAGAGGGTGATCGGCCACACTGGGACT<br>GAGACACGGCCCAGACTCCTACGGGAGGCAGCAGTAGGGAATCTTCCACAAT<br>GGGCGAAAGCCTGATGGAGCAACGCCGCGTGAGTGAAGAAGGATTTCGGTTC<br>GTAAAACTCTGTTGTAAGGGAAGAACAAGTACAGTAGTAACTGGCTGTACCTT<br>GACGGTACCTTATTAGAAAGCCACGGCTAACTACGTGCCAGCAGCCGCGGTA<br>ATACGTAGGTGGCAAGCGTTGTCCGGAATTATTGGGCGTAAAGCGCGCGCAG<br>GTGGTTTCTTAAGTCTGATGTGAAAGCCCACGGCTCAACCGTGGAGGGTCATT<br>GGAAACTGGGAGACTTGAGTGCAGAAGAGGATAGTGGAATTCCAAGTGTAGC<br>GGTGAAATGCGTAGAGATTTGGAGGAACACCAGTGGCGAAGGCGACTATCTG<br>GTCTGTAACTGACACTGAGGCGCGAAAGCGTGGGGAGCAAACAGGATTAGAT<br>ACCCTGGTAGTCCACGCCGTAAACGATGAGTGCTAAGTGTTAGGGGGTTTCC<br>GCCCCTTAGTGCTGCAGCTAACGCATTAAGCACTCCGCCTGGGGAGTACGGT<br>CGCAAGACTGAAACTCAAAGGAATTGACGGGGGCCCGCACAAGCGGTGGAGC<br>ATGTGGTTTAATTCGAAGCAACGCGAAGAACCTTACCAGGTCTTGACATCCCG<br>TTGACCACTGTAGAGATATGGTTTTCCCTTCGGGGACAACGGTGACAGGTGGT<br>GCATGGTTGTCGTCAGCTCGTGTCGTGAGATGTTGGGTTAAGTCCCGCAACGA<br>GCGCAACCCTTGATCTTAGTTGCCATCATTTAGTTGGGCACTCTAAGGTGACT<br>GCCGGTGACAAACCGGAGGAAGGTGGGGATGACGTCAAATCATCATGCCCCT<br>TATGACCTGGGCTACACACGTGCTACAATGGACGATACAAACGGTTGCCAACT<br>CGCGAGAGGGAGCTAATCCGATAAAGTCGTTCTCAGTTCGGATTGTAGGCTGC<br>AACTCGCCTACATGAAGCCGGAATCGCTAGTAATCGCGGATCAGCATGCCGC<br>GGTGAATACGTTCCCGGGCCTTGTACACACCGCCCGTCACACCACGAGAGTT<br>TGTAACACCCGAAGTCGGTGAGGTAACCTTTTGGAGCCAGCCGCCGAAGGTG<br>GGATAGATGATTGGGGTGAAGTCGTAACAAGGTAGCCGTATCGGAAGGT
Priestia megaterium	CGTGCGCGCTATAATGCAAGTCGAGCGAACTGATTAGAAGCTTGCTTCTATGA<br>CGTTAGCGGCGGACGGGTGAGTAACACGTGGGCAACCTGCCTGTAAGACTGG<br>GATAACTTCGGGAAACCGAAGCTAATACCGGATAGGATCTTCTCCTTCATGGG<br>AGATGATTGAAAGATGGTTTCGGCTATCACTTACAGATGGGCCCGCGGTGCAT<br>TAGCTAGTTGGTGAGGTAACGGCTCACCAAGGCAACGATGCATAGCCGACCT<br>GAGAGGGTGATCGGCCACACTGGGACTGAGACACGGCCCAGACTCCTACGG<br>GAGGCAGCAGTAGGGAATCTTCCGCAATGGACGAAAGTCTGACGGAGCAACG<br>CCGCGTGAGTGATGAAGGCTTTCGGGTCGTAAAACTCTGTTGTTAGGGAAGAA<br>CAAGTACGAGAGTAAGCTGCGCTCGTACCCTTTGACGGTACCTAACCCCAGAG<br>AAAGCCACCGGCTAAACTACGTGCCCAGCAGCCGCGGTAATACGTAGGTGGC<br>AAGCGTTATCCCGGAATTATTGGGCGTAAAGCGCGCGCAGGCGGTTTCTTAAG<br>TCTGATGTGAAAGCCCACGGCTCAACCGTGGAGGGTCATTGGAAACTGGGGA<br>ACTTGAGTGCAGAAGAGAAAAGCGGAATTCCACGTGTAGCGGTGAAATGCGTA<br>GAGATGTGGAGGAACACCAGTGGCGAAGGCGGCTTTTTGGTCTGTAACTGAC<br>GCTGAGGCGCGAAAGCGTGGGGAGCAAACAGGATTAGATACCCTGGTAGTCC<br>ACGCCGTAAACGATGAGTGCTAAGTGTTAGAGGGTTTCCGCCCTTTAGTGCTG<br>CAGCTAACGCATTAAGCACTCCGCCTGGGGGAGTACGGTCGCAAGACTGAAA<br>CTCAAAGGAATTGACGGGGGCCCGCACAAGCGGGTGGAGCATGTTGGTTTAA<br>TTCGAAGCAACGCGAAGAACCTTACCAGGTCTTGACATCCCTCTGACAACTC<br>TAGAAGATAAGAGCGGTTTCCCCTTCGGGGGGACAGAAGTGACCAGGTGGGT<br>GCCATGGGTGTCCGTCAGCCTCTGGTCCTGCGAAGGTTGGGTTAAGTCCCGC<br>AACGAGCGCAACCCTTGATCTTAGTTGCCAGCATTTAGTTGGGCACTCTAAGG<br>TGACTGCCGGTGACAAACCGGAGGAAGGTGGGGATGACGTCAAATCATCATG<br>CCCCTTATGACCTGGGCTACACACGTGCTACAATGGATGGTACAAAGGGCTGC<br>AAGACCGCGAGGTCAAGCCAATCCCATAAAACCATTCTCAGTTCGGATTGTAG<br>GCTGCAACTCGCCTACATGAAGCTGGAATCGCTAGTAATCGCGGATCAGCATG<br>CCGCGGTGAATACGTTCCCGGGCCTTGTACACACCGCCCGTCACACCACGAG<br>AGTTTGTAACACCCGAAGTCGGTGGAGTAACCGTAAGGAGCTAGCCGCCTAA<br>GGTGGGACAGATGATTGGGGGAAGTCGAACAAGTGCAT
Staphylococcus delphini	AGAGTTTGATCCTGGCTCAGTATGAACGCTGGCGGCGTGCCTAATACATGCAA<br>GTCGAGCGAACAGATAAGGAGCTTGCTCCTTTGACGTTAGCGGCGGACGGGT<br>GAGTAACACGTGGGTAACCTACCTATAAGACTGGAATAACTCCGGGAAACCGG<br>GGCTAATGCCGGATAACATGTTGAACCGCATGGTTCTACAGTGAAAGACGGTC<br>TTGCTGTCACTTATAGATGGACCCGCGCCGTATTAGCTAGTTGGTGGGGTAAC<br>GGCCTACCAAGGCGACGATACGTAGCCGACCTGAGAGGGTGATCGGCCACAC

	TGGAACTGAGACACGGTCCAGACTCCTACGGGAGGCAGCAGTAGGGAATCTT<br>CCGCAATGGGCGAAAGCCTGACGGAGCAACGCCGCGTGAGTGATGAAGGTCT<br>TCGGATCGTAAAGCTCTGTTGTTAGGGAAGAACAAATGTGTAAGTAACTGTGC<br>ACATCTTGACGGTACCTAACCAGAAAGCCACGGCTAACTACGTGCCAGCAGCC<br>GCGGTAATACGTAGGTGGCAAGCGTTATCCGGAATTATTGGGCGTAAAGCGC<br>GCGTAGGCGGTTTTTTAAGTCTGATGTGAAAGCCCACGGCTCAACCGTGGAG<br>GGTCATTGGAAACTGGAAAACTTGAGTGCAGAAGAGGAAAGTGGAATTCCATG<br>TGTAGCGGTGAAATGCGCAGAGATATGGAGGAACACCAGTGGCGAAGGCGGC<br>TTTCTGGTCTGCAACTGACGCTGATGTGCGAAAGCGTGGGGATCAAACAGGAT<br>TAGATACCCTGGTAGTCCACGCCGTAAACGATGAGTGCTAAGTGTTAGGGGGT<br>TTCCGCCCCTTAGTGCTGCAGCTAACGCATTAAGCACTCCGCCTGGGGAGTAC<br>GGTCGCAAGACTGAAACTCAAAGGAATTGACGGGGACCCGCACAAGCGGTGG<br>AGCATGTGGTTTAATTCGAAGCAACGCGAAGAACCTTACCAAATCTTGACATCC<br>TTTGACAACTCTAGAGATAGAGCTTTCCTCTTCGGAGGACAAAGTGACAGGTG<br>GTGCATGGTTGTCGTCAGCTCGTGTCGTGAGATGTTGGGTTAAGTCCCGCAAC<br>GAGCGCAACCCTTGAACTTAGTTGCCATCATTAAGTTGGGCACTCTAAGTTGA<br>CTGCCGGTGACAAACCGGAGGAAGGTGGGGATGACGTCAAATCATCATGCCC<br>CTTATGATTTGGGCTACACACGTGCTACAATGGACAATACAAAGGGCAGCGAA<br>ACCGCGAGGTCAAGCAAATCCCATAAAGTTGTTCTCAGTTCGGATTGTAGTCT<br>GCAACTCGACTACATGAAGCTGGAATCGCTAGTAATCGTAGATCAGCATGCTA<br>CGGTGAATACGTTCCCGGGTCTTGTACACACCGCCCGTCACACCACGAGAGT<br>TTGTAACACCCGAAGCCGGTGGAGTAACCATTTTGGAGCTAGCCGTCGAAGGT<br>GGGACAAATGATTGGGGT
Alkalihalobacillus gibsonii	GACGAACGCTGGCGACGTGCCTAATACATGCAAGTCGAGCGGACGTTTTTGA<br>AGCTTGCTCCANAAACGTTAGCGGCGGACGGGTGAGTAACACGTGGGCAACC<br>TACCTTATCGACTGGGATAACTCCGGGAAACCGGGGCTAATACCGGATAACAT<br>CTAGCACCTCCTGGTGCCGGATTAAAAGAGGGCTTCTTGCTCTCACGATGAGA<br>TGGGCCCGCGGCGCATTAGCTAGTTGGAGAGGTAACGGCTCCCCAAGNCGAC<br>GATGCGTAGCCGACCTGAGAGGGTGATCGGCCACACTGGGACTGAGACACG<br>GCCCAGACTCCTACGGGAGGCAGCAGTAGGGAATCTTCCGCAATGGACGAAA<br>GTCTGACGGAGCAACCCCGCGTGAGTGATGAAGGGTTTCGGCTCGTAAAGCT<br>CTGTTATGAGGGAAGAACACGTACCGTTCGAATAGGGCGGTACCTTGACGGTA<br>CCTCATCAGAAAGCCACGGCTAACTACGTGCCAGCAGCCGCGGTAATACGTA<br>GGTGGCAAGCGTTGTCCGGAATTATTGGGCGTAAAGCGCGCGCAGGCGGCCT<br>TTTAAGTCTGATGTGAAATCTTGCGGCTCAACCGCAAGCGGCCATTGGAAACT<br>GGGAGGCTTGAGTACAGAAGAGGAGAGTGGAATTCCACGTGTAGCGGTGAAA<br>TGCGTAGATATGTGGAGGAACACCAGTGGCGAAGGCGACTCTCTGGTCTGTA<br>ACTGACGCTGAGGCGCGAAAGCGTGGGGAGCAAACAGGATTAGATACCCTGG<br>TAGTCCACGCCGTAAACGATGAGTGCTAGGTGTTAGGGGTTTCGATGCCCGTA<br>GTGCCGAAGTTAACACATTAAGCACTCCGCCTGGGGAGTACGGCCGCAAGGC<br>TGAAACTCAAAGGAATTGACGGGGGCCCGCACAAGCAGTGGAGCATGTGGTT<br>TAATTCGAAGCAACGCGAAGAACCTTACCAGGTCTTGACATCCTTTGACCACT<br>CTGGAGACAGAGCTTCCCCTTCGGGGGCAAAGTGACAGGTGGTGCATGGTTG<br>TCGTCAGCTCGTGTCGTGAGATGTTGGGTTAAGTCCCGCAACGAGCGCAACC<br>CTTGACCTTAGTTGCCAGCATTTAGTTGGGCACTCTAAGGTGACTGCCGGTGA<br>CAAACCGGAGGAAGGTGGGGATGACGTCAAATCATCATGCCCCTTATGACCT<br>GGGCTACACACGTGCTACAATGGATGGTACAAAGGGTTGCGAAGCCGCGAGG<br>TGAAGCCAATCCCATAAAGCCATTCTCAGTTCGGATTGTAGGCTGCAACTCGC<br>CTGCATGAAGCTGGAATTGCTAGTAATCGCGGATCAGCATGCCGCGGTGAATA<br>CGTTCCCGGGCCTTGTACACACCGCCCGTCACACCACGAGAGTTTGTAACAC<br>CCGAAGTCGGTGAGGTAACCTTTTGGAGCCAGCCGCCGAAGGTGGGACAGAT<br>GATTGGGGTGAAGTCGTAACAAGGTAGCCGTATCGGAAGGTG
Rhodanobacter spathiphylli	ATTGAACGCTGGCGGCATGCCTAACACATGCAAGTCGAACGGCAGCACAGCA<br>GAGCTTGCTCTGTGGGTGGCGAGTGGCGGACGGGTGAGTAATGCATCGGGA<br>CCTACCCAGACGTGGGGGATAACGTAGGGAAACTTACGCTAATACCGCATAC<br>GTCCTACGGGAGAAAGCAGGGGACCTTCGGGCCTTGCGCGGTTGGACGGAC<br>CGATGTGCGATTAGCTAGTTGGTAGGGTAATGGCCTACCAAGGCGACGATCG<br>CTAGCTGGTCTGAGAGGATGATCAGCCACACTGGGACTGAGACACGGCCCAG<br>ACTCCTACGGGAGGCAGCAGTGGGGAATATTGGACAATGGGCGCAAGCCTGA<br>TCCAGCAATGCCGCGTGTGTGAAGAAGGCCCTCGGGTTGTAAAGCACTTTTAT<br>CAGGAACGAAATACCACGGGTTAATACCCTATGGGGCTGACGGTACCTGAGG<br>AATAAGCACCGGCTAACTTCGTGCCAGCAGCCGCGGTAATACGAAGGGTGCA<br>AGCGTTAATCGGAATTACTGGGCGTAAAGGGTGCGTAGGCGGTTCGTTAAGTC<br>TGTCGTGAAATCCCCGGGCTCAACCTGGGAATGGCGATGGATACTGGCGAGC<br>TAGAGTGTGTCAGAGGATGGTGGAATTCCCGGTGTAGCGGTGAAATGCGTAG

	AGATCGGGAGGAACATCAGTGGCGAAGGCGGCCATCTGGGACAACACTGACG<br>CTGAAGCACGAAAGCGTGGGGAGCAAACAGGATTAGATACCCTGGTAGTCCA<br>CGCCCTAAACGATGCGAACTGGATGTTGGTCTCAACTCGGAGATCAGTGTCGA<br>AGCTAACGCGTTAAGTTCGCCGCCTGGGGAGTACGGTCGCAAGACTGAAACT<br>CAAAGGAATTGACGGGGGCCCGCACAAGCGGTGGAGTATGTGGTTTAATTCG<br>ATGCAACGCGAAGAACCTTACCTGGCCTTGACATGTCCGGAATCCTTGAGAGA<br>TCGAGGAGTGCCTTCGGGAATCGGAACACAGGTGCTGCATGGCTGTCGTCAG<br>CTCGTGTCGTGAGATGTTGGGTTAAGTCCCGCAACGAGCGCAACCCTTGTCCT<br>TAGTTGCCAGCACGTAATGGTGGGAACTCTAAGGAGACTGCCGGTGACAAAC<br>CGGAGGAAGGTGGGGATGACGTCAAGTCATCATGGCCCTTACGGCCAGGGCT<br>ACACACGTACTACAATGGTCGGTACAGAGGGTTGCAATACCGCGAGGTGGAG<br>CCAATCCCAGAAAGCCGATCCCAGTCCGGATCGGAGTCTGCAACTCGACTCC<br>GTGAAGTCGGAATCGCTAGTAATCGCGGATCAGCTATGCCGCGGTGAATACG<br>TTCCCGGGCCTTGTACACACCGCCCGTCACACCATGGGAGTGGGTTGCTCCA<br>GAAGGCGTTAGTCTAACCGCAAGGGGGACGACGCCCACGGAGTGGTCCATGA<br>CTGGGGTGAAGTCGTAACAAGGTAGCCGTATCGGAAGG
Bacillus subtilis	CAGTGAATTCGAGGCGGCGTGCCTAATACATGCAAGTCGAGCGGACAGATGG<br>GAGCTTGCTCCCTGATGTTAGCGGCGGACGGGTGAGTAACACGTGGGTAACC<br>TGCCTGTAAGACTGGGATAACTCCGGGAAACCGGGGCTAATACCGGATGGTT<br>GTCTGAACCGCATGGTTCAGACATAAAAGGTGGCTTTGGCTACCACTTACAGA<br>TGGACCCGCGGCGCATTAGCTAGTTGGTGAGGTAACGGCTCACCAAGGCAAC<br>GATGCGTAGCCGACCTGAGAGGGTGATCGGCCACACTGGGACTGAGACACG<br>GCCCAGACTCCTACGGGAGGCAGCAGTAGGGAATCTTCCGCAATGGACGAAA<br>GTCTGACGGAGCAACGCCGCGTGAGTGATGAAGGTTTTCGGATCGTAAAGCT<br>CTGTTGTTAGGGAAGAACAAGTGCCGTTCAAATAGGGCGGCACCTTGACGGTA<br>CCTAACCAGAAAGCCACGGCTAACTACGTGCCAGCAGCCGCGGTAATACGTA<br>GGTGGCAAGCGTTGTCCGGAATTATTGGGCGTAAAGGGCTCGCAGGCGGTTT<br>CTTAAGTCTGATGTGAAAGCCCCCGGCTCAACCGGGGAGGGTCATTGGAAAC<br>TGGGGAACTTGAGTGCAGAAGAGGAGAGTGGAATTCCACGTGTAGCGGTGAA<br>ATGCGTAGAGATGTGGAGGAACACCAGTGGCGAAGGCGACTCTCTGGTCTGT<br>AACTGACGCTGAGGAGCGAAAGCGTGGGGAGCGAACAGGATTAGATACCCTG<br>GTAGTCCACGCCGTAAACGATGAGTGCTAAGTGTTAGGGGGTTTCCGCCCCTT<br>AGTGCTGCAGCTAACGCATTAAGCACTCCGCCTGGGGAGTACGGTCGCAAGA<br>CTGAAACTCAAAGGAATTGACGGGGGCCCGCACAAGCGGTGGAGCATGTGGT<br>TTAATTCGAAGCAACGCGAAGAACCTTACCAGGTCTTGACATCCTCTGACAATC<br>CTAGAGATAGGACGTCCCCTTCGGGGGCAGAGTGACAGGTGGTGCATGGTTG<br>TCGTCAGCTCGTGTCGTGAGATGTTGGGTTAAGTCCCGCAACGAGCGCAACC<br>CTTGATCTTAGTTGCCAGCATTCAGTTGGGCACTCTAAGGTGACTGCCGGTGA<br>CAAACCGGAGGAAGGTGGGGATGACGTCAAATCATCATGCCCCTTATGACCT<br>GGGCTACACACGTGCTACAATGGACAGAACAAAGGGCAGCGAAACCGCGAGG<br>TTAAGCCAATCCCACAAATCTGTTCTCAGTTCGGATCGCAGTCTGCAACTCGA<br>CTGCGTGAAGCTGGAATCGCTAGTAATCGCGGATCAGCATGCCGCGGTGAAT<br>ACGTTCCCGGGCCTTGTACACACCGCCCGTCACACCACGAGAGTTTGTAACAC<br>CCGAAGTCGGTGAGGTAACCTTTATGGAGCCAGCCGCCGAAGGTGGGACAGA<br>TGATT
Iodobacter fluviatile	ATTGAACGCTGGCGGCATGCTTTACACATGCAAGTCGAACGGTAACAGGGTG<br>CTTGCACCGCTGACGAGTGGCGAACGGGTGAGTAATATATCGGAACGTACCT<br>AGTAATGGGGGATAACTATCCGAAAGGATAGCTAATACCGCATACGCCCTGAG<br>GGGGAAAGAGGGGGATCGCAAGACCTCTCGTTATTAGAGCGGCCGATATCAG<br>ATTAGCTAGTTGGTGAGGTAAAGGCTCACCAAGGCCACGATCTGTAGCGGGT<br>CTTAGAGGACGATCCGCCACACTGGAACTGAGACACGGTCCAGACTCCTACG<br>GGAGGCAGCAGTGGGGAATCTTGGACAATGGGCGCAAGCCTGATCCAGCAAT<br>GCCGCGTGCGTGAAGAAGGCCTTCGGGTTGTAAAGCGCTTTTGTTCGGGAGG<br>AAATCCTAGTGGCTAATATCCATTGGGGATGACAGTACCGGAAGAATAAGGAC<br>CGGCTAACTACGTGCCAGCAGCCGCGGTAATACGTAGGGTCCAAGCGTTAAT<br>CGGAATTACTGGGCGTAAAGGGTGCGCAGGTGGTTNATTAAGTGTGATGTGAA<br>AGCCCCGGGCTCAACCTGGGAATTGCATTGCAAACTGGTCAACTAGAGTATGG<br>CAGAGGGGGGTGGAATTCCGCGTGTAGCAGTGAAATGCGTAGAGATGCGGAG<br>GAACACCGATGGCGAAGGCAACCCCCTGGGCTAATACTGACACTCATGCACG<br>AAAGCGTGGGGAGCAAACAGGATTAGATACCCTGGTAGTCCACGCCCTAAAC<br>GATGTCTACTAGTTGTTGGGGAATTCGTTCCTTAGTAACGCAGCTAACGCGTG<br>AAGTAGACCGCCTGGGGAGTACGGCCGCAAGGCTAAAACTCAAAGGAATTGA<br>CGGGGGCCCGCACAAGCGGTGGATGATGTGGATTAATTCGATGCAACGCGAA<br>AAACCTTACCTAGCCTTGACATGNNNNGAATCCCTGAGAGATTGGGGAGTGCC<br>GCAAGGAACNNNNACACAGGTGCTGCATGGCTGTCGTCAGCTCGTGTCGTGA

	GATGTTGGGTTAAGTCCCGCAACGAGCGCAACCCTTGTCCTTAGTTGCTACCA<br>TTTAGTTGGGCACTTTAAGGAGACTGCCGGTGACAAACCGGAGGAAGGTGGG<br>GATGACGTCAAGTCCTCATGGCCCTTATGGCTAGGGCTTCACACGTCATACAA<br>TGGTCGGTACAGAGGGTTGCCAAGCCGCGAGGTGGAGCTAATCTCATAAAAC<br>CGATCGTAGTCCGGATTGGAGTCTGCAACTCGACTCCATGAAGTCGGAATCGC<br>TAGTAATCGCGGATCAGCATGTCGCGGTGAATACGTTCCCGGGCCTTGTACAC<br>ACCGCCCGTCACACCATGGGAATGGGTTTCACCAGAAGTAGGTAGGCTAACC<br>GTAAGGAGGCCGCTTACCACGGTGGGATTCATGACTGGGGTGAAG
Sphingomonas roseiflava	ATGGCTCAGAACGAACGNTGGAGGCATGCCTAACACATGCAAGTCGAACGAG<br>ATCCTTCGGGGTTTAGTGGCGCACGGGTGCGTAACGCGTGGGAATCTGCCCT<br>TTGGTTCGGAATAACAGTTGGAAACGGCTGCTAATACCGGATGATGACGAAAG<br>TCCAAAGATTTATCGCCAGAGGATGAGCCCGCGTTGGATTAGGTAGTTGGTGG<br>GGTAAAGGCNTACCAAGCCGACGATCCATAGCTGGTCTGAGAGGATGATCAG<br>CCACACTGGGACTGAGACACGGCCCAGACTCCTACGGGAGGCAGCAGTGGG<br>GAATATTGGACAATGGGCGAAAGCCTGATCCAGCAATGCCGCGTGAGTGATG<br>AAGGCCCTAGGGTTGTAAAGCTCTTTTACCCGGGAAGATAATGACTGTACCGG<br>GAGAATAAGCCCCGGCTAACTCCGTGCCAGCAGCCGCGGTAATACGGAGGG<br>GGCTAGCGTTGTTCGGAATTACTGGGCGTAAAGCGCACGTAGGCGGCTTTGT<br>AAGTTAGAGGTGAAAGCCTGGAGCTCAACTCCAGAACTGCCTTTGATACTGCA<br>TGGCTTGAATCCTGGAGAGGTNAGTGGAATTCCGAGTGTAGAGGTGAAATTCG<br>TAGATATTCGGAAGAACACCAGTTGCGAAGGCGGCTNACTGGACTGGTATTGA<br>CGCTGAGGTGCGAAACCGTGGGGAGCAAACAGGATTAGATACCCTGGTAGTC<br>CACGCCGTAAACGATGATAACTAGCTGTCCGGGGACTTGGTCTTTGGGTGGC<br>GCACGTAACGCATTAAGTTATCCGCCTGGGGAGTACGGCCGCAAGGTTAAAA<br>CTCAAAGTAATTGACGGGNNCCTGCACAAGCGGTGGAGCATGTGGTTTAATTC<br>GAAGCAACGCGCAGAACCTTACCAGCGTTTGACATGTCCGGACGACTGGCAG<br>AGATGCCTTTCTTCCTAACGGGGACTGGAACACAGGTGCTGCATGGCTGTCGT<br>CAGCTCGTGTCGTGAGATGTTGGGTTAAGTCCCGCAACGAGCGCAACCCTCG<br>CCTTTAGTTACCATCATTTGGTTGGGTACTCTAAAGGAACCGCCGGTGATAAG<br>CCGGAGGAAGGTGGGGATGACGTCAAGTCCTCATGGCCCTTACGCGCTGGG<br>CTACACACGTGCTACAATGGCAACTACAGTGGGCAGCGACCCTGCGAGGGCG<br>AGCTAATCCCCAAAAGTTGTCTCAGTTCGGATTGTTCTCTGCAACTCGAGAGC<br>ATGAAGGCGGAATCGCTAGTAATCGCGGATCAGCATGCCGCGGTGAATACGT<br>TCCCAGGCCTTGTACACACCGCCCGTCACACCATGGGAGTTGGATTCACCCG<br>AAGGCGTTGCGCCAACCTAGCAATAGGAAGCAGGCGACCACGGTGGGTTCAG<br>CGAC
Paenarthrobacter aurescens	TTTGATCCTGGCTCAGGATGAACGCTGGCGGCGTGCTTAACACATGCAAGTCG<br>AACGATGATCCCAGCTTGCTGGGGGATTAGTGGCGAACGGGTGAGTAACACG<br>TGAGTAACCTGCCCTTGACTCTGGGATAAGCCTGGGAAACTGGGTCTAATACC<br>GGATATGACCATCTGGCGCATGTCATGGTGGTGGAAAGCTTTTTGTGGTTTTG<br>GATGGACTCGCGGCCTATCAGCTTGTTGGTGGGGTAATGGCCTACCAAGGCG<br>ACGACGGGTAGCCGGCCTGAGAGGGTGACCGGCCACACTGGGACTGAGACA<br>CGGCCCAGACTCCTACGGGAGGCAGCAGTGGGGAATATTGCACAATGGGCG<br>CAAGCCTGATGCAGCGACGCCGCGTGAGGGATGACGGCCTTCGGGTTGTAAA<br>CCTCTTTCAGTAGGGAAGAAGCGAAAGTGACGGTACCTGCAGAAGAAGCGCC<br>GGCTAACTACGTGCCAGCAGCCGCGGTAATACGTAGGGCGCAAGCGTTATCC<br>GGAATTATTGGGCGTAAAGAGCTCGTAGGCGGTTTGTCGCGTCTGCTGTGAAA<br>GACCGGGGCTCAACTCCGGTTCTGCAGTGGGTACGGGCAGACTAGAGTGCAG<br>TAGGGGAGACTGGAATTCCTGGTGTAGCGGTGAAATGCGCAGATATCAGGAG<br>GAACACCGATGGCGAAGGCAGGTCTCTGGGCTGTAACTGACGCTGAGGAGCG<br>AAAGCATGGGGAGCGAACAGGATTAGATACCCTGGTAGTCCATGCCGTAAAC<br>GTTGGGCACTAGGTGTGGGGGACATTCCACGTTTTCCGCGCCGTAGCTAACG<br>CATTAAGTGCCCCGCCTGGGGAGTACGGCCGCAAGGCTAAAACTCAAAGGAA<br>TTGACGGGGGCCCGCACAAGCGGCGGAGCATGCGGATTAATTCGATGCAACG<br>CGAAGAACCTTACCAAGGCTTGACATGAACCGGAAAGACCTGGAAACAGGTG<br>CCCCGCTTGCGGTCGGTTTACAGGTGGTGCATGGTTGTCGTCAGCTCGTGTC<br>GTGAGATGTTGGGTTAAGTCCCGCAACGAGCGCAACCCTCGTTCTATGTTGCC<br>AGCGCGTTATGGCGGGGACTCATAGGAGACTGCCGGGGTCAACTCGGAGGA<br>AGGTGGGGACGACGTCAAATCATCATGCCCCTTATGTCTTGGGCTTCACGCAT<br>GCTACAATGGCCGGTACAAAGGGTTGCGATACTGTGAGGTGGAGCTAATCCC<br>AAAAAGCCGGTCTCAGTTCGGATTGGGGTCTGCAACTCGACCCCATGAAGTC<br>GGAGTCGCTAGTAATCGCAGATCAGCAACGCTGCGGTGAATACGTTCCCGGG<br>CCTTGTACACACCGCCCGTCAAGTCACGAAAGTTGGTAACACCCGAAGCCGG<br>TGGCCTAACCCTTGTGGGGGGAGCCGTCGAAGGTGGGACCGGCGATTGGGA

	CTAAGTCGTAACAAGGTAGCCGTACCGGAAGGTGCGGCTGGATCACCTCCTTT<br>TCTA
Phyllobacterium myrsinacearum	AACGAACGCTGGCGGCAGGCTTAACACATGCAAGTCGAGCGCCCCGCAAGG<br>GGAGCGGCAGACGGGTGAGTAACGCGTGGGAATCTACCCATCTCTACGGAAT<br>AACGCATGGAAACGTGTGCTAATACCGTATACGTCCTTCGGGAGAAAGATTTA<br>TCGGAGATGGATGAGCCCGCGTTGGATTAGCTAGTTGGTGGGGTAAAGGCCT<br>ACCAAGGCGACGATCCATAGCTGGTCTGAGAGGATGATCAGCCACACTGGGA<br>CTGAGACACGGCCCAGACTCCTACGGGAGGCAGCAGTGGGGAATATTGGACA<br>ATGGGCGCAAGCCTGATCCAGCCATGCCGCGTGAGTGATGAAGGCCCTAGGG<br>TTGTAAAGCTCTTTCACCGGTGAAGATAATGACGGTAACCGGAGAAGAAGCCC<br>CGGCTAACTTCGTGCCAGCAGCCGCGGTAATACGAAGGGGGCTAGCGTTGTT<br>CGGATTTACTGGGCGTAAAGCGCACGTAGGCGGACTATTAAGTCAGGGGTGA<br>AATCCCGGGGCTCAACCCCGGAACTGCCTTTGATACTGGTAGTCTTGAGTTCG<br>AGAGAGGTGAGTGGAATTCCGAGTGTAGAGGTGAAATTCGTAGATATTCGGAG<br>GAACACCAGTGGCGAAGGCGGCTCACTGGCTCGATACTGACGCTGAGGTGCG<br>AAAGCGTGGGGAGCAAACAGGATTAGATACCCTGGTAGTCCACGCCGTAAAC<br>TATGAGAGCTAGCCGTCGGGCAGTATACTGTTCGGTGGCGCAGCAAACGCAT<br>TAAGCTCTCCGCCTGGGGAGTACGGTCGCAAGATTAAAACTCAAAGGAATTGA<br>CGGGGGCCCGCACAAGCGGTGGAGCATGTGGTTTAATTCGAAGCAACGCGCA<br>GAACCTTACCAGCCCTTGACATCCCGATCGCGGTTACCAGAGATGGTTTCCTT<br>CAGTTAGGCTGGATCGGTGACAGGTGCTGCATGGCTGTCGTCAGCTCGTGTC<br>GTGAGATGTTGGGTTAAGTCCCGCAACGAGCGCAACCCTCGCCCTTAGTTGC<br>CATCATTCAGTTGGGCACTCTAAGGGGACTGCCGGTGATAAGCCGAGAGGAA<br>GGTGGGGATGACGTCAAGTCCTCATGGCCCTTACGGGCTGGGCTACACACGT<br>GCTACAATGGTGGTGACAGTGGGCAGCGAGACCGCGAGGTCGAGCTAATCTC<br>CAAAAGCCATCTCAGTTCGGATTGCACTCTGCAACTCGAGTGCATGAAGTTGG<br>AATCGCTAGTAATCGTGGATCAGAATGCCACGGTGAATACGTTCCCGGGCCTT<br>GTACACACCGCCCGTCACACCATGGGAGTTGGTTTTACCCGAAGGTGCTGTG<br>CTAACCGCAAGGAGGCAGGCAACCACGGTAGGGTCAGCGACTGGGGTGAAG
Cellulosimicrobium cellulans	CTCAGGACGAACGCTGGCGGCGTGCTTAACACATGCAAGTCGAACGATGATG<br>CCCAGCTTGCTGGGTGGATTAGTGGCGAACGGGTGAGTAACACGTGAGTAAC<br>CTGCCCTTGACTTCGGGATAACTCCGGGAAACCGGGGCTAATACCGGATATG<br>AGCTACCTTCGCATGGGGGTGGTTGGAAAGTTTTTCGGTCAAGGATGGGCTC<br>GCGGCCTATCAGCTTGTTGGTGGGGTGATGGCCTACCAAGGCGACGACGGGT<br>AGCCGGCCTGAGAGGGCGACCGGCCACACTGGGACTGAGACACGGCCCAGA<br>CTCCTACGGGAGGCAGCAGTGGGGAATATTGCACAATGGGCGAAAGCCTGAT<br>GCAGCGACGCCGCGTGAGGGATGAAGGCCTTCGGGTTGTAAACCTCTTTCAG<br>CAGGGAAGAAGCGCAAGTGACGGTACCTGCAGAAGAAGCGCCGGCTAACTAC<br>GTGCCAGCAGCCGCGGTAATACGTAGGGCGCAAGCGTTGTCCGGAATTATTG<br>GGCGTAAAGAGCTCGTAGGCGGTTTGTCGCGTCTGGTGTGAAAACTCGAGGC<br>TCAACCTCGAGCTTGCATCGGGTACGGGCAGACTAGAGTGCGGTAGGGGAGA<br>CTGGAATTCCTGGTGTAGCGGTGGAATGCGCAGATATCAGGAGGAACACCGA<br>TGGCGAAGGCAGGTCTCTGGGCCGCAACTGACGCTGAGGAGCGAAAGCATG<br>GGGAGCGAACAGGATTAGATACCCTGGTAGTCCATGCCGTAAACGTTGGGCA<br>CTAGGTGTGGGGCTCATTCCACGAGTTCCGTGCCGCAGCAAACGCATTAAGT<br>GCCCCGCCTGGGGAGTACGGCCGCAAGGCTAAAACTCAAAGGAATTGACGGG<br>GGCCCGCACAAGCGGCGGAGCATGCGGATTAATTCGATGCAACGCGAAGAAC<br>CTTACCAAGGCTTGACATGCACGGGAAGCCACCAGAGATGGTGGTCTCTTTG<br>GACACTCGTGCACAGGTGGTGCATGGTTGTCGTCAGCTCGTGTCGTGAGATG<br>TTGGGTTAAGTCCCGCAACGAGCGCAACCCTCGTCCCATGTTGCCAGCGGGT<br>TATGCCGGGGACTCATGGGAGACTGCCGGGGTCAACTCGGAGGAAGGTGGG<br>GATGACGTCAAATCATCATGCCCCTTATGTCTTGGGCTTCACGCATGCTACAAT<br>GGCCGGTACAAAGGGCTGCGATACCGTAAGGTGGAGCGAATCCCAAAAAGCC<br>GGTCTCAGTTCGGATTGGGGTCTGCAACTCGACCCCATGAAGTCGGAGTCGC<br>TAGTAATCGCAGATCAGCAACGCTGCGGTGAATACGTTCCCGGGCCTTGTACA<br>CACCGCCCGTCAAGTCACGAAAGTCGGTAACACCCGAAGCCCATGGCCCAAC<br>CGTTCGCGGGGGGAGTGGTCGAAGGTGGGACTGGCGATTGGGACTAAGTCG<br>TAACAAGGTAGCCGTACCGGAA
Marinobacter nauticus	CAGCTCAGATTGAACGCTGGCGGCAGGCTTAACACATGCAAGTCGAGCGGTA<br>ACAGGGGTAGCTTGNTACCCGCTGACGAGCGGCGGACGGGTGAGTAATGCTT<br>AGGAATCTGCCCAGTAGTGGGGGGATAGCCCGGGGAAACCCGGATTAATACC<br>GCATACGTCCTACGGGAGAAAGCAGGGGATCTTCGGACCTTGCGCTATTGGA<br>TGAGCCTAAGTCGGATTAGCTAGTTGGTGGGGTAAAGGCCTACCAAGGCGAC

	GATCCGTAGCTGGTCTGAGAGGATGATCAGCCACATCGGGACTGAGACACGG<br>CCCGAACTCCTACGGGAGGCAGCAGTGGGGAATATTGGACAATGGGGGCAAC<br>CCTGATCCAGCCATGCCGCGTGTGTGAAGAAGGCTTTCGGGTTGTAAAGCACT<br>TTCAGTAGGGAGGAAAACCTTATGGCTAATACCCATGAGGCTTGACGTTACCT<br>ACAGAAGAAGCACCGGCTAACTCCGTGCCAGCAGCCGCGGTAATACGGAGGG<br>TGCAAGCGTTAATCGGAATTACTGGGCGTAAAGCGCGCGTAGGTGGTTTGGTA<br>AGCGAGATGTGAAAGCCCCGGGCTTAACCTGGGAACGGCATTTCGAACTGTC<br>AGACTAGAGTGTGGTAGAGGGTAGTGGAATTTCCTGTGTAGCGGTGAAATGC<br>GTAGATATAGGAAGGAACACCAGTGGCGAAGGCGGCTACCTGGACCAACACT<br>GACACTGAGGTGCGAAAGCGTGGGGAGCAAACAGGATTAGATACCCTGGTAG<br>TCCACGCCGTAAACGATGTCAACTAGCCGTTGGGACTCTTGAAGTCTTAGTGG<br>CGCAGCTAACGCACTAAGTTGACCGCCTGGGGAGTACGGCCGCAAGGTTAAA<br>ACTCAAATGAATTGACGGGGGCCCGCACAAGCGGTGGAGCATGTGGTTTAATT<br>CGACGCAACGCGAAGAACCTTACCTGGCCTTGACATCCAGAGAACTTTCCAGA<br>GATGGATTGGTGCCTTCGGGAACTCTGAGACAGGTGCTGCATGGCCGTCGTC<br>AGCTCGTGTCGTGAGATGTTGGGTTAAGTCCCGTAACGAGCGCAACCCCTATC<br>CCTGGTTGCTAGCAGGTAATGCTGAGAACTCCAGGGAGACTGCCGGTGACAA<br>ACCGGAGGAAGGTGGGGATGACGTCAGGTCATCATGGCCCTTACGGCCAGG<br>GCTACACACGTGCTACAATGGCGCGTACAGAGGGCTGCCAACTCGCGAGAGT<br>GAGCCAATCCCTTAAAACGCGTCGTAGTCCGGATCGGAGTCTGCAACTCGACT<br>CCGTGAAGTCGGAATCGCTAGTAATCGCGAATCAGAATGTCGCGGTGAATACG<br>TTCCCGGGCCTTGTACACACCGCCCGTCACACCATGGGAGTGGATTGCACCA<br>GAAGTAGTTAGTCTAAC
Kurthia sibirica	TGATCCTGGCTCAGGACGAACGCTGGCGGCGTGCCTAATACATGCAAGTCGA<br>GCGAATGATGAAGAAGCTTGCTTCTTCTGATTTAGCGGCGGACGGGTGAGTAA<br>CACGTGGGCAACCTGCCCTACAGATTGGGATAACTCCGGGAAACCGGGGCTA<br>ATACCGAATAATCCATTTTGCCTCATGGCGAAATGTTGAAAGGCGCTTCGGCG<br>TCACTGTAGGATGGGCCCGCGCTGCATTAGCTAGTTGGTGAGGTAACGGCTC<br>ACCAAGGCCACGATGCATAGCCGACCTGAGAGGGTGATCGGCCACATTGGGA<br>CTGAGACACGGCCCAAACTCCTACGGGAGGCAGCAGTAGGGAATCTTCCACA<br>ATGGACGAAAGTCTGATGGAGCAACGCCGCGTGAGTGATGAAGGTTTTCGGA<br>TCGTAAAACTCTGTTGTAAGGGAAGAACAAGTGCGTTAGGTAATGAACGCACC<br>ATGACGGTACCTTATTAGAAAGCCACGGCTAACTACGTGCCAGCAGCCGCGG<br>TAATACGTAGGTGGCAAGCGTTGTCCGGATTTATTGGGCGTAAAGCGCGCGC<br>AGGTGGTTTCTTAAGTCTGATGTGAAAGCCCCCGGCTCAACCGGGGAGGGTC<br>ATTGGAAACTGGGAAACTTGAGTACAGAAGAGGATAGTGGAATTCCAAGTGTA<br>GCGGTGAAATGCGTAGAGATTTGGAGGAACACCAGTGGCGAAGGCGACTGTC<br>TGGTCTGTAACTGACACTGAGGCGCGAAAGCGTGGGGAGCAAACAGGATTAG<br>ATACCCTGGTAGTCCACGCCGTAAACGATGAGTGCTAAGTGTTAGGGGGTTTC<br>CGCCCCTTAGTGCTGCAGCTAACGCATTAAGCACTCCGCCTGGGGAGTACGA<br>CCGCAAGGTTGAAACTCAAAGGAATTGACGGGGGCCCGCACAAGCGGTGGAG<br>CATGTGGTTTAATTCGAAGCAACGCGAAGAACCTTACCAGGTCTTGACATCCC<br>AATGACCGTCCTAGAGATAGGATTTTCCCTTCGGGGACATTGGTGACAGGTGG<br>TGCATGGTTGTCGTCAGCTCGTGTCGTGAGATGTTGGGTTAAGTCCCGCAACG<br>AGCGCAACCCTTAATGTTAGTTGCCATCATTTAGTTGGGCACTCTAATGTGACT<br>GCCGGTGATAAACCGGAGGAAGGTGGGGATGACGTCAAATCATCATGCCCCT<br>TATGACCTGGGCTACACACGTGCTACAATGGGCGATACAAAGAGTCGCAAACT<br>CGCGAGGGTAAGCTAATCTCATAAAATCGTTCTCAGTTCGGATTGTAGGCTGC<br>AACTCGCCTACATGAAGCCGGAATCGCTAGTAATCGCGGATCAGCATGCCGC<br>GGTGAATACGTTCCCGGGCCTTGTACACACCGCCCGTCACACCACGAGAGTT<br>TGTAACACCCGAAGTCGGTGGGGTAACCATTTATGGAGCCAGCCGCCTAAGG<br>TGGGATAGATGATTGGGGTGAAGTCGTAACAAGGTAGCCGTATCGGAAGGTG<br>CGGCTGGATCACCTCC
Agrococcus versicolor	GATGAACGCTGGCGGCGTGCTTAACACATGCAAGTCGAACGGTGAAGGAGGA<br>GCTTGCTCCTCCGGATCAGTGGCGAACGGGTGAGTAACACGTGAGCAATCTG<br>CCCCTGACTCTGGGATAAGCGTTGGAAACGACGTCTAATACCGGATACGAGCT<br>GAGAAGGCATCTTCAGCAGCTGGAAAGAACTTCGGTCAGGGATGAGCTCGCG<br>GCCTATCAGGTAGTTGGTGAGGTAACGGCTCACCAAGCCTACGACGGGTAGC<br>CGGCCTGAGAGGGTGACCGGCCACACTGGGACTGAGACACGGCCCAGACTC<br>CTACGGGAGGCAGCAGTGGGGAATATTGCACAATGGGCGAAAGCCTGATGCA<br>GCAACGCCGCGTGAGGGACGAAGGCCTTCGGGTTGTAAACCTCTTTTAGCAG<br>GGAAGAAGCGAAAGTGACGGTACCTGCAGAAAAAGCACCGGCTAACTACGTG<br>CCAGCAGCCGCGGTAATACGTAGGGTGCGAGCGTTATCCGGAATTATTGGGC<br>GTAAAGAGCTCGTAGGCGGTCTGTCGCGTCTGCTGTGAAAACCCGAGGCTCA<br>ACCTCGGGCTTGCAGTGGGTACGGGCAGACTAGAGTGTGGTAGGGGAGATTG

	GAATTCCTGGTGTAGCGGTGGAATGCGCAGATATCAGGAGGAACACCGATGG<br>CGAAGGCAGATCTCTGGGCCATTACTGACGCTGAGGAGCGAAAGCGTGGGGA<br>GCAAACAGGCTTAGATACCCTGGTAGTCCACGCCGTAAACGTTGGGAACTAGA<br>TGTGGGGACCATTCCACGGTCTCCGTGTCGTAGCTAACGCATTAAGTTCCCCG<br>CCTGGGGAGTACGGCCGCAAGGCTAAAACTCAAAGGAATTGACGGGGGCCC<br>GCACAAGCGGCGGAGCATGCGGATTAATTCGATGCAACGCGAAGAACCTTAC<br>CAAGGCTTGACATATACGAGAACGCACTGGAGACAGTGAACTCTTTGGACACT<br>CGTAAACAGGTGGTGCATGGTTGTCGTCAGCTCGTGTCGTGAGATGTTGGGTT<br>AAGTCCCGCAACGAGCGCAACCCTCGTCCTATGTTGCCAGCACGTAATGGTG<br>GGAACTCATGGGATACTGCCGGGGTCAACTCGGAGGAAGGTGGGGATGAGG<br>TCAAATCATCATGCCCCTTATGTCTTGGGCTTCACGCATGCTACAATGGCCGG<br>TACAAAGGGCTGCAATACCGTAAGGTGGAGCGAATCCCAAAAAGCCGGTCTC<br>AGTTCGGATTGAGGTCTGCAACTCGACCTCATGAAGTCGGAGTCGCTAGTAAT<br>CGCAGATCAGCAACGCTGCGGTGAATACGTTCCCGGGCCTTGTACACACCGC<br>CCGTCAAGTCATGAAAGTCGGTAACACCCGAAGCCGGTGGCCTAACCCTTGT<br>GGAGGGAGCCGTCGAAGGTGGGATCGGTAATTAGGACTAAGTCGTAACAAGG<br>TAGCCGTACCGGAAGGTGC
Kitasatospora herbaricolor	ACGAACGCTGGCGGCGTGCTTAACACATGCAAGTCGAACGGTGAAGCCCTTC<br>GGGGTGGATCAGTGGCGAACGGGTGAGTAACACGTGGGCAATCTGCCCTGCA<br>CTCTGGGACAAGCCCTGGAAACGGGGTCTAATACCGGATATGACCTTCCTCC<br>GCATGGGGGTTGGTGTAAAGCTCCGGCGGTGCAGGATGAGCCCGCGGCCTA<br>TCAGCTTGTTGGTGGGGTAATGGCCTACCAAGGCGACGACGGGTAGCCGGCC<br>TGAGAGGGCGACCGGCCACACTGGGACTGAGACACGGCCCAGACTCCTACG<br>GGAGGCAGCAGTGGGGAATATTGCACAATGGGCGAAAGCCTGATGCAGCGAC<br>GCCGCGTGAGGGATGACGGCCTTCGGGTTGTAAACCTCTTTCAGCAGGGAAG<br>AAGCGCAAGTGACGGTACCTGCAGAAGAAGCACCGGCTAACTACGTGCCAGC<br>AGCCGCGGTAATACGTAGGGTGCGAGCGTTGTCCGGAATTATTGGGCGTAAA<br>GAGCTCGTAGGCGGCCTGTCGCGTCGGATGTGAAAGCCCGGGGCTTAACCC<br>CGGGTCTGCATTCGATACGGGCAGGCTAGAGTGTGGTAGGGGAGATCGGAAT<br>TCCTGGTGTAGCGGTGAAATGCGCAGATATCAGGAGGAACACCGGTGGCGAA<br>GGCGGATCTCTGGGCCATTACTGACGCTGAGGAGCGAAAGCGTGGGGAGCG<br>AACAGGATTAGATACCCTGGTAGTCCACGCCGTAAACGTTGGGAACTAGGTGT<br>TGGCGACATTCCACGTCGTCGGTGCCGCAGCTAACGCATTAAGTTCCCCGCC<br>TGGGGAGTACGGCCGCAAGGCTAAAACTCAAAGGAATTGACGGGGGCCCGCA<br>CAAGCAGCGGAGCATGTGGCTTAATTCGACGCAACGCGAAGAACCTTACCAA<br>GGCTTGACATATACCGGAAACGGCCAGAGATGGTCGCCCCCTTGTGGTCGGT<br>ATACAGGTGGTGCATGGTTGTCGTCAGCTCGTGTCGTGAGATGTTGGGTTAAG<br>TCCCGCAACGAGCGCAACCCTTGTTCTGTGTTGCCAGCATGCCTTTCGGGGT<br>GATGGGGACTCACAGGAGACTGCCGGGGTCAACTCGGAGGAAGGTGGGGAC<br>GACGTCAAATCATCATGCCCCTTATGTCTTGGGCTGCACACGTGCTACAATGG<br>TCGGTACAAAGGGCTGCGATGCCGCGAGGCGGAGCGAATCCCAAAAAGCCG<br>GCCTCAGTTCGGATTGGGGTCTGCAACTCGACCCCATGAAGTTGGAGTTGCTA<br>GTAATCGCAGATCAGCATGCTGCGGTGAATACGTTCCCGGGCCTTGTACACAC<br>CGCCCGTCACGTCACGAAAGTCGGTAACACCCGAAGCCGGTGGCCTAACCCG<br>TAAGGGGAGGAGCCGTCGAAGGTGGGACCAGCGATTGGGACGAAGTCGTAA<br>CAAGGTAGCCGTAC
Photorhabdus luminescens	CCTGGCTCAGATTGAACGCTGGCGGCAGGCCTAACACATGCAAGTCGAGCGG<br>TAACAGGAAAGCGCTTGCGCTTTTGCTGACGAGCGGCGGACGGGTGAGTAAT<br>GTCTGGGGATCTCCCCGAGGGCGGGGGATAACCACTGGAAACGGTGGCTAAT<br>ACCGCATAATGTCGCGAGACCAAAGTGGGGGACCTGAAAGGGCCTCACGCCG<br>TCGGATGAACCCAGATGGGATTAGCTAGTAGGTAGGGTAAAGGCCTACCTAG<br>GCGACGATCCCTAGCTGGTCTGAGAGGATGACCAGCCACACTGGGACTGAGA<br>CACGGCCCAGACTCCTACGGGAGGCAGCAGTGGGGAATATTGCACAATGGGC<br>GCAAGCCTGATGCAGCCATGCCGCGTGTATGAAGAAGGCCTTCGGGTTGTAA<br>AGTACTTTCAGCGGGGAGGAAGGGTTCAGCTTGAACAGAGCTGAATTTTGACG<br>TTACCCGCAGAAGAAGCACCGGCTAACTCCGTGCCAGCAGCCGCGGTAATAC<br>GGAGGGTGCAAGCGTTAATCGGAATGACTGGGCGTAAAGCGCACGCAGGCG<br>GTCAATTAAGTTAGATGTGAAATCCCCGGGCTCAACCTGGGAATGGCATCTAA<br>GACTGGTTGGCTGGAGTCTCGTAGAGGGGGGTAGAATTCCATGTGTAGCGGT<br>GAAATGCGTAGAGATGTGGAGGAATACCGGTGGCGAAGGCGGCCCCCTGGA<br>CGAAGACTGACGCTCAGGTGCGAAAGCGTGGGGAGCAAACAGGATTAGATAC<br>CCTGGTAGTCCACGCTGTAAACGATGTCGATTTGGAGGTTGTGGCCTTGAGCT<br>GTGGCTTCCGAAGCTAACGCGTTAAATCGACCGCCTGGGGAGTACGGCCGCA<br>AGGTTAAAACTCAAATGAATTGACGGGGGCCCGCACAAGCGGTGGAGCATGT<br>GGTTTAATTCGATGCAACGCGAAGAACCTTACCTACTCTTGACATCCAGAGAA

	GACCTCAGAGATGAGGTTGTGCCTTCGGGAGCTCTGAGACAGGTGCTGCATG<br>GCTGTCGTCAGCTCGTGTTGTGAAATGTTGGGTTAAGTCCCGCAACGAGCGCA<br>ACCCTTATCCTTTGTTGCCAGCGCGTAATGGCGGGAACTCAAAGGAGACTGCC<br>GGTGATAAACCGGAGGAAGGTGGGGATGACGTCAAGTCATCATGGCCCTGAC<br>GAGTAGGGCTACACACGTGCTACAATGGCGGATACAAAGTGAAGCGACCTCG<br>CGAGAGCAAGCGGAACACACAAAGTCTGTCGTAGTCCGGATTGGAGTCTGCA<br>ACTCGACTCCATGAAGTCGGAATCGCTAGTAATCGTAGATCAGCATGCTACGG<br>TGAATACGTTCCCGGGCCTTGTACACACCGCCCGTCACACCATGGGAGTGGG<br>TTGCAAAAGAAGTCGGTAGCTTAACCGCAAGGAGGGCGCTGACCACTTTGTG<br>GCTCATGACTGGGGTGAAGTCGTAACAAGGTAACCGTA
Gordonia alkanivorans	CCTGGCTCAGGACGAACGCTGGCGGCGTGCTTAACACATGCAAGTCGAACGG<br>AAAGGCCCAGCTTGCTGGGTACTCGAGTGGCGAACGGGTGAGTAACACGTGG<br>GTGATCTGCCCTGAACTTTGGGATAAGCCTGGGAAACTGGGTCTAATACCGGA<br>TATGACCTTGGAGTGCATGCTCTGGGGTGGAAAGCTTTTGCGGTTCAGGATGG<br>GCCCGCGGCCTATCAGCTTGTTGGTGGGGTAATGGCCTACCAAGGCGACGAC<br>GGGTAGCCGACCTGAGAGGGTGATCGGCCACACTGGGACTGAGACACGGCC<br>CAGACTCCTACGGGAGGCAGCAGTGGGGAATATTGCACAATGGGCGCAAGCC<br>TGATGCAGCGACGCCGCGTGAGGGATGACGGCCTTCGGGTTGTAAACCTCTT<br>TCACCAGGGACGAAGCGCAAGTGACGGTACCTGGAGAAGAAGCACCGGCCAA<br>CTACGTGCCAGCAGCCGCGGTAATACGTAGGGTGCGAGCGTTGTCCGGAATT<br>ACTGGGCGTAAAGAGCTCGTAGGCGGTTTGTCGCGTCGTCTGTGAAATTCTGC<br>AACTCAATTGTAGGCGTGCAGGCGATACGGGCAGACTTGAGTACTACAGGGG<br>AGACTGGAATTCCTGGTGTAGCGGTGAAATGCGCAGATATCAGGAGGAACAC<br>CGGTGGCGAAGGCGGGTCTCTGGGTAGTAACTGACTCTGAGGAGCGAAAGC<br>GTGGGTAGCGAACAGGATTAGATACCCTGGTAGTCCACGCCGTAAACGGTGG<br>GTACTAGGTGTGGGGCTCATTTCACGAGTTCCGTGCCGTAGCTAACGCATTAA<br>GTACCCCGCCTGGGGAGTACGGCCGCAAGGCTAAAACTCAAAGGAATTGACG<br>GGGGCCCGCACAAGCGGCGGAGCATGTGGATTAATTCGATGCAACGCGAAGA<br>ACCTTACCTGGGTTTGACATACACCAGACGCATGTAGAGATACATGTTCCCTT<br>GTGGTTGGTGTACAGGTGGTGCATGGCTGTCGTCAGCTCGTGTCGTGAGATG<br>TTGGGTTAAGTCCCGCAACGAGCGCAACCCTTGTCCTGTATTGCCAGCGGGTT<br>ATGCCGGGGACTTGCAGGAGACTGCCGGGGTCAACTCGGAGGAAGGTGGGG<br>ATGACGTCAAGTCATCATGCCCCTTATGTCCAGGGCTTCACACATGCTACAAT<br>GGCTGGTACAGAGGGCTGCGATACCGTGAGGTGGAGCGAATCCCTTAAAGCC<br>AGTCTCAGTTCGGATTGGGGTCTGCAACTCGACCCCATGAAGTCGGAGTCGC<br>TAGTAATCGCAGATCAGCAACGCTGCGGTGAATACGTTCCCGGGCCTTGTACA<br>CACCGCCCGTCACGTCATGAAAGTCGGTAACACCCGAAGCCGGTGGCCTAAC<br>CCCTTGTGGGAGGGAGCTGTCGAAGGTGGGATCGGCGATTGGGACGAAGTC<br>GTAACAAGGTAACCGTACCGGAAGGTGCGG
Nocardioides daphniae	GCCCTTCGGGGTACACGAGCGGCGAACGGGTGAGTAACACGTGAGTAATCTG<br>CCCTTCACTTCGGGATAAGCACCGGAAACGGTGTCTAATACCGGATATGAACC<br>TCCTTCGCATGTTGGGGGTTGGAAAGTTCTGGCGGTGGAGGATGTGCTCGCG<br>GCCTATCAGCTTGTTGGTGAGGTAATGGCTCACCAAGGCTTCGACGGGTAGC<br>CGGCCTGAGAGGGTGACCGGCCACACTGGGACTGAGACACGGCCCAGACTC<br>CTACGGGAGGCAGCAGTGGGGAATATTGGACAATGGGCGAAAGCCTGATCCA<br>GCAACGCCGCGTGAGGGATGACTGCCTTCGGGTTGTAAACCTCTTTCAGTAG<br>GGACGAAGCGCAAGTGACGGTACCTACAGAAGAAGCACCGGCCAACTACGTG<br>CCAGCAGCCGCGGTAATACGTAGGGTGCGAGCGTTGTCCGGAATTATTGGGC<br>GTAAAGGGCTCGTAGGCGGTTTGTCGCGTCGGGAGTGAAAACTCAGGGCTTA<br>ACTCTGAGCTTGCTTCCGATACGGGCAGACTAGAGGTATGCAGGGGAGAACG<br>GAATTCCTGGTGTAGCGGTGAAATGCGCAGATATCAGGAGGAACACCGGTGG<br>CGAAGGCGGTTCTCTGGGCATTACCTGACGCTGAGGAGCGAAAGTGTGGGGA<br>GCGAACAGGATTAGATACCCTGGTAGTCCACACCGTAAACGTTGGGCGCTAG<br>GTGTGGGGCCTATTCCATGGGTTCCGTGCCGCAGCTAACGCATTAAGCGCCC<br>CGCCTGGGGAGTACGGCCGCAAGGCTAAAACTCAAAGGAATTGACGGGGGC<br>CCGCACAAGCGGCGGAGCATGCGGATTAATTCGATGCAACGCGAAGAACCTT<br>ACCTGGGTTTGACATACACCGGAAACACCTAGAGATAGGTGCCCTTTTAGTCG<br>GTGTACAGGTGGTGCATGGCTGTCGTCAGCTCGTGTCGTGAGATGTTGGGTT<br>AAGTCCCGCAACGAGCGCAACCCTCGTCCTATGTTGCCAGCACGTCGTGGTG<br>GGGACTCATAGGAGACTGCCGGGGTCAACTCGGAGGAAGGTGGGGATGACG<br>TCAAGTCATCATGCCCCTTATGTCCAGGGCTTCACGCATGCTACAATGGCCGG<br>TACAAAGGGCTGCGATCCCGTGAGGGGGAGCGAATCCCAAAAAGCCGGTCTC<br>AGTTCGGATTGGGGTCTGCAACTCGACCCCATGAAGTCGGAGTCGCTAGTAAT

	CGCAGATCAGCAACGCTGCGGTGAATACGTTCCCGGGCCTTGTACACACCGC<br>CCGTCACGTCACGAAAGTCGGCAACACCCGAAGCCGGTGGCCCAAC
Morganella psychrotolerans	CTCCAGAGTTTGATCATGGCTCAGATTGAACGCTGGCGGCAGGCCTAACACAT<br>GCAAGTCGGGCGGTAACAGGGAGAAGCTTGCTTCTCTGCTGACGAGCGGCG<br>GACGGGTGAGTAATGTATGGGGATCTGCCTGATGGAGGGGGATAACTACTGG<br>AACGGTAGCTAATACCGCATAATGTCTCCGGACCAAAGCGGGGGACCTTCGG<br>GCCTCGCACCATCAGATGAACCCATATGGGATTAGCTAGTAGGTGAGGTAATG<br>GCTCACCTAGGCGACGATCTCTAGCTGGTCTGAGAGGATGATCAGCCACACT<br>GGGACTGAGACACGGCCCAGACTCCTACGGGAGGCAGCAGTGGGGAATATT<br>GCACAATGGGCGCAAGCCTGATGCAGCCATGCCGCGTGTATGAAGAAGGCCT<br>TAGGGTTGTAAAGTACTTTCAGTCGGGAGGAAGGTGTTGAGTTTAATATGCTC<br>AGCAATTGACGTTACCGACAGAAGAAGCACCGGCTAACTCCGTGCCAGCAGC<br>CGCGGTAATACGGAGGGTGCAAGCGTTAATCGGAATTACTGGGCGTAAAGCG<br>CACGCAGGCGGTTGATTAAGTCAGATGTGAAATCCCCGGGCTCAACCCGGGA<br>ATTGCATCTGAGACTGGTCAGCTAGAGTCTTGTAGAGGGGGGTAGAATTCCAT<br>GTGTAGCGGTGAAATGCGTAGAGATGTGGAGGAATACCGGTGGCGAAGGCG<br>GCCCCCTGGACAAAGACTGACGCTCAGGTGCGAAAGCGTGGGGAGCAAACAC<br>CGATTAAATACCCTGGTAGTCCACGCTGTAAACGATGTCGACTTGTGAGGTTG<br>TGCCCTTGAGGCGTGGCTTCCGGAGCTAACGCGTTAAGTCGACCGCCTGGGG<br>AGTACGGCCGCAAGGTTAAAACTCAAATGAATTGACGGGGGCCCGCACAAGC<br>GGTGGAGCATGTGGTTTAATTCGATGCAACGCGAAGAACCTTACCTACTCTTG<br>ACATCCAGAGAACTTAGCAGAGATGCTTTGGTGCCTTCGGGAACTCTGAGACA<br>GGTGCTGCATGGCTGTCGTCAGCTCGTGTTGTGAAATGTTGGGTTAAGTCCCG<br>CAACGAGCGCAACCCTTATCCTTTGTTGCCAGCGCGTAATGGCGGGAACTCAA<br>AGGAGACTGCCGGTGATAAACCGGAGGAAGGTGGGGATGACGTCAAGTCATC<br>ATGGCCCTTACGAGTAGGGCTACACACGTGCTACAATGGCGTATACAAAGGGA<br>AGCGACCTCGCGAGAGCAAGCGGAACTCACAAAGTACGTCGTAGTCCGGATT<br>GGAGTCTGCAACTCGACTCCATGAAGTCGGAATCGCTAGTAATCGTAGATCAG<br>AATGCTACGGTGAATACGTTCCCGGGCCTTGTACACACCGCCCGTCACACCAT<br>GGGAGTGGGTT
Gluconacetobacter tumulisoli	AGCGAACGCTGGCGGCATGCTTAACACATGCAAGTCGCACGAACCTTTCGGG<br>GTTAGTGGCGGACGGGTGAGTAACGCGTAGGGATCTGTCCATGGGTGGGGG<br>ATAACTCCGGGAAACTGGAGCTAATACCGCATGACACCTGAGGGTCAAAGGC<br>GCAAGTCGCCTGTGGAGGAACCTGCGTTCGATTAGCTAGTTGGTGGGGTAAA<br>GGCCTACCAAGGCGATGATCGATAGCTGGTCTGAGAGGATGATCAGCCACAC<br>TGGGACTGAGACACGGCCCAGACTCCTACGGGAGGCAGCAGTGGGGAATATT<br>GGACAATGGGCGCAAGCCTGATCCAGCAATGCCGCGTGTGTGAAGAAGGTCT<br>TCGGATTGTAAAGCACTTTCGACGGGGACGATGATGACGGTACCCGTAGAAG<br>AAGCCCCGGCTAACTTCGTGCCAGCAGCCGCGGTAATACGAAGGGGGCTAGC<br>GTTGCTCGGAATGACTGGGCGTAAAGGGCGCGTAGGCGGTTCAGACAGTCAG<br>ATGTGAAATTCCTGGGCTTAACCTGGGGGCTGCATTTGATACGTATGGACTAG<br>AGTGTGAGAGAGGGTTGTGGAATTCCCAGTGTAGAGGTGAAATTCGTAGATAT<br>TGGGAAGAACACCGGTGGCGAAGGCGGCAACCTGGCTCATAACTGACGCTGA<br>GGCGCGAAAGCGTGGGGAGCAAACAGGATTAGATACCCTGGTAGTCCACGCT<br>GTAAACGATGTGTGCTGGATGTTGGGTGGCTTAGCCACTCAGTGTCGTAGTTA<br>ACGCGATAAGCACACCGCCTGGGGAGTACGGCCGCAAGGTTGAAACTCAAAG<br>GAATTGACGGGGGCCCGCACAAGCGGTGGAGCATGTGGTTTAATTCGAAGCA<br>ACGCGCAGAACCTTACCAGGGCTTGACATGGGGAGGCTGTGGGCAGAGATGC<br>TCATTTCCCGCAAGGGACCTCCTGCACAGGTGCTGCATGGCTGTCGTCAGCT<br>CGTGTCGTGAGATGTTGGGTTAAGTCCCGCAACGAGCGCAACCCTCGCCTTTA<br>GTTGCCAGCATGTTTGGGTGGGCACTCTAAAGGAACTGCCGGTGACAAGCCG<br>GAGGAAGGTGGGGATGACGTCAAGTCCTCATGGCCCTTATGTCCTGGGCTAC<br>ACACGTGCTACAATGGCGGTGACAGTGGGAAGCCAGGCAGCGATGCCGAGCT<br>GATCTCAAAAAGCCGTCTCAGTTCGGATTGCACTCTGCAACTCGGGTGCATGA<br>AGTTGGAATCGCTAGTAATCGCGGATCAGCATGCCGCGGTGAATACGTTCCC<br>GGGCCTTGTACACACCGCCCGTCACACCATGGGAGTTGGTTTGACCTTAAGC<br>CGGTGAGCGAACCGCAAGGGCGCAGCCGACCACGGTCGGGTCAGCGACTGG<br>GGTGAAG
Streptomyces thermoviolaceus	GACGAACGCTGGCGGCGTGCTTAACACATGCAAGTCGAACGATGAAGCCCCT<br>TCGGGGGTGGATTAGTGGCGAACGGGTGAGTAACACGTGGGCAATCTGCCCT<br>GCACTCTGGGACAAGCCCTGGAAACGGGGTCTAATACCGGATACGACACAGG<br>GAGGCATCTCCTCTGTGTGGAAAGCTCCGGCGGTGCAGGATGAGCCCGCGG<br>CCTATCAGCTTGTTGGTGAGGTAACGGCTCACCAAGGCGACGACGGGTAGCC

	GGCCTGAGAGGGCGACCGGCCACACTGGGACTGAGACACGGCCCAGACTCC<br>TACGGGAGGCAGCAGTGGGGAATATTGCACAATGGGCGCAAGCCTGATGCAG<br>CGACGCCGCGTGAGGGATGACGGCCTTCGGGTTGTAAACCTCTTTCAGCAGG<br>GAAGAAGCGCAAGTGACGGTACCTGCAGAAGAAGCGCCGGCTAACTACGTGC<br>CAGCAGCCGCGGTAATACGTAGGGCGCAAGCGTTGTCCGGAATTATTGGGCG<br>TAAAGAGCTCGTAGGCGGCTTGTCGCGTCGGTTGTGAAAGCCCGGGGCTTAA<br>CTCCGGGTCTGCAGTCGATACGGGCAGGCTAGAGTTCGGTAGGGGAGATCG<br>GAATTCCTGGTGTAGCGGTGAAATGCGCAGATATCAGGAGGAACACCGGTGG<br>CGAAGGCGGATCTCTGGGCCGATACTGACGCTGAGGAGCGAAAGCGTGGGG<br>AGCGAACAGGATTAGATACCCTGGTAGTCCACGCCGTAAACGGTGGGCACTA<br>GGTGTGGGCAGCATTCCACGTTGTCCGTGCCGTAGCTAACGCATTAAGTGCC<br>CCGCCTGGGGAGTACGGCCGCAAGGCTAAAACTCAAAGGAATTGACGGGGG<br>CCCGCACAAGCGGCGGAGCATGTGGCTTAATTCGACGCAACGCGAAGAACCT<br>TACCAAGGCTTGACATACACCGGAAACATCCAGAGATGGGTGCCCCCTTGTG<br>GTCGGTGTACAGGTGGTGCATGGCTGTCGTCAGCTCGTGTCGTGAGATGTTG<br>GGTTAAGTCCCGCAACGAGCGCAACCCTTGTCCCGTGTTGCCAGCAGGCCCT<br>TGTGGTGCTGGGGACTCACGGGAGACCGCCGGGGTCAACTCGGAGGAAGGT<br>GGGGACGACGTCAAGTCATCATGCCCCTTATGTCTTGGGCTGCACACGTGCTA<br>CAATGGCCGGTACAAAGAGCTGCGATACCGCGAGGTGGAGCGAATCTCAAAA<br>AGCCGGTCTCAGTTCGGATTGGGGTCTGCAACTCGACCCCATGAAGTCGGAG<br>TCGCTAGTAATCGCAGATCAGCATTGCTGCGGTGAATACGTTCCCGGGCCTTG<br>TACACACCGCCCGTCACGTCACGAAAGTCGGTAACACCCGAAGCCGGTGGCC<br>CAACCCCTTGTGGGAGGGAGCTGTCGAAGGTGGGACTGGCGATTGGGACGA<br>AGTCGTAACAAGGTAGCCGTACCGGAAGGTGC
Microbacterium esteraromaticum	TGGCGGCGTGCTTAACACATGCAAGTCGAACGATGAAGCCCAGCTTGCTGGG<br>TGGATTAGTGGCGAACGGGTGAGTAACACGTGAGCAACCTGCCCCTGACTCT<br>GGGATAAGCGCTGGAAACGGCGTCTAATACTGGATATGTCCCGTCACCGCAT<br>GGTGTGCGGGTGGAAAGATTTTTCGGTTGGGGATGGGCTCGCGGCCTATCAG<br>CTTGTTGGTGAGGTAATGGCTCACCAAGGCGTCGACGGGTAGCCGGCCTGAG<br>AGGGTGACCGGCCACACTGGGACTGAGACACGGCCCAGACTCCTACGGGAG<br>GCAGCAGTGGGGAATATTGCACAATGGGCGGAAGCCTGATGCAGCAACGCCG<br>CGTGAGGGATGACGGCCTTCGGGTTGTAAACCTCTTTTAGCAGGGAAGAAGC<br>GAAAGTGACGGTACCTGCAGAAAAAGCACCGGCTAACTACGTGCCAGCAGCC<br>GCGGTAATACGTAGGGTGCAAGCGTTATCCGGAATTATTGGGCGTAAAGAGCT<br>CGTAGGCGGTCTGTCGCGTCTGCTGTGAAATTCCGAGGCTCAACCTCGGGCT<br>TGCAGTGGGTACGGGCAGACTAGAGTGCGGTAGGGGAGATTGGAATTCCTGG<br>TGTAGCGGTGGAATGCGCAGATATCAGGAGGAACACCGATGGCGAAGGCAGA<br>TCTCTGGGCCGTAACTGACGCTGAGGAGCGAAAGGGTGGGGAGCAAACAGG<br>CTTAGATACCCTGGTAGTCCACCCCGTAAACGTTGGGAACTAGTTGTGGGGTC<br>CTTTCCACGGATTCCGTGACGCAGCTAACGCATTAAGTTCCCCGCCTGGGGA<br>GTACGGCCGCAAGGCTAAAACTCAAAGGAATTGACGGGGACCCGCACAAGCG<br>GCGGAGCATGCGGATTAATTCGATGCAACGCGAAGAACCTTACCAAGGCTTGA<br>CATACACGAGAACGGGCCAGAAATGGTCAACTCTTTGGACACTCGTGAACAGG<br>TGGTGCATGGTTGTCGTCAGCTCGTGTCGTGAGATGTTGGGTTAAGTCCCGCA<br>ACGAGCGCAACCCTCGTTCTATGTTGCCAGCACGTAATGGTGGGAACTCATGG<br>GATACTGCCGGGGTCAACTCGGAGGAAGGTGGGGATGACGTCAAATCATCAT<br>GCCCCTTATGTCTTGGGCTTCACGCATGCTACAATGGCCGGTACAATGGGCTG<br>CAATACCGTAAGGTGGAGCGAATCCCAAAAAGCCGGTCCCAGTTCGGATTGA<br>GGTCTGCAACTCGACCTCATGAAGTCGGAGTCGCTAGTAATCGCAGATCAGCA<br>ACGCTGCGGTGAATACGTTCCCGGGTCTTGTACACACCGCCCGTCAAGTCAT<br>GAAAGTCGGTAACACCTGAAGCCGGTGGCCTAACCCCTTGTGGGAGGGAGCT<br>GTCGAAGGTGGGATCGGTAATTAGGACTAAGTCGTAACAAGGTAGCCGTACC<br>G
Planococcus kocurii	GACGAACGCTGGCGGCGTGCCTAATACATGCAAGTCGAGCGGAACTTTGGGA<br>GCTTGCTCCCATTGTTTAGCGGCGGACGGGTGAGTAACACGTGGGCAACCTG<br>CCCTGCAGATCGGGATAACTCCGGGAAACCGGTGCTAATACCGAATAGTTTGC<br>GGCCTCTCCTGAGGCTGTACGGAAAGACGGTTTCGGCTGTCACTGCAGGATG<br>GGCCCGCGGCGCATTAGCTAGTTGGTGGGGTAATGGCCTACCAAGGCGACGA<br>TGCGTAGCCGACCTGAGAGGGTGATCGGCCACACTGGGACTGAGACACGGC<br>CCAGACTCCTACGGGAGGCAGCAGTAGGGAATCTTCCGCAATGGACGAAAGT<br>CTGACGGAGCAACGCCGCGTGAGTGACGAAGGTTTTCGGATCGTAAAACTCT<br>GTTGTGAGGGAAGAACAAGTACCAAGTAACTACTGGTACCTTGACGGTACCTC<br>ACCAGAAAGCCACGGCTAACTACGTGCCAGCAGCCGCGGTAATACGTAGGTG<br>GCAAGCGTTGTCCGGAATTATTGGGCGTAAAGCGCGCGCAGGCGGTTCCTTA<br>AGTCCGATGTGAAAGCCCACGGCTCAACCGTGGAGGGTCATTGGAAACTGGG

	GAACTTGAGTGCAGAAGAGGAAAGTGGAATTCCATGTGTAGCGGTGAAATGC<br>GTAGAGATGTGGAGGAACACCAGTGGCGAAGGCGACTTTCTGGTCTGTAACT<br>GACGCTGAGGCGCGAAAGCGTGGGGAGCAAACAGGATTAGATACCCTGGTAG<br>TCCACGCCGTAAACGATGAGTGCTAAGTGTTAGGGGGTTTCCGCCCCTTAGTG<br>CTGCAGCTAACGCATTAAGCACTCCGCCTGGGGAGTACGGCCGCAAGGCTGA<br>AACTCAAAGGAATTGACGGGGGCCCGCACAAGCGGTGGAGCATGTGGTTTAA<br>TTCGAAGCAACGCGAAGAACCTTACCAGGTCTTGACATCCCACTGACCGGTGT<br>AGAGATACGCTTTTCCCTTCGGGGACAGTGGTGACAGGTGGTGCATGGTTGT<br>CGTCAGCTCGTGTCGTGAGATGTTGGGTTAAGTCCCGCAACGAGCGCAACCC<br>TTGATCTTAGTTGCCAGCATTCAGTTGGGCACTCTAAGGTGACTGCCGGTGAC<br>AAACCGGAGGAAGGTGGGGATGACGTCAAATCATCATGCCCCTTATGACCTG<br>GGCTACACACGTGCTACAATGGACGGTACAAAGGGCTGCAACCCCGCGAGGG<br>CAAGCCAATCCCAGAAAACCGTTCTCAGTTCGGATTGCAGGCTGCAACTCGCC<br>TGCATGAAGCCGGAATCGCTAGTAATCGTGGATCAGCATGCCACGGTGAATAC<br>GTTCCCGGGCCTTGTACACACCGCCCGTCACACCACGAGAGTTTGTAACACC<br>CGAAGTCGGTGAGGTAACCCTTGTGGAGCCAGCCGCCGAAGGTGGGACAGAT<br>GATTGGGGTGAAG
Psychrobacter okhotskensis	GATTGAACGCTGGCGGCAGGCTTAACACATGCAAGTCGAGCGGAAACGATGG<br>TAGCTTGCTACCAGGCGTCGAGCGGCGGACGGGTGAGTAATACTTAGGAATC<br>TACCTAGTAGTGGGGGATAGCTCGGGGAAACTCGAATTAATACCGCATACGAC<br>CTACGGGAGAAAGGGGGCAGTTTACTGCTCTCGCTATTAGATGAGCCTAAGTC<br>GGATTAGCTAGATGGTGGGGTAAAGGCCTACCATGGCGACGATCTGTAGCTG<br>GTCTGAGAGGATGATCAGCCACACCGGGACTGAGACACGGCCCGGACTCCTA<br>CGGGAGGCAGCAGTGGGGAATATTGGACAATGGGGGAAACCCTGATCCAGCC<br>ATGCCGCGTGTGTGAAGAAGGCCTTTTGGTTGTAAAGCACTTTAAGCAGTGAA<br>GAAGACTCCGTGGTTAATACCCACGGACGATGACATTAGCTGCAGAATAAGCA<br>CCGGCTAACTCTGTGCCAGCAGCCGCGGTAATACAGAGGGTGCAAGCGTTAA<br>TCGGAATTACTGGGCGTAAAGGGAGCGTAGGTGGCTCGATAAGTCAGATGTG<br>AAATCCCCGGGCTCAACCTGGGAACTGCATCTGATACTGTTGAGCTAGAGTAT<br>GTGAGAGGAAGGTAGAATTCCAGGTGTAGCGGTGAAATGCGTAGAGATCTGG<br>AGGAATACCGATGGCGAAGGCAGCCTTCTGGCATAATACTGACACTGAGGCT<br>CGAAAGCGTGGGTAGCAAACAGGATTAGATACCCTGGTAGTCCACGCCGTAA<br>ACGATGTCTACTAGTCGTTGGGTCCCTTGAGGACTTAGTGACGCAGCTAACGC<br>AATAAGTAGACCGCCTGGGGAGTACGGCCGCAAGGTTAAAACTCAAATGAATT<br>GACGGGGGCCCGCACAAGCGGTGGAGCATGTGGTTTAATTCGATGCAACGCG<br>AAGAACCTTACCTGGTCTTGACATATCTAGAATCCTGCAGAGATGCGGGAGTG<br>CCTTCGGGAATTAGAATACAGGTGCTGCATGGCTGTCGTCAGCTCGTGTCGTG<br>AGATGTTGGGTTAAGTCCCGCAACGAGCGCAACCCTTGTCCTTAGTTACCAGC<br>GGGTTAAGCCGGGAACTCTAAGGATACTGCCAGTGACAAACTGGAGGAAGGC<br>GGGGACGACGTCAAGTCATCATGGCCCTTACGACCAGGGCTACACACGTGCT<br>ACAATGGTAGGTACAGAGGGCAGCTACACAGCGATGTGATGCGAATCTCAAAA<br>AGCCTATCGTAGTCCAGATTGGAGTCTGCAACTCGACTCCATGAAGTAGGAAT<br>CGCTAGTAATCGCGGATCAGAATGCCGCGGTGAATACGTTCCCGGGCCTTGT<br>ACACACCGCCCGTCACACCATGGGAGTTGATTGCACCAGAAGTGGATAGCTTA<br>ACCTTCGGGGGAGCGTTCACCACGGTGTGGTTGATGACTGGGGTGAAGTCGT<br>AACAAGGTAGCCGTAGGGGAACC
Pseudomonas fluorescens	AGTTTGATCCTGGCTCAGATTGAACGCTGGCGGCAGGCCTAACACATGCAAGT<br>CGAGCGGTAGAGAGAAGCTTGCTTCTCTTGAGAGAGGCGGACGGGTGAGTAA<br>AGCCTAGGAATCTGCCTGGTAGTGGGGGATAACGTTCGGAAACGGACGCTAA<br>TACCGCATACGTCCTACGGGAGAAAGCAGGGGACCTTCGGGCCTTGCGCTAT<br>CAGATGAGCCTAGGTCGGATTAGCTAGTTGGTGAGGTAATGGCTCACCAAGG<br>CGACGATCCGTAACTGGTCTGAGAGGATGATCAGTCACACTGGAACTGAGACA<br>CGGTCCAGACTCCTACGGGAGGCAGCAGTGGGGAATATTGGACAATGGGCGA<br>AAGCCTGATCCAGCCATGCCGCGTGTGTTAAGAAGGTCTTCGGGTTGTAAAGC<br>ACTTTAAGTTGGGAGGAAGGGCATTAACCTAATACGTTAGTGTTTTGACGTTAC<br>CGACAGAATAAGCACCGGCTAACTCTGTGCCAGCAGCCGCGGTAATACAGAG<br>GGTGCAAGCGTTAATCGGAATTACTGGGCGTAAAGCGCGCGTAGGTGGTTTG<br>TTAAGTTGGATGTGAAATCCCCGGGCTCAACCTGGGAACTGCATTCAAAACTG<br>ACTGACTAGAGTATGGTAGAGGGTGGTGGAATTTCCTGTGTAGCGGTGAAATG<br>CGCAGATATAGGAAGGAACACCAGTGGCGAAGGCGACCACCTGGACTAATAC<br>TGACACTGAGGTGCGAAAGCGTGGGGAGCAAACAGGATTAGATACCCTGGTA<br>GTCCACGCCGTAAACGATGTCAACTAGCCGTTGGGAGCCTTGAGCTCTTAGTG<br>GCGCAGCTAACGCATTAAGTTGACCGCCTGGGGAGTACGGCCGCAAGGTTAA<br>AACTCAAATGAATTGACGGGGGCCCGCACAAGCGGTGGAGCATGTGGTTTAA<br>TTCGAAGCAACGCGAAGAGCCTTACCAGGCCTTGACATCCAATGAACTTTCTA

	GAGATAGATTGGTGCCTTCGGGAACATTGAGACAGGTGCTGCATGGCTGTCG<br>TCAGCTCGTGTCGTGAGATGTTGGGTTAAGTCCCGTAACGAGCGCAACCCTTG<br>TCCTTAGTTACCAGCACGTAATGGTGGGCACTCTAAGGAGACTGCCGGTGACA<br>AACCGGAGGAAGGTGGGGATGACGTCAAGTCATCATGGCCCTTACGGCCTGG<br>GCTACACACGTGCTACAATGGTCGGTACAGAGGGTTGCCAAGCCGCGAGGTG<br>GAGCTAATCCCTCAAAACCGATCGTAGTCCGGATCGTAGTCTGCAACTCGACT<br>GCGTGAAGTCGGAATCGCTAGTAATCGCGAATCAGAATGTCGCGGTGAATAC<br>GTTCCCGGGCCTTGTACACACCGCCCGTCACACCATGGGAGTGGGTTGCACC<br>AGAAGTAGCTAGTCTAACCTTCGGGAGGGCGGTTACCACGGTGTGATTCATGA<br>CTGGGGTGAAGTCGTAACAAGGTAGCCGTAGGGGAACCTGCGGCTGGATCAC<br>CTCCTT
Ligilactobacillus equi	AACGCTTTTTCTTATCACCGTAGCTTGCTACACCGATAAGAAATTGAGTGGCGA<br>ACGGGTGAGTAACACGTGGGTAACCTGCCCTAAAGAGGGGGATAACACTTGG<br>AAACAGGTGCTAATACCGCATATCTCTTAGAACCGCATGGTTCTGGGATGAAA<br>GGTGGCGTAAGCTATCACTTTAGGATGGACCCGCGGCGTATTAGCTTGTTGGT<br>GGGGTAATGGCCTACCAAGGCGATGATACGTAGCCGAACTGAGAGGTTGATC<br>GGCCACATTGGGACTGAGACACGGCCCAAACTCCTACGGGAGGCAGCAGTAG<br>GGAATCTTCCACAATGGACGCAAGTCTGATGGAGCAACGCCGCGTGAATGAA<br>GAAGGCCTTCGGGTCGTAAAATTCTGTTGTTAGAGAAGAACATGCAGGAGAGT<br>AACTGTTCTTGTATTGACGGTATCTAACCAGAAAGCCACGGCTAACTACGTGC<br>CAGCAGCCGCGGTAATACGTAGGTGGCAAGCGTTGTCCGGATTTATTGGGCG<br>TAAAGGGAACGCAGGCGGTTTTTTAAGTCTGATGTGAAAGCCTTCGGCTCAAC<br>CGAAGAATTGCATTGGAAACTGGAAGACTTGAGTGCAGAAGAGGAGAGTGGA<br>ACTCCATGTGTAGCGGTGGAATGCGTAGATATATGGAAGAACACCAGTGGCGA<br>AAGCGGCTCTCTGGTCTGTAACTGACGCTGAGGTTCGAAAGCGTGGGTAGCA<br>AACAGGATTAGATACCCTGGTAGTCCACGCCGTAAACGATGAATACTAAGTGT<br>TGGAGGGTTTCCGCCCTTCAGTGCTGCAGCTAACGCAATAAGTATTCCGCCTG<br>GGGAGTACGGCCGCAAGGCTGAAACTCAAAGGAATTGACGGGGGCCCGCAC<br>AAGCGGTGGAGCATGTGGTTTAATTCGAAGCAACGCGAAGAACCTTACCAGGT<br>CTTGACATCTTTTGACCATCTGAGAGATCAGATTTTCCCTTCGGGGACAAAATG<br>ACAGGTGGTGCATGGCTGTCGTCAGCTCGTGTCGTGAGATGTTGGGTTAAGT<br>CCCGCAACGAGCGCAACCCTTGTTGTCAGTTGCCAGCATTAAGTTGGGCACTC<br>TGGCGAGACTGCCGGTGACAAACCGGAGGAAGGTGGGGACGACGTCAAGTC<br>ATCATGCCCCTTATGACCTGGGCTACACACGTGCTACAATGGACGGTACAACG<br>AGTYGCTAACCCGCGAGGGTACGCTAATCTCTTAAAGCCGTTCTCAGTTCGGA<br>TTGTAGGCTGCAACTCGCCTACATGAAGTCGGAATCGCTAGTAATCGCGAATC<br>AGCATGTCGCGGTGAATACGTTCCCGGGCCTTGTACACACCGCCCGTCACAC<br>CATGAGAGTTTGTAACACCCAAAGCCGGTGGGGTAACCTTTTAGGAGCTAGCC<br>GTCTAAGGTGGG
Amycolatopsis lurida	GACGAACGCTGGCGGCGTGCTTAACACATGCAAGTCGAACGATGAAGCCTTT<br>CGGGGTGGATTAGTGGCGAACGGGTGAGTAACACGTGGGCAATCTGCCCTGT<br>ACTTTGGGATAAGCCTGGGAAACTGGGTCTAATACCGGATACGACTACTGATC<br>GCATGGTTGGTGGTGGAAAGCTCCGGCGGTACAGGATGAGCCCGCGGCCTAT<br>CAGCTTGTTGGTGGGGTAATGGCCTACCAAGGCGACGACGGGTAGCCGGCCT<br>GAGAGGGTGACCGGCCACACTGGGACTGAGACACGGCCCAGACTCCTACGG<br>GAGGCAGCAGTGGGGAATATTGCACAATGGGCGCAAGCCTGATGCAGCGACG<br>CCGCGTGAGGGATGACGGCCTTCGGGTTGTAAACCTCTTTCGCCAGGGACGA<br>AGCGCAAGTGACGGTACCTGGATAAGAAGCACCGGCTAACTACGTGCCAGCA<br>GCCGCGGTAATACGTAGGGTGCGAGCGTTGTCCGGAATTATTGGGCGTAAAG<br>AGCTCGTAGGCGGTTTGTCGCGTCGTTCGTGAAAACTCCACGCTTAACGTGGA<br>GCGTGCGGGCGATACGGGCAGACTTGAGTTCGGTAGGGGAGACTGGAATTCC<br>TGGTGTAGCGGTGAAATGCGCAGATATCAGGAGGAACACCGGTGGCGAAGGC<br>GGGTCTCTGGGCCGATACTGACGCTGAGGAGCGAAAGCGTGGGGAGCGAAC<br>AGGATTAGATACCCTGGTAGTCCACGCTGTAAACGTTGGGCGCTAGGTGTGG<br>GCGACATCCACGTTGTCCGTGCCGTAGCTAACGCATTAAGCGCCCCGCCTGG<br>GGAGTACGGCCGCAAGGCTAAAACTCAAAGGAATTGACGGGGGCCCGCACAA<br>GCGGCGGAGCATGTGGATTAATTCGATGCAACGCGAAGAACCTTACCTGGGC<br>TTGACATGCGCCAGACATCCCTAGAGATAGGGCTTCCCTTGTGGTTGGTGTAC<br>AGGTGGTGCATGGCTGTCGTCAGCTCGTGTCGTGAGATGTTGGGTTAAGTCC<br>CGCAACGAGCGCAACCCTTATCCTACGTTGCCAGCGCGTTATGGCGGGGACT<br>CGTGGGAGACTGCCGGGGTCAACTCGGAGGAAGGTGGGGATGACGTCAAGT<br>CATCATGCCCCTTATGTCCAGGGCTTCACACATGCTACAATGGCTGGTACAGA<br>GGGCTGCGATACCGCGAGGTGGAGCGAATCCCTTAAAGCCGGTCTCAGTTCG<br>GATCGCAGTCTGCAACTCGACTGCGTGAAGTCGGAGTCGCTAGTAATCGCAG<br>ATCAGCAACGCTGCGGTGAATACGTTCCCGGGCCTTGTACACACCGCCCGTC

	ACGTCATGAAAGTCGGTAACACCCGAAGCCCATGGCCCAACCCGTAAGGGGGGGAGTGGTCGAAGGTGGGACTGGCGATTGGGACGAAGTCGTAACAAGGTAGCCGTACCGGAAGGTGCGGCTGGATCACCTCCTT
Pseudonocardia thermophila	AGAGTTTGATCATGGCTCAGGACGAACGCTGGCGGCGCGCTTAACACATGCAAGTCGAGCGGTAAGGCTCCTTGCGGGAGTACACGAGCGGCGAACGGGTGAGTAACACGTGGGCAACCTGCCCCCCAGCTCTGGGATAAGCCTGGGAAACTGGGTCTAATACCGGATATGACCGCTGCCGCATGGTCTGGTGGTGGAAAGTTTTTTCGGCTGGGGATGGCCCGCGGCCTATCAGCTTGTTGGTGGGGTAATGGCCTACCAAGGCGACGACGGGTAGCCGGCCTGAGAGGGCGACCGGCCACACTGGGACTGAGACACGGCCCAGACTCCTACGGGAGGCAGCAGTGGGGAATATTGCGCAATGGGCGAAAGCCTGACGCAGCGACGCCGCGTGGGGGATGAAGGCCTTCGGGTTGTAAACCTCTTTCGACAGGGACGAAGCCTTTCGGGGTGACGGTACCTGTAGAAGAAGCACCGGCCAACTACGTGCCAGCAGCCGCGGTAATACGTAGGGTGCGAGCGTTGTCCGGAATTATTGGGCGTAAAGAGCTCGTAGGCGGTGTGTCGCGTCGGCCGTGAAAACTTTCAGCTTAACTGGGAGCTTGCGGTCGATACGGGCATCACTAGAGTTCGGCAGGGGAGACTGGAATTCAAGGTGTAGCGGTGAAATGCGCAGATATCTTGAGGAACACCGGTGGCGAAGGCGGGTCTCTGGGCCGATACTGACGCTGAGGAGCGAAAGCGTGGGGAGCGAACAGGATTAGATACCCTGGTAGTCCACGCCGTAAACGGTGGGCGCTAGGTGTGGGGGCCATTCCACGGTCTCTGTGCCGCAGCTAACGCATTAAGCGCCCCGCCTGGGGAGTACGGCCGCAAGGCTAAAACTCAAAGGAATTGACGGGGGCCCGCACAAGCGGCGGAGCATGTGGATTAATTCGATGCAACGCGAAGAACCTTACCTGGGTTTGACATGCACCGGACGCGTCTAGAGATAGGCGTTCCCTTGTGGCCGGTGTGCAGGTGGTGCATGGCTGTCGTCAGCTCGTGTCGTGAGATGTTGGGTTAAGACCCGCAATGAGCGCAACCCTNGTCCCATGTTGCCAGCACGTTATGGTGGGGACTCATGGGAGACTGCCGGGGTCAACTCGGAGGAAGGTGGGGATGACGTCAAGTCATCATGCCCCTTATGCCCAGGGCTTCACACATGCTACAATGGCCAGTACAGAGGGCTGCGAGACCGTGAGGTGGAGCGAATCCCTTAAAGCTGGTCTCAGTTCGGATCGGGGTCTGCAACTCGACCCCGTGAAGTTGGAGTCGCTAGTAATCGCAGATCAGCATTGCTGCGGTGAATACGTTCCCGGGCCTTGTACACACCGCCCGTCACGTCATGAAAGTTGGTAACACCCGAAGCCGATGGCCTAACCTCTTCGGGGGAGGGAGTCGTCGAAGGTGGGACCGGCGATTGGGACGAAGTCGTAACAAGGTAGCCGTACCGGAAGGTGCGGCTGGATCACCTCCTT
Metabacillus galliciensis	TTGCTCTCAAAGATTAGCGGCGGACGGGTGAGTAACACGTGGGTAACCTGCCTGTAAGATTGGGATAACTCCGGGAAACCGGAGCTAATACCGGATAACATTTTGAACCGCATGGTTCGAAATTGAAAGGTGGCTTTTGCTACCACTTACAGATGGACCCGCGGCGCATTAGCTAGTTGGTGAGGTAATGGCTCACCAAGGCAACGATGCGTAGCCGACCTGAGAGGGTGATCGGCCACACTGGGACTGAGACACGGCCCAGACTCCTACGGGAGGCAGCAGTAGGGAATCTTCCGCAATGGACGAAAGTCTGACGGAGCAACGCCGCGTGAGTGATGAAGGCCTTCGGGTCGTAAAGCTCTGTTGTTAGGGAAGAACAAGTACCGTTCAAATAGGGCGGTACCATGACGGTACCTAACCAGAAAGCCACGGCTAACTACGTGCCAGCAGCCGCGGTAATACGTAGGTGGCAAGCGTTGTCCGGAATTATTGGGCGTAAAGGGCTCGCAGGCGGTTCCTTAAGTCTGATGTGAAAGCCCACGGCTCAACCGTGGAGGGTCATTGGAAACTGGGGAACTTGAGTACAGAAGAGGAGAGTG
Herbaspirillum seropedicae	GGAGTTTGATTATGGCTCAGATTGAACGCTGGCGGCATGCCTTACACATGCAAGTCGAACGGCAGCATAGGAGCTTGCTCCTGATGGCGAGTGGCGAACGGGTGAGTAATATATCGGAACGTGCCCTAGAGTGGGGGATAACTAGTCGAAAGATTAGCTAATACCGCATACGATCTACGGATGAAAGTGGGGGATCGCAAGACCTCATGCTCCTGGAGCGGCCGATATCTGATTAGCTAGTTGGTGGGGTAAAAGCCTACCAAGGCGACGATCAGTAGCTGGTCTGAGAGGACGACCAGCCACACTGGGACTGAGACACGGCCCAGACTCCTACGGGAGGCAGCAGTGGGGAATTTTGGACAATGGGGGCAACCCTGATCCAGCAATGCCGCGTGAGTGAAGAAGGCCTTCGGGTTGTAAAGCTCTTTTGTCAGGGAAGAAACGGTTTTGGCTAATATCCAGAACTAATGACGGTACCTGAAGAATAAGCACCGGCTAACTACGTGCCAGCAGCCGCGGTAATACGTAGGGTGCAAGCGTTAATCGGAATTACTGGGCGTAAAGCGTGCGCAGGCGGTTGTGTAAGACAGATGTGAAATCCCCGGGCTCAACCTGGGAATTGCATTTGTGACTGCACGGCTAGAGTGTGTCAGAGGGGGGTAGAATTCCACGTGTAGCAGTGAAATGCGTAGATATGTGGAGGAATACCGATGGCGAAGGCAGCCCCCTGGGATAACACTGACGCTCATGCACGAAAGCGTGGGGAGCAAACAGGATTAGATACCCTGGTAGTCCACGCCCTAAACGATGTCTACTAGTTGTCGGGTCTTAATTGACTTGGTAACGCAGCTAACGCGTGAAGTAGACCGCCTGGGGAGTACGGTCGCAAGATTAAAACTCAAAGGAATTGACGGGGACCCGCACAAGCGGTGGATGATGTGGAT

	TAATTCGATGCAACGCGAAAAACCTTACCTACCCTTGACATGGTCGGAATCCT<br>GAAGAGATTTGGGAGTGCTCGAAAGAGAACCGGCGCACAGGTGCTGCATGGC<br>TGTCGTCAGCTCGTGTCGTGAGATGTTGGGTTAAGTCCCGCAACGAGCGCAA<br>CCCTTGTCATTAGTTGCTACGAAAGGGCACTCTAATGAGACTGCCGGTGACAA<br>ACCGGAGGAAGGTGGGGATGACGTCAAGTCCTCATGGCCCTTATGGGTAGGG<br>CTTCACACGTCATACAATGGTACATACAGAGGGCCGCCAACCCGCGAGGGGG<br>AGCTAATCCCAGAAAGTGTATCGTAGTCCGGATTGGAGTCTGCAACTCGACTC<br>CATGAAGTTGGAATCGCTAGTAATCGCGGATCAGCATGTCGCGGTGAATACGT<br>TCCCGGGTCTTGTACACACCGCCCGTCACACCATGGGAGCGGGTTTTACCAG<br>AAGTGGGTAGCCTAACCGCAAGGAGGGCGCTCACCACGGTAGGATTCGTGAC<br>TGGGGTGAAGTCGTAACAAGGTAGCCGTATCGGAAGGTGCGGCTGGATCACC<br>TCCTTT
Escherichia coli	ATTGAACGCTGGCGGCAGGCCTAACACATGCAAGTCGAACGGTAACAGGAAG<br>CAGCTTGCTGCTTTGCTGACGAGTGGCGGACGGGTGAGTAATGTCTGGGAAA<br>CTGCCNGATGGAGGGGGATAACTACTGGAAACGGTAGCTAATACCGCATAAC<br>GTCGCAAGACCAAAGAGGGGGACCTTAGGGCCTCTTGCCATCGGATGTGCCC<br>AGATGGGATTAGCTAGTAGGTGGGGTAACGGCTCACCTAGGCGACGATCCCT<br>AGCTGGTCTGAGAGGATGACCAGCCACACTGGAACTGAGACACGGTCCAGAC<br>TCCTACGGGAGGCAGCAGTGGGGAATATTGCACAATGGGCGCAAGCCTGATG<br>CAGCCATGCCGCGTGTATGAAGAAGGCCTTCGGGTTGTAAAGTACTTTCAGCG<br>GGGAGGAAGGGAGTAAAGTTAATACCTTTGCTCATTGACGTTACCCGCAGAAG<br>AAGCACCGGCTAACTCCGTGCCAGCAGCCGCGGTAATACGGAGGGTGCAAGC<br>GTTAATCGGAATTACTGGGCGTAAAGCGCACGCAGGCGGTTTGTTAAGTCAGA<br>TGTGAAATCCCCGGGCTCAACCTGGGAACTGCATCTGATACTGGCAAGCTTGA<br>GTCTCGTAGAGGGGGGNAGAATTCCAGGTGTAGCGGTGAAATGCGTAGAGAT<br>CTGGAGGAATACCGGTGGCGAAGGCGGCCCCCTGGACGAAGACTGACGCTC<br>AGGTGCGAAAGCGTGGGGAGCAAACAGGATTAGATACCCTGGTAGTCCACGC<br>CGTAAACGATGTCGACTTGGAGGTTGTGCCCTTGAGGCGTGGCTTCCGGAGC<br>TAACGCGTTAAGTCGACCGCCTGGGGAGTACGGCCGCAAGGTTAAAACTCAA<br>ATGAATTGACGGGGGCCCGCACAAGCGGTGGAGCATGTGGTTTAATTCGATG<br>CAACGCGAAGAACCTTACCTGGTCTTGACATCCACGGAAGTTTTCAGAGATGA<br>GAATGTGCCTTCGGGAACCGTGAGACAGGTGCTGCATGGCTGTCGTCAGCTC<br>GTGTTGTGAAATGTTGGGTTAAGTCCCGCAACGAGCGCAACCCTTATCCTTTG<br>TTGCCAGCGGTCCGGCCGGGAACTCAAAGGAGACTGCCAGTGATAAACTGGA<br>GGAAGGTGGGGATGACGTCAAGTCATCATGGCCCTTACGACCAGGGCTACAC<br>ACGTGCTACAATGGCGCATACAAAGAGAAGCGACCTCGCGAGAGCAAGCGNA<br>CCTCATAAAGTGCGTCGTAGTCCGGATTGGAGTCTGCAACTCGACTCCATGAA<br>GTCGGAATCGCTAGTAATCGTGGATCAGAATGCCACGGTGAATACGTTCCCGG<br>GCCTTGTACACACCGCCCGTCACACCATGGGAGTGGGTTGCAAAAGAAGTAG<br>GTAGCTTAACCTTCGGGAGGGCGCTTACCACTTTGTGATTCATGACTGGGGTG<br>AAG
Chlamydia trachomatis	CTGAGAATTTGATCTTGGTTCAGATTGAACGCTGGCGGCGTGGATGAGGCATG<br>CAAGTCGAACGGAGCAATTGTTTCGACGATTGTTTAGTGGCGGAAGGGTTAGT<br>AATGCATAGATAATTTGTCCTTAACTTGGGAATAACGGTTGGAAACGGCCGCTA<br>ATACCGAATGTGGCGATATTTGGGCATCCGAGTAACGTTAAAGAAGGGGATCT<br>TAGGACCTTTCGGTTAAGGGAGAGTCTATGTGATATCAGCTAGTTGGTGGGGT<br>AAAGGCCTACCAAGGCTATGACGTCTAGGCGGATTGAGAGATTGGCCGCCAA<br>CACTGGGACTGAGACACTGCCCAGACTCCTACGGGAGGCTGCAGTCGAGAAT<br>CTTTCGCAATGGACGGAAGTCTGACGAAGCGACGCCGCGTGTGTGATGAAGG<br>CTCTAGGGTTGTAAAGCACTTTCGCTTGGGAATAAGAGAAGACGGTTAATACC<br>CGCTGGATTTGAGCGTACCAGGTAAAGAAGCACCGGCTAACTCCGTGCCAGC<br>AGCTGCGGTAATACGGAGGGTGCTAGCGTTAATCGGATTTATTGGGCGTAAAG<br>GGCGTGTAGGCGGAAAGGTAAGTTAGTTGTCAAAGATCGGGGCTCAACCCCG<br>AGTCGGCATCTAATACTATTTTTCTAGAGGATAGATGGAGAAAAGGGAATTTCA<br>CGTGTAGCGGTGAAATGCGTAGATATGTGGAAGAACACCAGTGGCGAAGGCG<br>CTTTTCTAATTTATACCTGACGCTAAGGCGCGAAAGCAAGGGGAGCAAACAGG<br>ATTAGATACCCTGGTAGTCCTTGCCGTAAACGATGCATACTTGATGTGGATGG<br>TCTCAACCCCATCCGTGTCGGAGCTAACGCGTTAAGTATGCCGCCTGAGGAGT<br>ACACTCGCAAGGGTGAAACTCAAAAGAATTGACGGGGGCCCGCACAAGCAGT<br>GGAGCATGTGGTTTAATTCGATGCAACGCGAAGGACCTTACCTGGGTTTGACA<br>TGTATATGACCGCGGCAGAAATGTCGTTTTCCGCAAGGACATATACACAGGTG<br>CTGCATGGCTGTCGTCAGCTCGTGCCGTGAGGTGTTGGGTTAAGTCCCGCAA<br>CGAGCGCAACCCTTATCGTTAGTTGCCAGCACTTAGGGTGGGAACTCTAACGA<br>GACTGCCTGGGTTAACCAGGAGGAAGGCGAGGATGACGTCAAGTCAGCATGG<br>CCCTTATGCCCAGGGCGACACACGTGCTACAATGGCCAGTACAGAAGGTGGC

	AAGATCGCGAGATGGAGCAAATCCTCAAAGCTGGCCCCAGTTCGGATTGTAGT CTGCAACTCGACTACATGAAGTCGGAATTGCTAGTAATGGCGTGTCAGCCATA ACGCCGTGAATACGTTCCCGGGCCTTGTACACACCGCCCGTCACATCATGGG AGTTGGTTTTACCTTAAGTCGTTGACTCAACCCGCAAGGAGAGAGGCGCCCAA GGTGAGGCTGATGACTAGGATGAAGTCGTAACAAGGTAGCCCTACCGGAAGG TGGGGCTGGATCACCTCCTTT

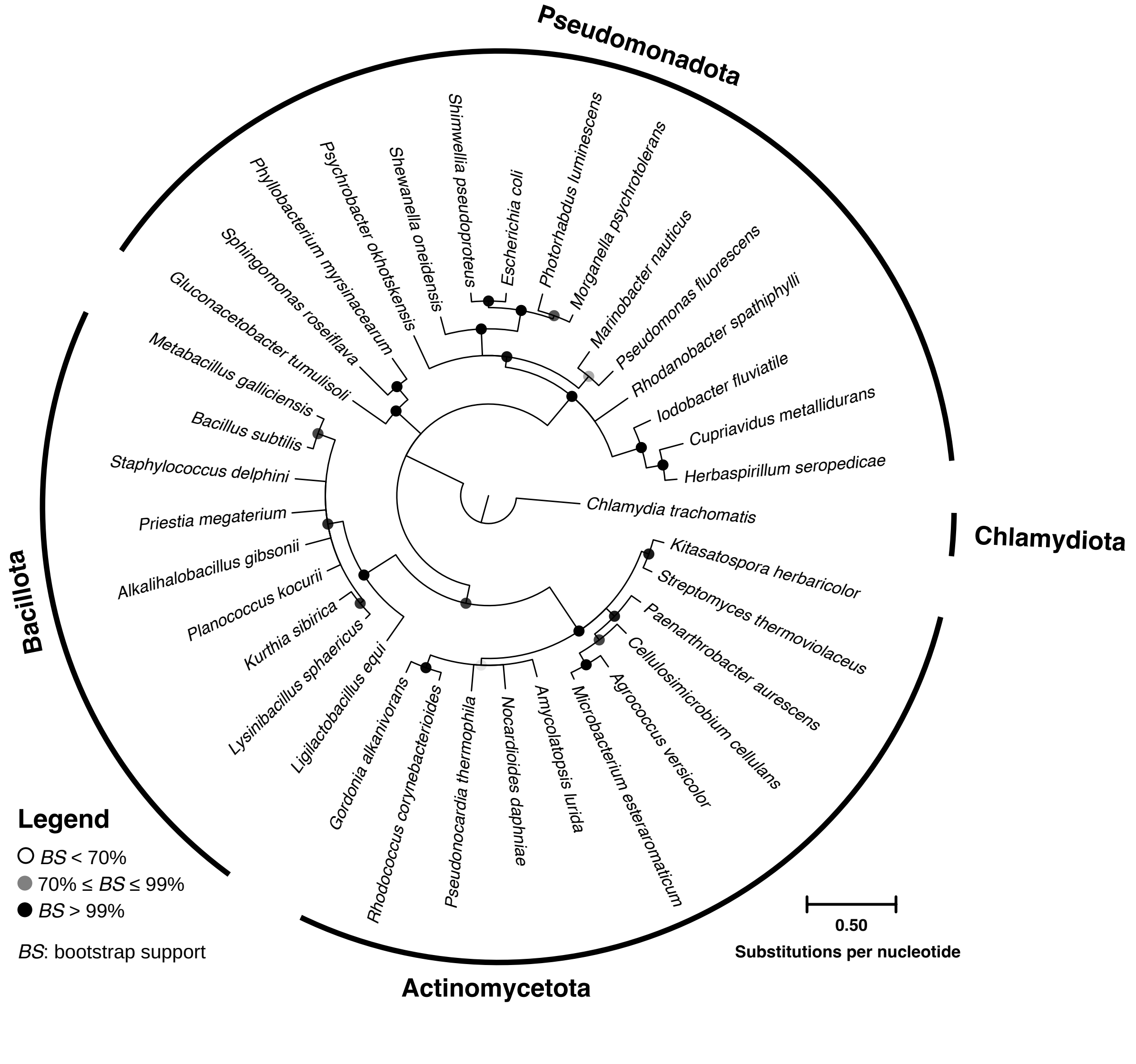

Figure S1: Maximum Likelihood 16s rRNA phylogenetic tree showing the relation between species of the curated NCIMB screening panel.

Maximum Likelihood tree constructed using the General Time Reversible (GTR) substitution model with gamma distributed rates (+G) and invariable sites (+I). Tree shows consensus of nodes with >65% bootstrap support based upon 500 replicates in which associated taxa are clustered together. Bar represents 0.1 substitutions per nucleotide position.

S3.2 ^{31}P NMR standards

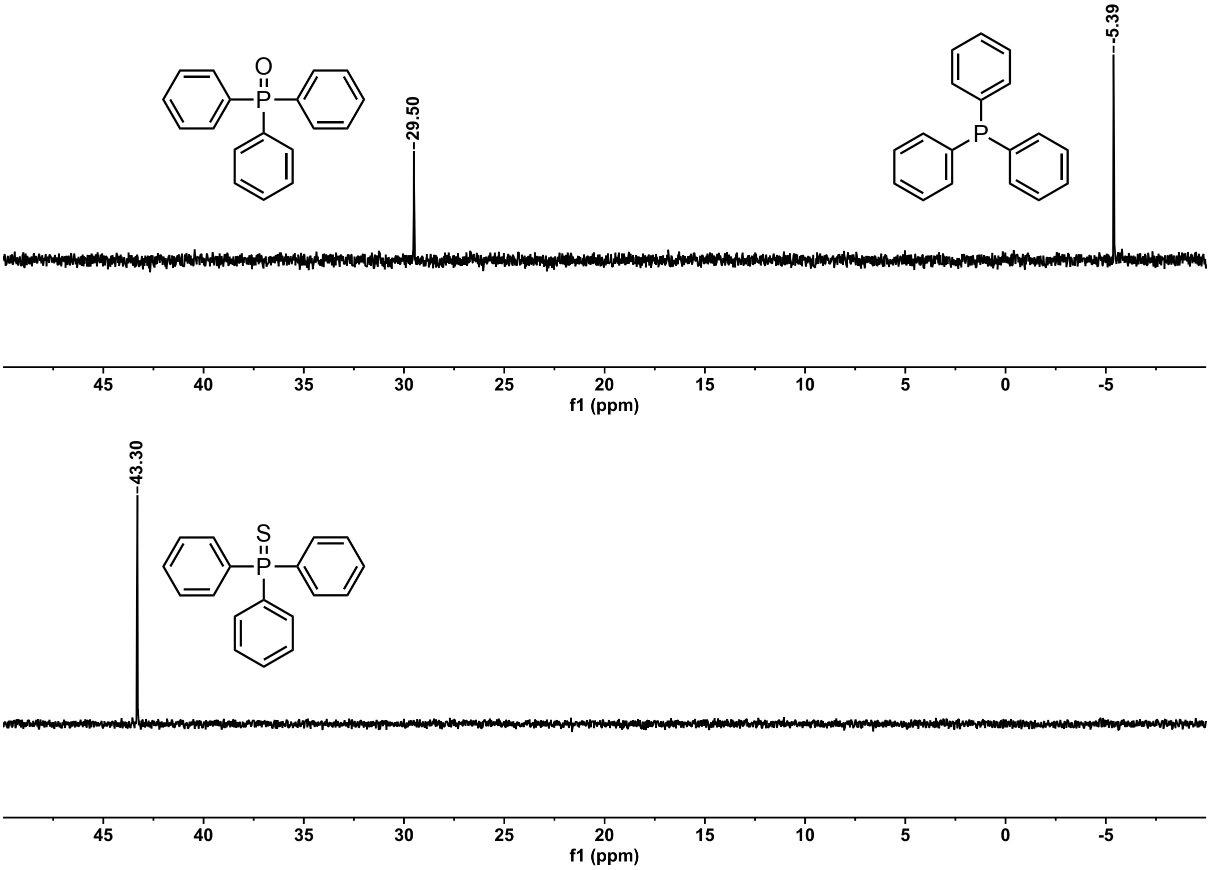

Figure S2: ^{31}P NMR analysis of phosphine standards

NMR peak shifts of commercial triphenylphosphine (δ -5.39 ppm), triphenylphosphine oxide (δ +29.50 ppm), and triphenylphosphine sulfide (δ +43.30 ppm) in $CDCl_3$. Samples analysed by H^+-decoupled ^{31}P NMR using a Bruker Pro500 spectrometer.

S3.3 Triphenylphosphine oxide reduction ^{31}P NMR traces

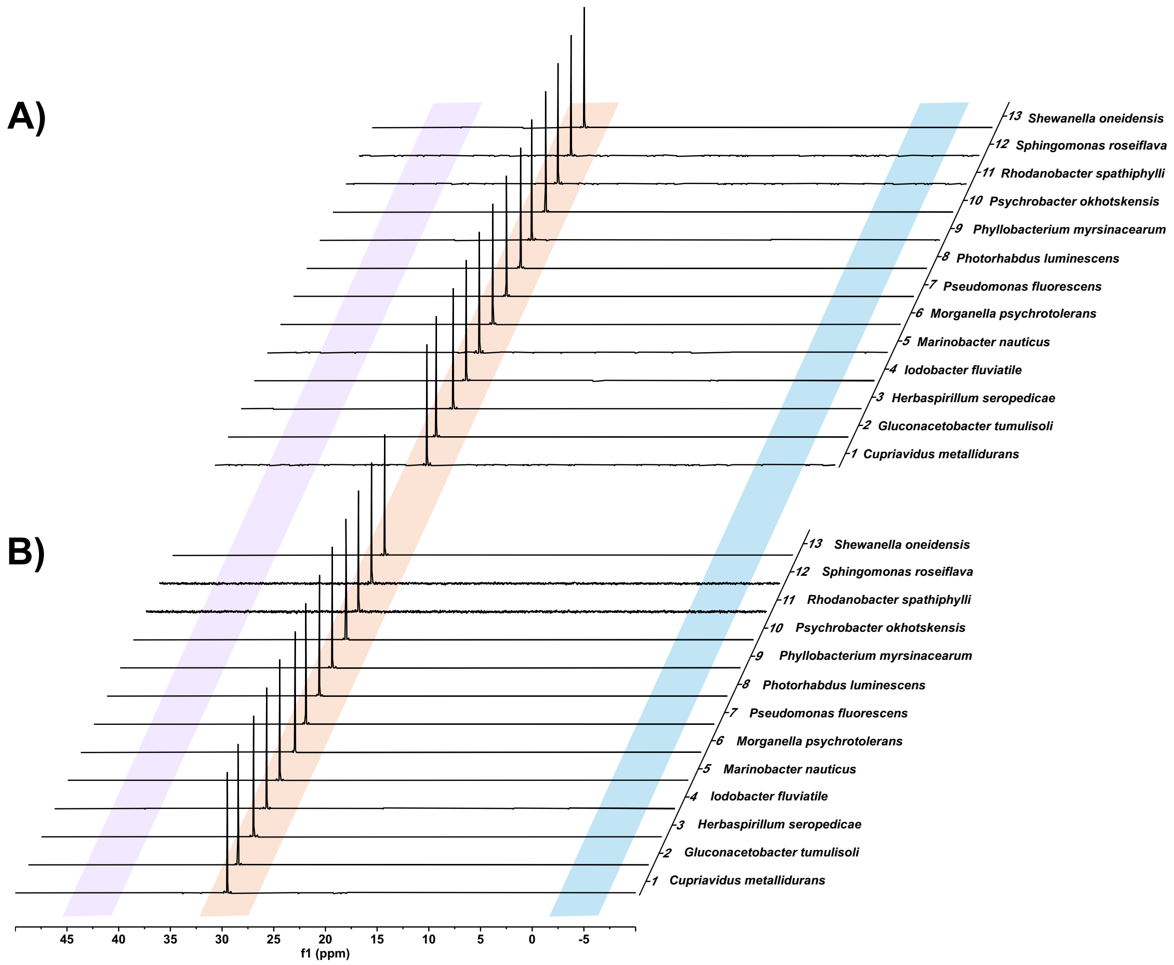

Figure S3: Stacked ^{31}P NMR spectra of A) aerobic and B) anaerobic Ph_3PO reduction screens for the *Pseudomonadota* phylum.

Reduction of 3 mM Ph_3PO to Ph_3P by *Pseudomonadota* from the curated NCIMB culture collection under A) aerobic and B) anaerobic conditions. Data presented qualitatively with local normalisation of peak size, including expected shift patterns for Ph_3P (blue; δ -5.39 ppm), Ph_3PO (orange; δ +29.50 ppm), and Ph_3PS (purple; δ +43.30 ppm). Completed in single replicate (n=1).

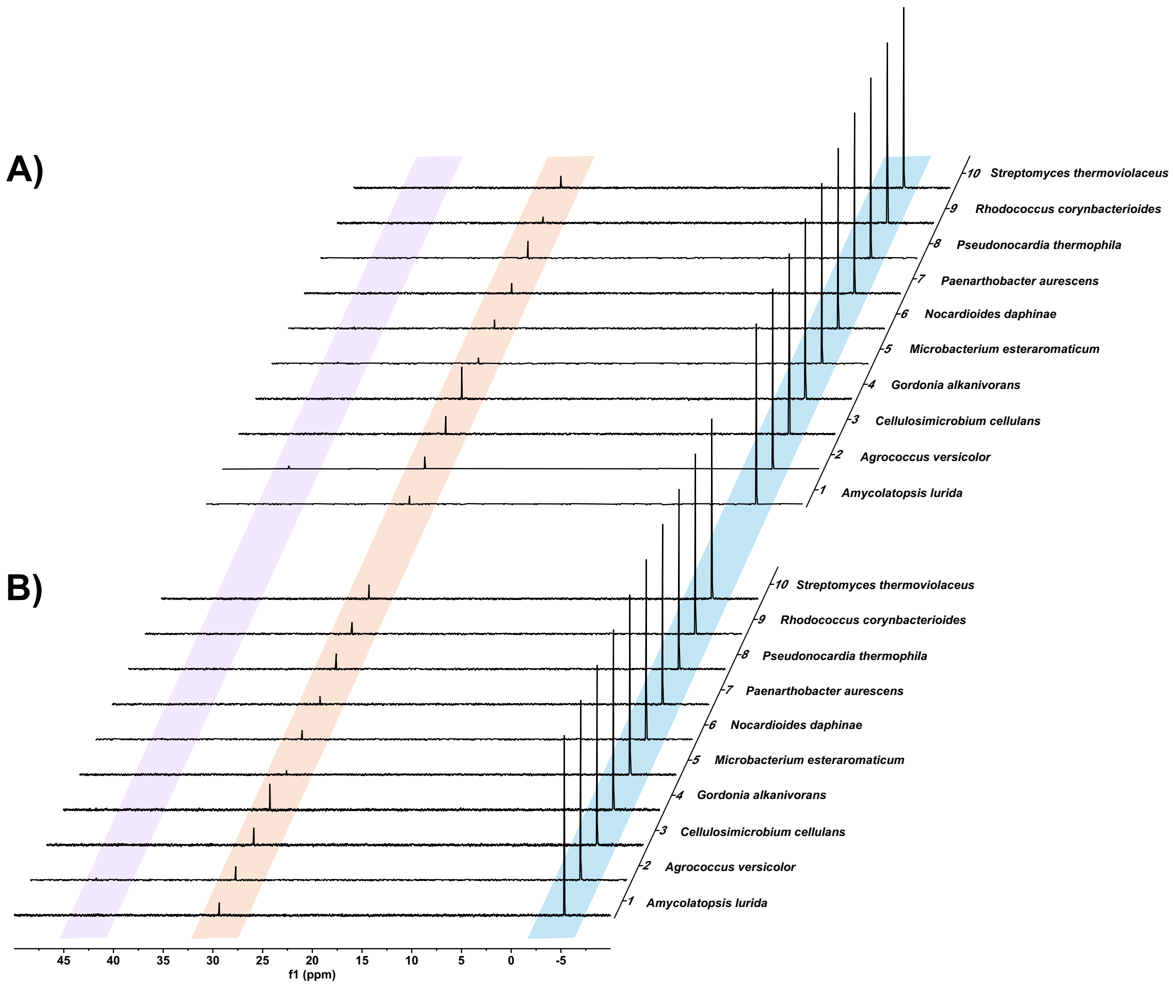

Figure S4: Stacked ^{31}P NMR spectra of A) aerobic and B) anaerobic Ph_3PO reduction screens for the *Actinomycetota* phylum.

Reduction of 3 mM Ph_3PO to Ph_3P by *Actinomycetota* from the curated NCIMB culture collection under A) aerobic and B) anaerobic conditions. Data presented qualitatively with local normalisation of peak size, including expected shift patterns for Ph_3P (blue; δ -5.39 ppm), Ph_3PO (orange; δ +29.50 ppm), and Ph_3PS (purple; δ +43.30 ppm). Completed in single replicate (n=1).

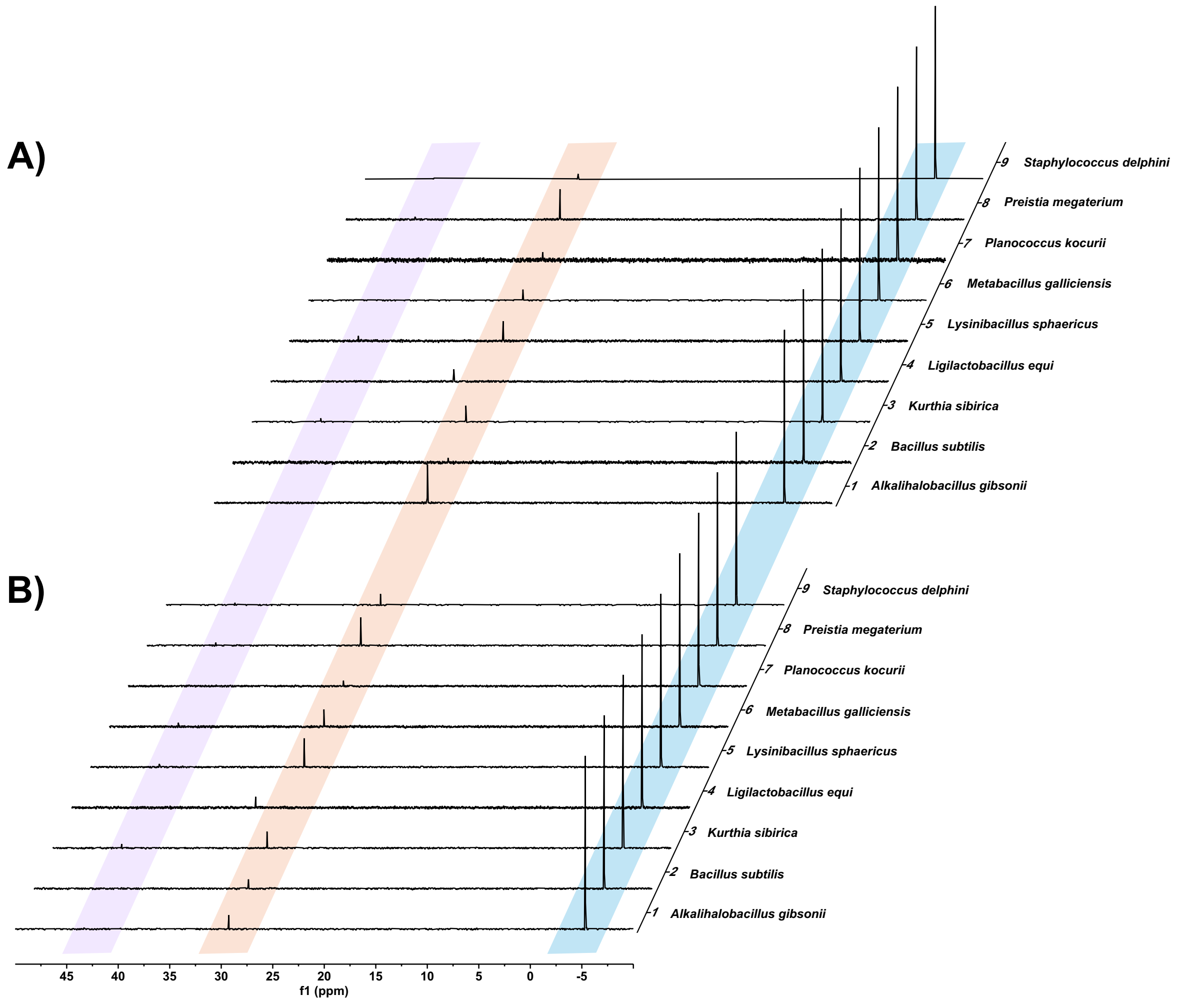

Figure S5: Stacked ^{31}P NMR spectra of A) aerobic and B) anaerobic Ph_3PO reduction screens for the *Bacillota* phylum.

Reduction of 3 mM Ph3PO to Ph3P by *Bacillota* from the curated NCIMB culture collection under A) aerobic and B) anaerobic conditions. Data presented qualitatively with local normalisation of peak size, including expected shift patterns for Ph_3P (blue; δ -5.39 ppm), Ph_3PO (orange; δ +29.50 ppm), and Ph_3PS (purple; δ +43.30 ppm). Shift observed in the *Ligolactobacillus equi* Ph_3PO peak attributed to lactic acid production during stationary phase, resulting in a change in resonance frequency. Completed in single replicate (n=1).

S3.4 Triphenylphosphine modification ^{31}P NMR traces

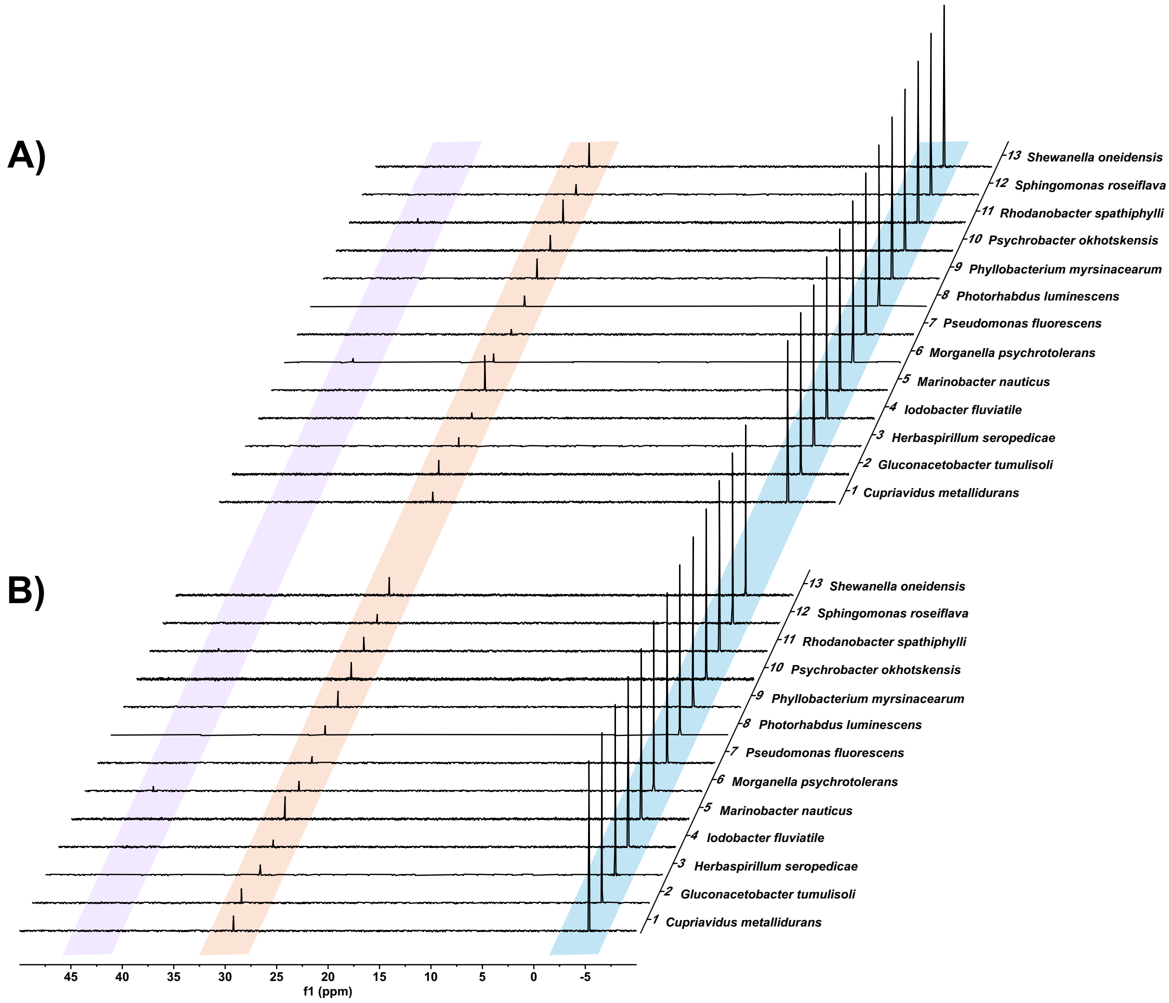

Figure S6: Stacked ^{31}P NMR spectra of A) aerobic and B) anaerobic Ph_3P modification screens for the *Pseudomonadota* phylum.

Modification of 3 mM Ph_3P by *Pseudomonadota* from the curated NCIMB culture collection under A) aerobic and B) anaerobic conditions. Data presented qualitatively with local normalisation of peak size, including expected shift patterns for Ph_3P (blue; δ -5.39 ppm), Ph_3PO (orange; δ +29.50 ppm), and Ph_3PS (purple; δ +43.30 ppm). Completed in single replicate (n=1).

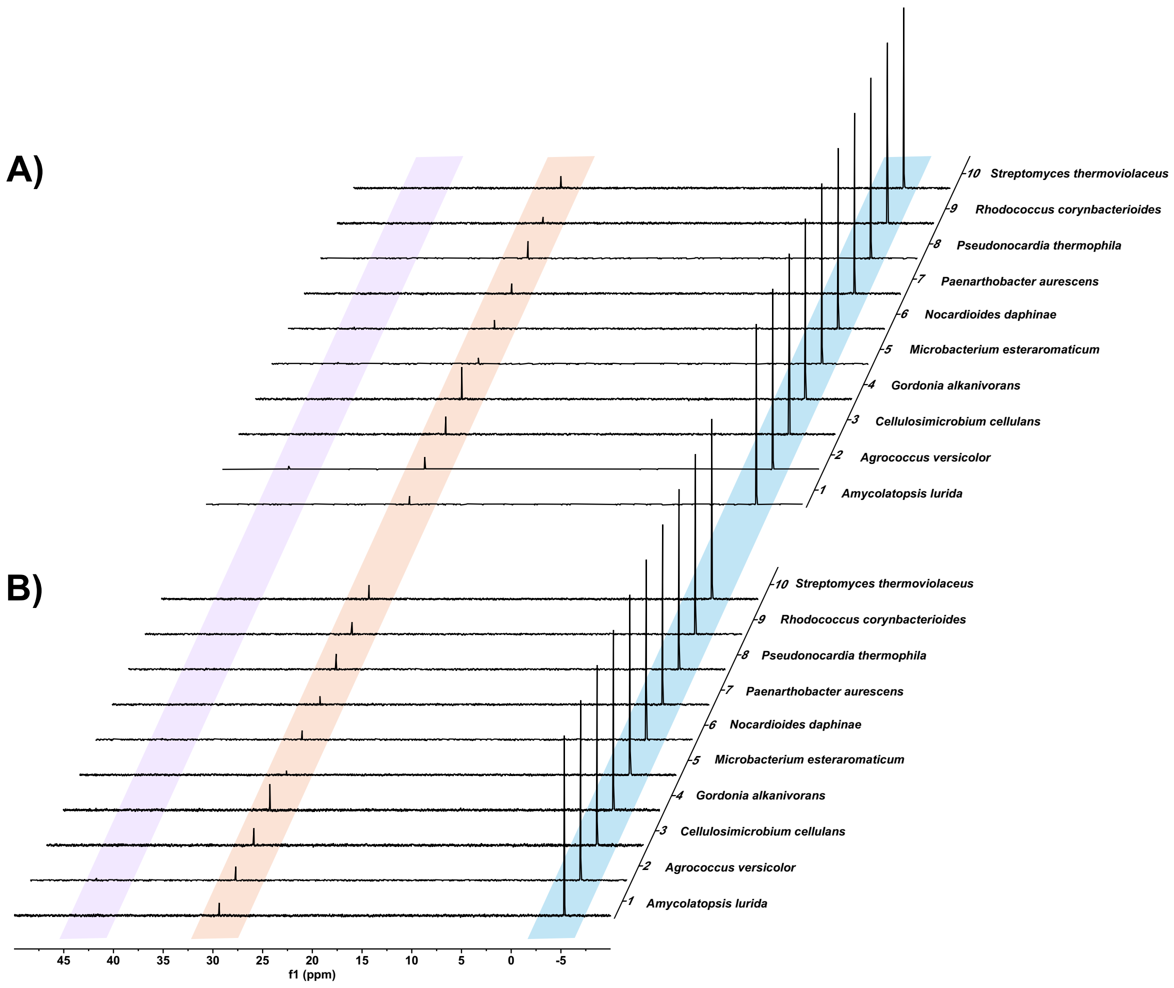

Figure S7: Stacked ^{31}P NMR spectra of A) aerobic and B) anaerobic Ph_3P modification screens for the *Actinomycetota* phylum.

Modification of 3 mM Ph_3P by *Actinomycetota* from the curated NCIMB culture collection under A) aerobic and B) anaerobic conditions. Data presented qualitatively with local normalisation of peak size, including expected shift patterns for Ph_3P (blue; δ -5.39 ppm), Ph_3PO (orange; δ +29.50 ppm), and Ph_3PS (purple; δ +43.30 ppm). Completed in single replicate (n=1).

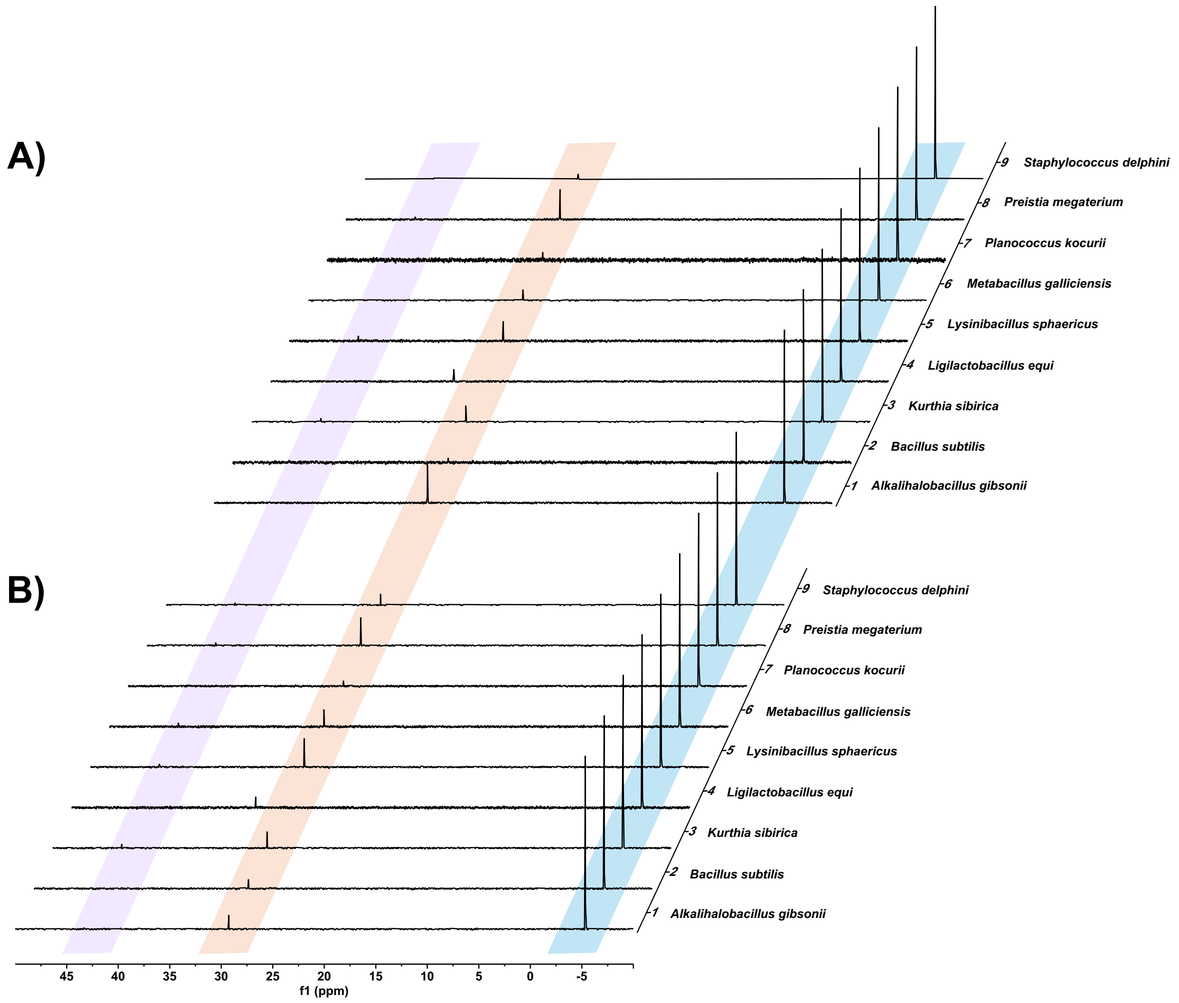

Figure S8: Stacked ^{31}P NMR spectra of A) aerobic and B) anaerobic Ph_3P modification screens for the *Bacillota* phylum.

Modification of 3 mM Ph_3P by *Bacillota* from the curated NCIMB culture collection under A) aerobic and B) anaerobic conditions. Data presented qualitatively with local normalisation of peak size, including expected shift patterns for Ph_3P (blue; δ -5.39 ppm), Ph_3PO (orange; δ +29.50 ppm), and Ph_3PS (purple; δ +43.30 ppm). Completed in single replicate (*n*=1).

A)

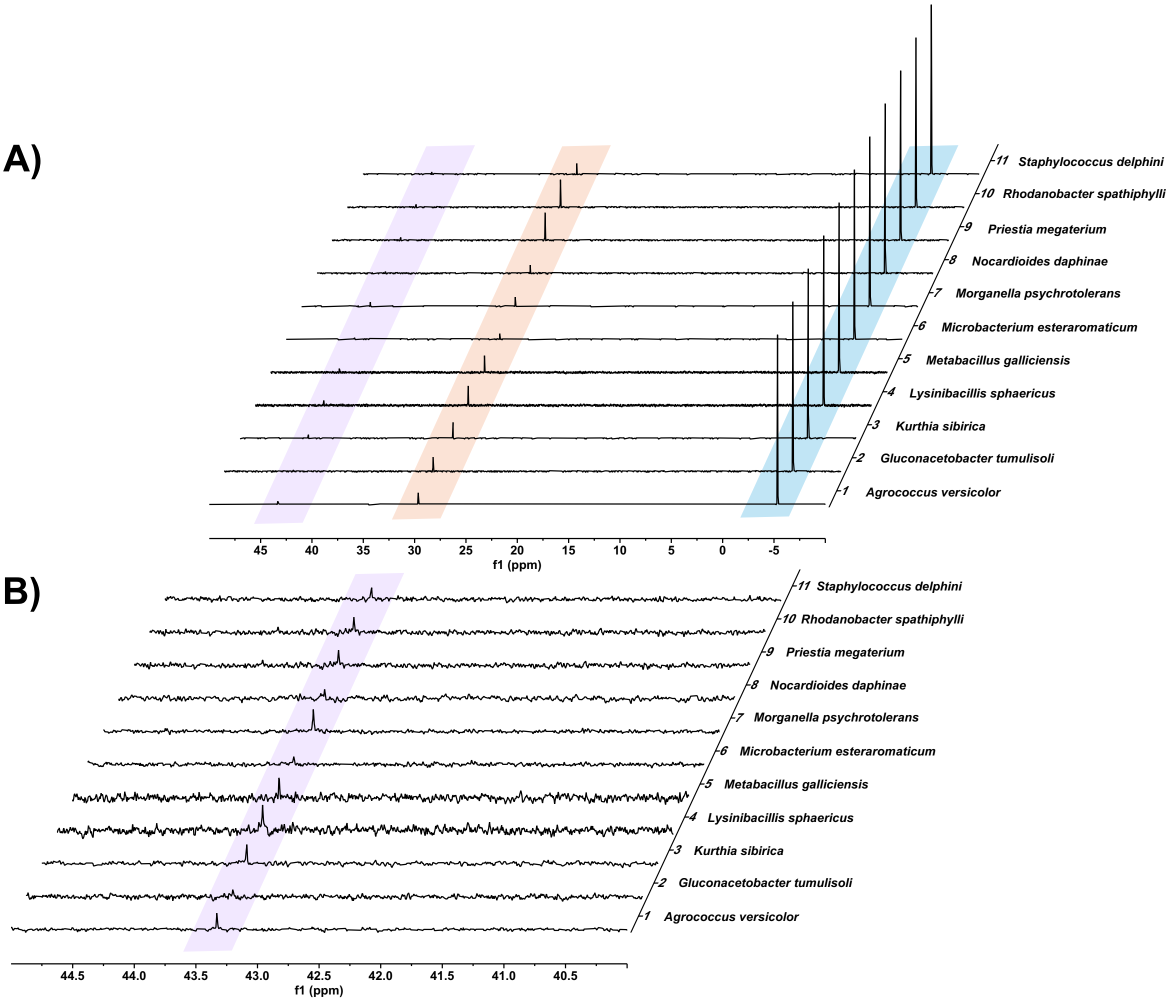

B)

Figure S9: Stacked ^{31}P NMR spectra of all species which facilitate Ph_3P modification.

Modification of 3 mM Ph_3P by curated bacteria from the NCIMB culture collection, showing A) the whole Ph_3P range and B) the corresponding Ph3PS peak. Data presented qualitatively with local normalisation of peak size, including expected shift patterns for Ph_3P (blue; δ -5.39 ppm), Ph_3PO (orange; δ +29.50 ppm), and Ph_3PS (purple; δ +43.30 ppm). Completed in single replicate (*n*=1).

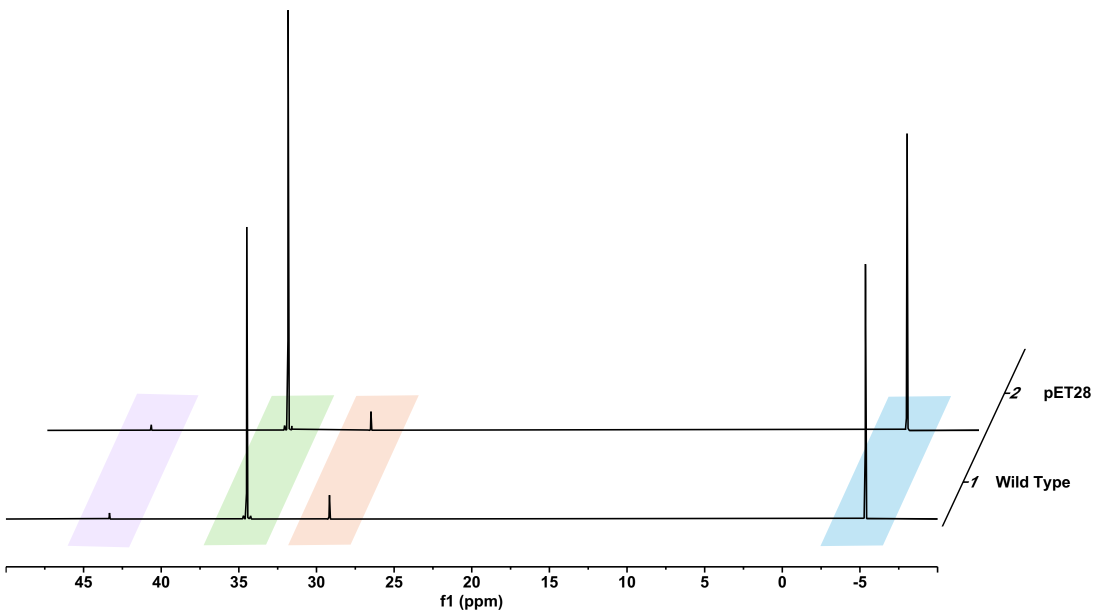

Figure S10: ^{31}P NMR spectra of Ph_3P modification by *Escherichia coli* BW25113.

Modification of 3 mM Ph_3P by *Escherichia coli* under aerobic conditions. Data presented qualitatively with local normalisation of peak size, including expected shift patterns for Ph_3P (blue; δ -5.39 ppm), Ph_3PO (orange; δ +29.50 ppm), $CyPh_2PO$ (green; δ + 34.50 ppm), and Ph_3PS (purple; δ +43.30 ppm). Completed in single replicate (*n*=1).

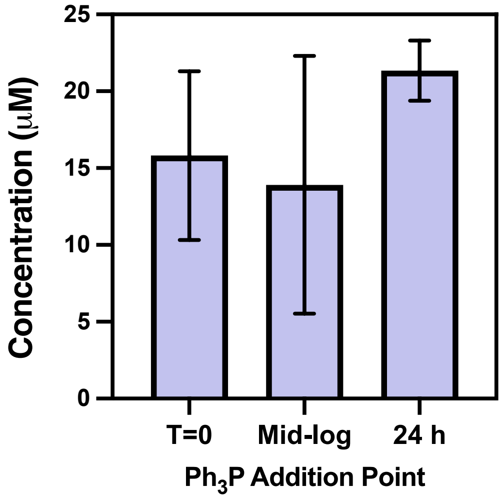

Figure S11: Ph_3PS by *Escherichia coli* BW25113 at different culture growth points.

Ph_3PS formation by *Escherichia coli* under aerobic conditions when 3 mM Ph_3P is added at inoculation (OD_{600} 0.01), mid-logarithmic stage (OD_{600} 0.47) and stationary phase (24 h incubation, OD_{600} 3.86). Concentrations quantified relative to a known concentration of $CyPh_2PO$ as an internal standard for quantification. Error bars represent standard error of the mean (*n*=3).

S3.5 Effect of sulfur metabolism on P=S formation in *E. coli*

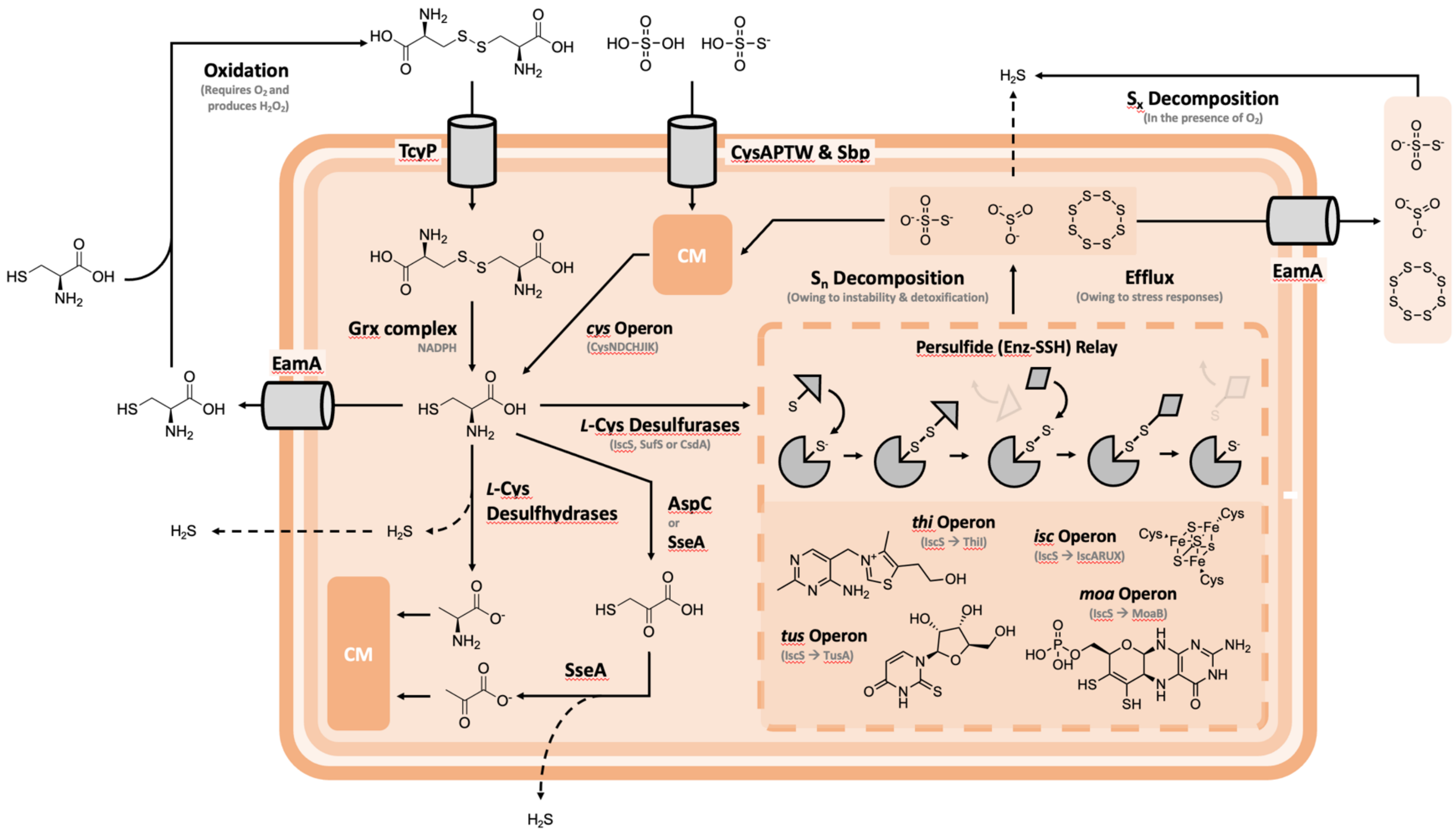

Figure S12: Overview of relevant cysteine metabolism in *E. coli* K12 MG1655.

Visual schematic overview of cysteine import, efflux, degradation, and assimilation (central metabolism; CM) by *E. coli* K12 MG1655. Model constructed using relevant Kyoto Encyclopedia of Genes and Genomes entries (KEGG; accessible at: https://www.kegg.jp) including sulfur metabolism (eco00920), cysteine and methionine metabolism (eco00270), and sulfur relay system (eco04122).

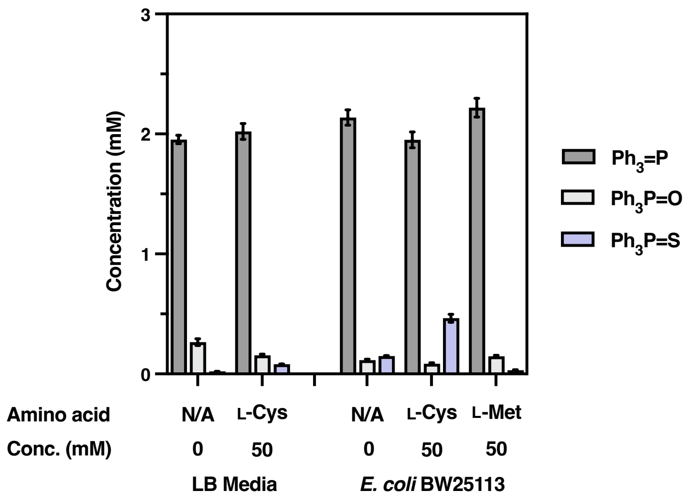

Figure S13: Mean production of Ph_3PS in both LB media and *E. coli* BW25113 supplemented with 50 mM L-cysteine or L-methionine.

Effect of 50 mM L-Cys or L-Met on P=S formation in LB media and *E. coli* BW25113 cultures. Concentrations quantified relative to a known concentration of $CyPh_2PO$ as an internal standard for quantification. Error bars represent standard error of the mean (*n*=3).

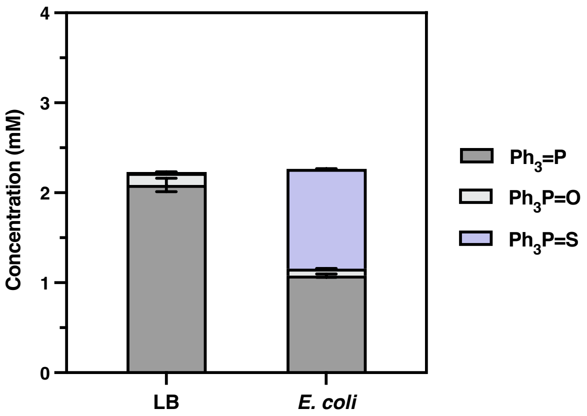

Figure S14: Mean production of Ph_3PS in both LB media and *E. coli* BW25113 supplemented with 25 mM L-cystine.

Effect of 25 mM L-cystine on P=S bond formation in LB media and *E. coli* BW25113 demonstrating a further dramatic increase in Ph_3PS formation. Concentrations quantified relative to a known concentration of $CyPh_2PO$ as an internal standard for quantification. Error bars represent standard error of the mean (*n*=3).

S3.6 Identifying the interactions between biotic and abiotic P=S formation

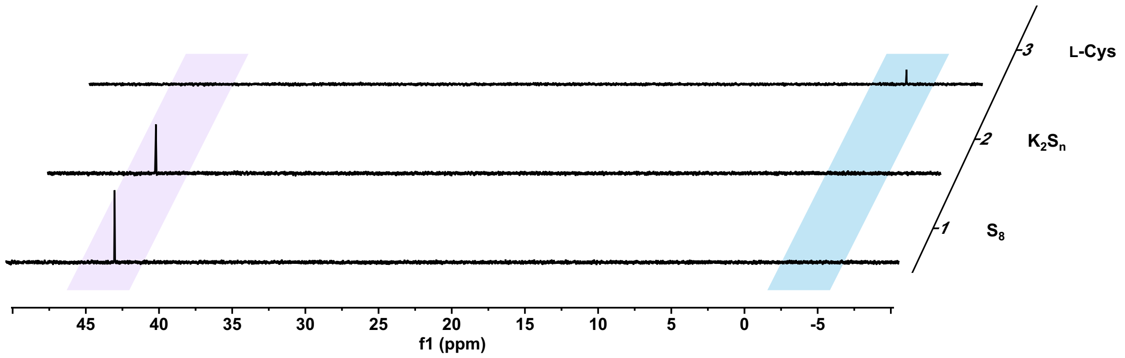

Figure S155: ^{31}P NMR spectra of Ph_3P modification by various sulfane sulfur sources *in vitro*.

Ph_3PS formation by reaction of Ph_3P with biologically-relevant sulfane sulfur-containing compounds. All reactions completed *in vitro* by mixing dissolved Ph_3P with dissolved sulfur donor. ^{31}P NMR spectroscopy data presented qualitatively with local normalisation of peak size, including expected shift patterns for Ph_3P (blue; δ -5.39 ppm) and Ph_3PS (purple; δ +43.30 ppm). Completed in triplicate, representative spectrum shown (*n*=1).

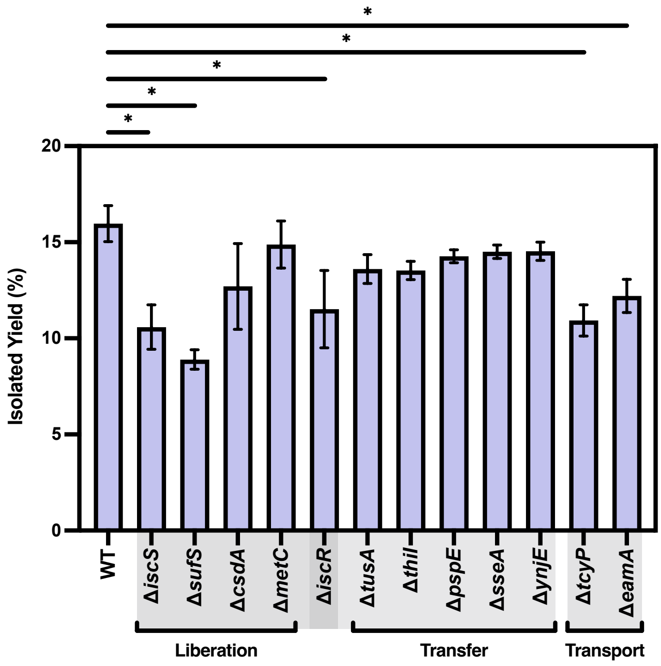

Figure S16: Production of Ph_3PS in *E. coli* BW25113 single knockouts supplemented with 50 mM L-cysteine.

Conversion of Ph_3P to Ph_3PS by *E. coli* BW25113 single gene knockouts belonging to sulfur liberation, transfer, and transport groups when supplemented with 50 mM L-Cys. Δ*iscR* belongs to both sulfur liberation and sulfur transfer groups. Concentrations quantified relative to a known concentration of $CyPh_2PO$ as an internal standard for quantification. Groups

investigated individually as technical triplicates (*n*=3), each with their own WT control (*n*=9). Error bars represent standard error of the mean. * represents groups that are significantly different from the WT control (Two-way ANOVA, F= 6.55, *df* = 24, P <0.05).

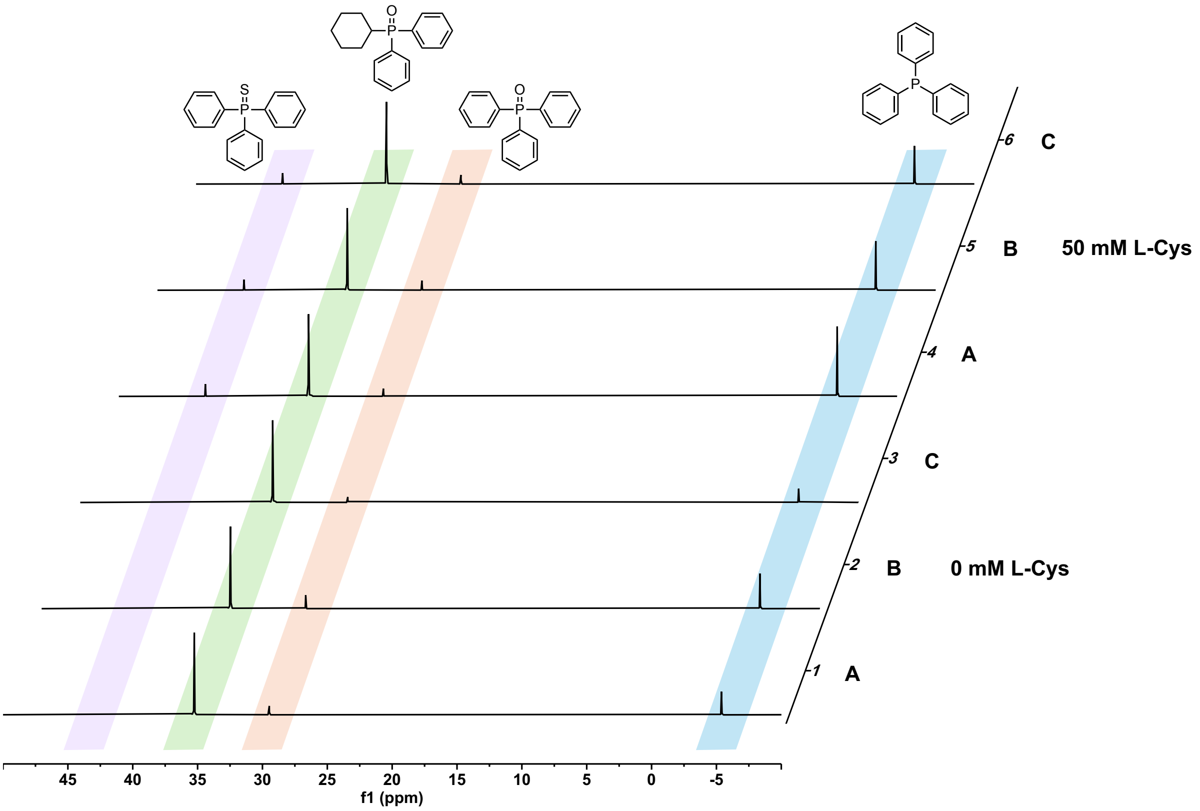

Figure S17: ^{31}P NMR spectra of Ph_3P modification using evolved headspace gas.

Ph_3PS formation in LB media containing with 5 mL headspace gas derived from *E. coli* BW25113 cultures supplemented with 50 mM L-Cys. Gas collected after 48 h growth at 37 °C, 220 rpm. Data presented qualitatively with local normalisation of peak size including expected shift patterns for Ph_3P (blue; δ -5.39 ppm), Ph_3PO (orange; δ +29.50 ppm), $CyPh_2PO$ (green; δ + 34.50 ppm), and Ph_3PS (purple; δ +43.30 ppm).Completed in technical triplicate (*n*=3).

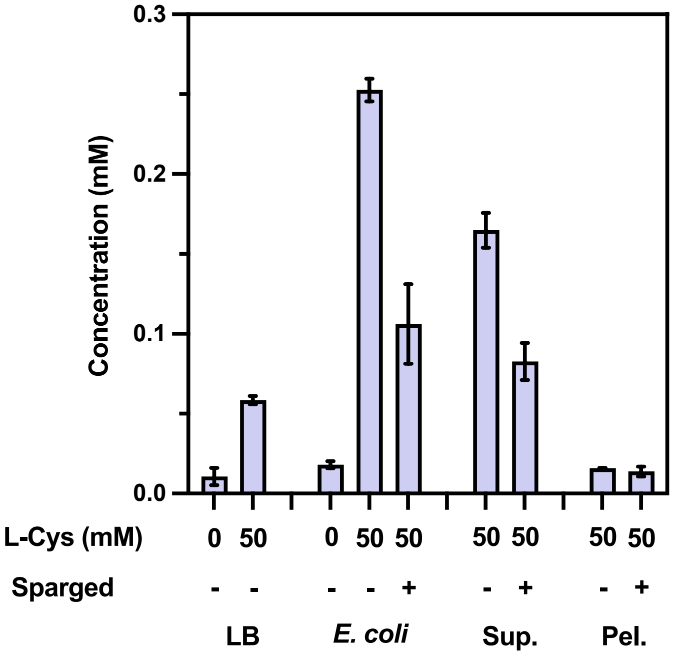

Figure S18: Mean production of Ph_3PS in separated *Escherichia coli* cultures.

Ph_3PS formation by 10 mL LB or separated *E. coli* BW25113 cultures (supernatant (Sup.) and cell pellet (Pel.)) ± 50 mM L-Cys. All samples incubated at 37 °C, 220 rpm for 48 h. Concentrations quantified relative to a known concentration of $CyPh_2PO$ as an internal standard for quantification. Error bars represent standard error of the mean (*n*=3).

S4 Phosphine Substrate Scope

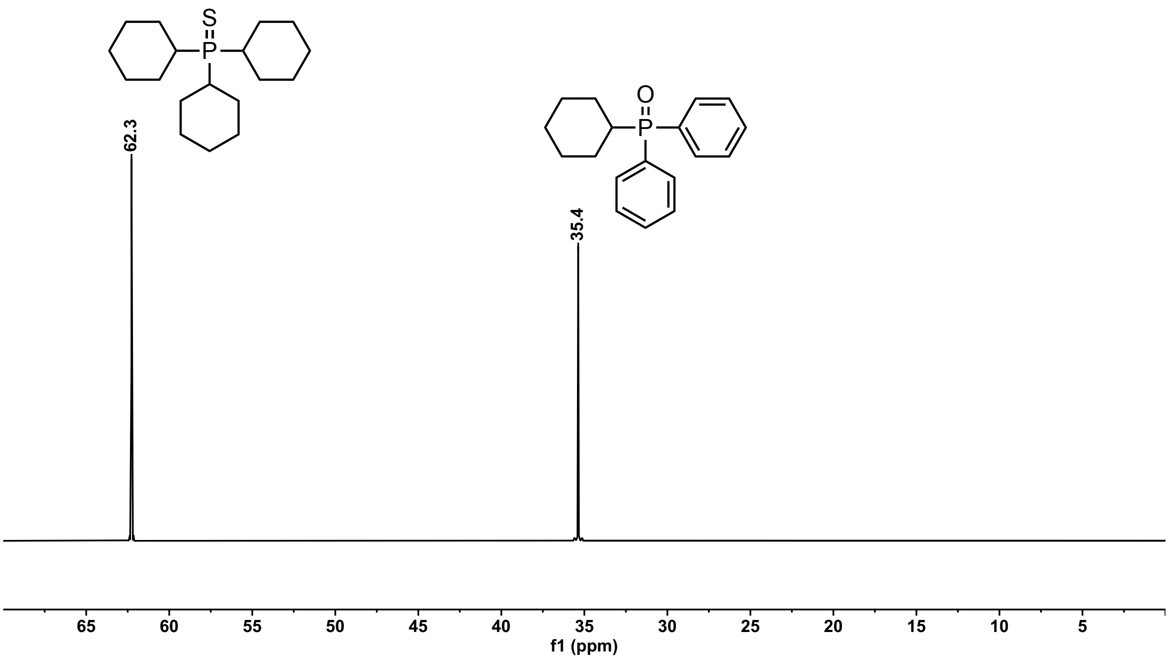

Figure S19: ^{31}P NMR spectrum of tricyclohexylphosphine modification by *Escherichia coli* BW25113.

Modification of 3 mM tricyclohexylphosphine by *Escherichia coli* under aerobic conditions when supplemented with 50 mM L-Cys. ^{31}P NMR (500 MHz, $CDCl_3$) δ/ppm: 62.0 ($SPCy_3$). Spectroscopic data in good agreement with literature[7]. Completed in triplicate, representative spectrum shown (*n*=1).

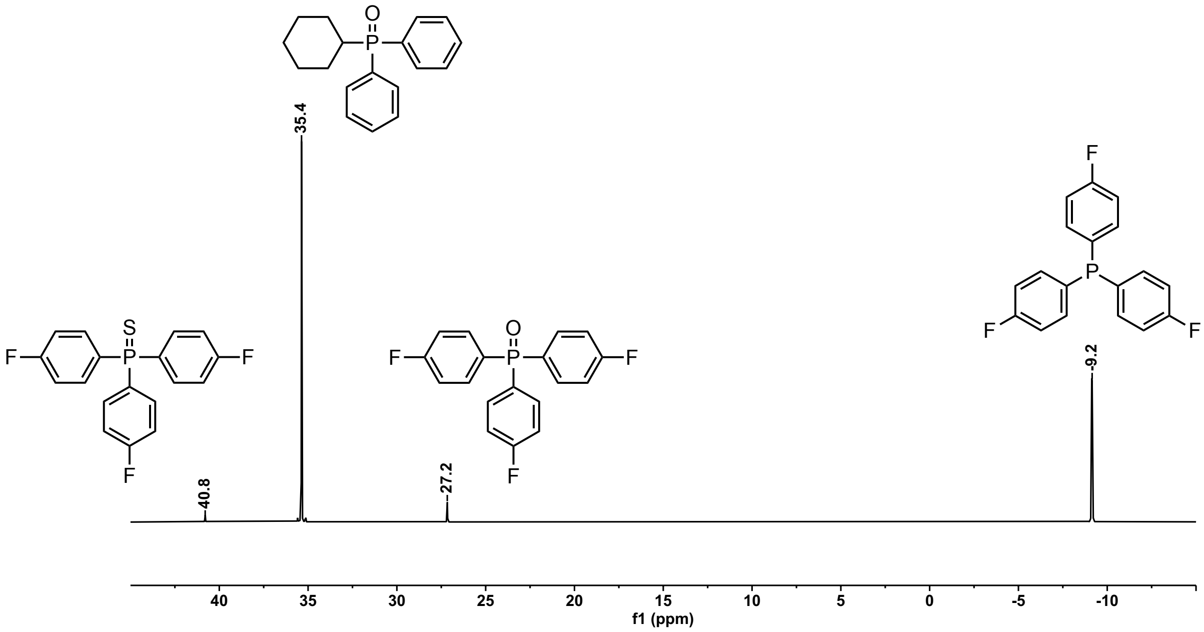

Figure S20: ^{31}P NMR spectrum of tris(4-fluorephenyl)phosphine modification by *Escherichia coli* BW25113.

Modification of 3 mM tris(4-fluorephenyl)phosphine by *Escherichia coli* under aerobic conditions when supplemented with 50 mM L-Cys. ^{31}P NMR (500 MHz, $CDCl_3$) δ/ppm: 40.8 ($SP(4FPh)_3$), 27.2 ($OP(4FPh)_3$), -9.2 ($P(4FPh)_3$). Spectroscopic data in good agreement with literature[8,9]. Completed in triplicate, representative spectrum shown (*n*=1).

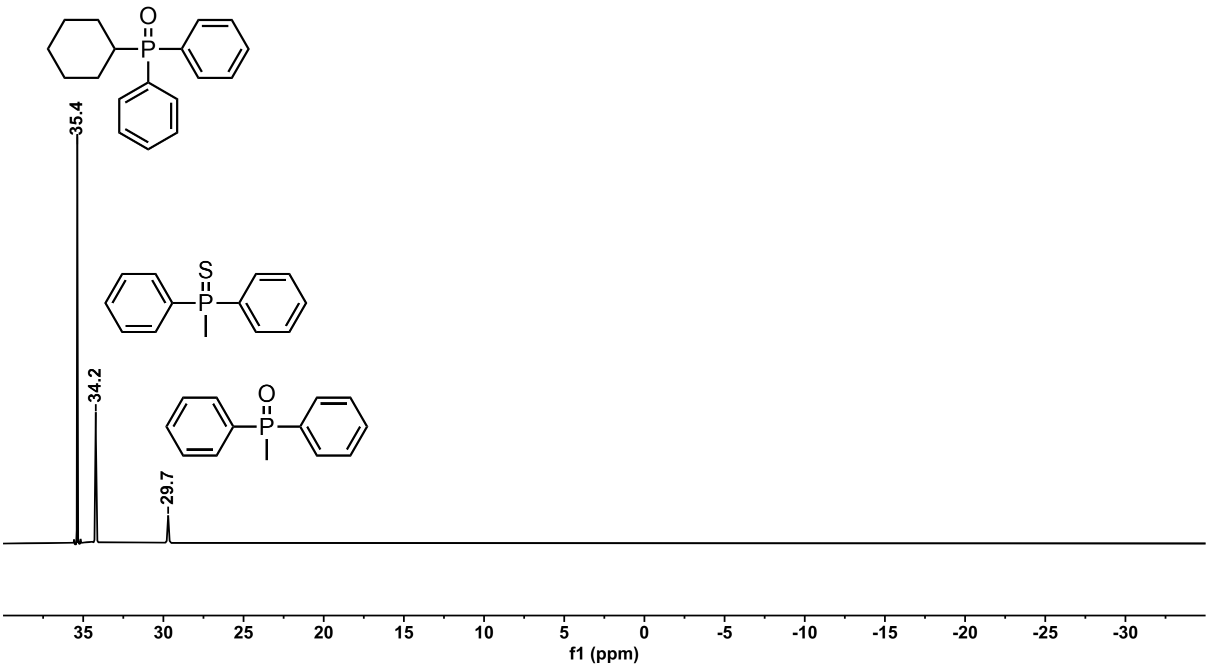

Figure S21: ^{31}P NMR spectrum of methyldiphenylphosphine modification by *Escherichia coli* BW25113.

Modification of 3 mM methyldiphenylphosphine by *Escherichia coli* under aerobic conditions when supplemented with 50 mM L-Cys. ^{31}P NMR (500 MHz, $CDCl_3$) δ/ppm: 35.6 ($SPMePh_2$), 29.9 ($OPMePh_2$). Spectroscopic data in good agreement with literature[10,11]. Completed in triplicate, representative spectrum shown (*n*=1).

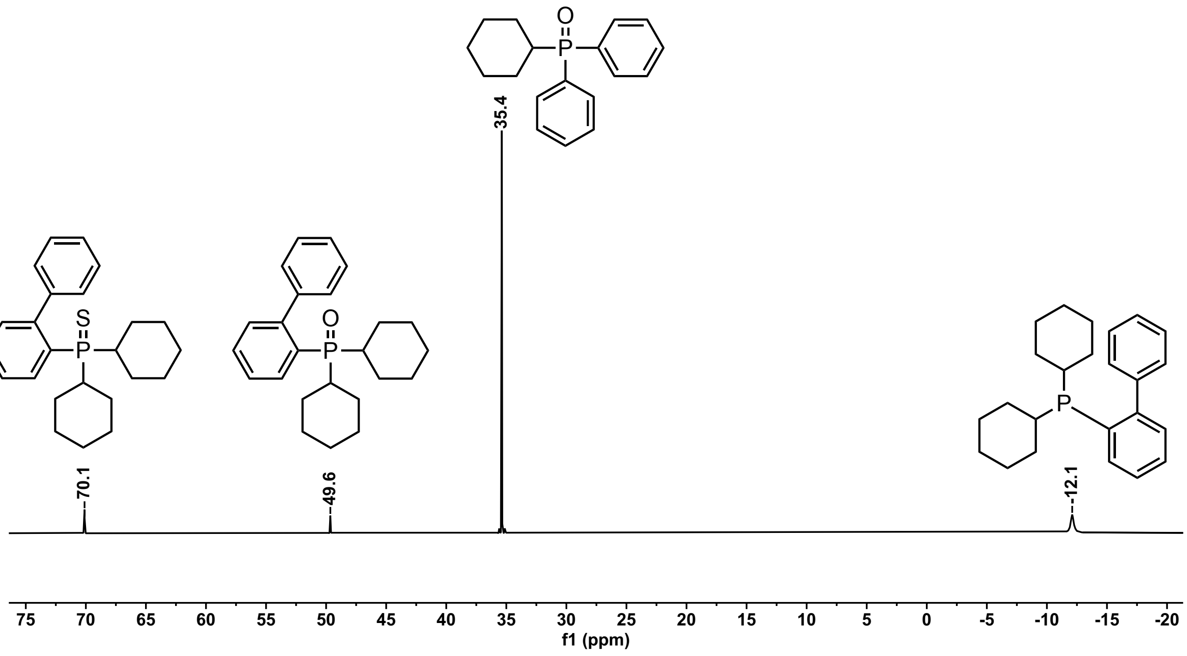

Figure S22: ^{31}P NMR spectrum of [1,1'-Biphenyl]-2-yldicyclohexylphosphine (CyJohnPhos) modification by *Escherichia coli* BW25113.

Modification of 3 mM CyJohnPhos by *Escherichia coli* under aerobic conditions when supplemented with 50 mM L-Cys. ^{31}P NMR (500 MHz, $CDCl_3$) δ/ppm: 70.1 (SP(1,1'-biphenyl)Cy_2), 49.6 (OP(1,1'-biphenyl)Cy_2), -12.1 ((1,1'-biphenyl)Cy_2). Spectroscopic data in good agreement with literature[12,13] . Completed in triplicate, representative spectrum shown (n=1).

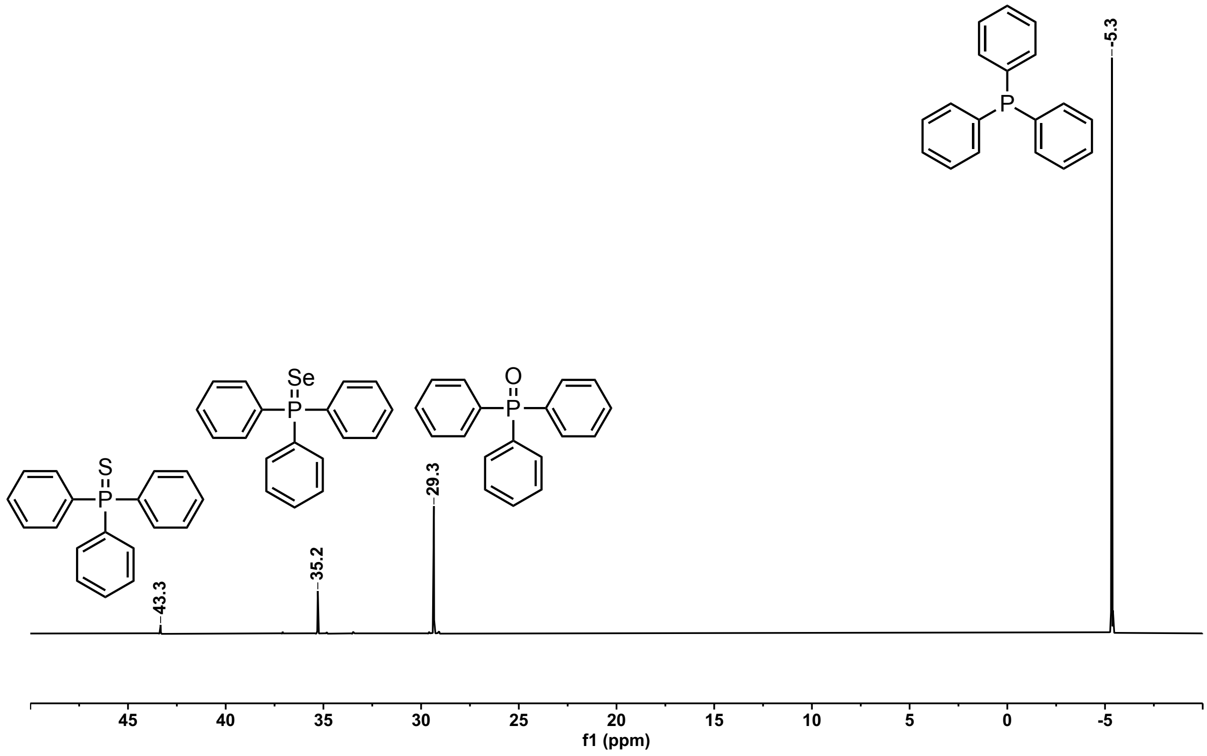

Figure S23: Production of Ph_3PSe by *Escherichia coli* BW25113 pET28 supplemented with 25 mM L-selenocystine.

Modification of 3 mM Ph_3P to form Ph_3PSe by *E. coli* BW25113 under aerobic conditions. Data presented qualitatively, including expected shift patterns for Ph_3P (δ -5.35 ppm), Ph_3PO (δ +29.34 ppm), Ph_3PSe (δ + 35.29 ppm), and Ph_3PS (δ +43.33 ppm). Completed in single replicate (*n*=1).

S5 References

(1) Lemoine, F.; Correia, D.; Lefort, V.; Doppelt-Azeroual, O.; Mareuil, F.; Cohen-Boulakia, S.; Gascuel, O. NGPhylogeny.Fr: New Generation Phylogenetic Services for Non-Specialists. *Nucleic Acids Research* **2019**, *47* (W1), W260–W265. https://doi.org/10.1093/NAR/GKZ303.

(2) Tamura, K.; Stecher, G.; Kumar, S. MEGA11: Molecular Evolutionary Genetics Analysis Version 11. *Molecular Biology and Evolution* **2021**, *38* (7), 3022–3027. https://doi.org/10.1093/molbev/msab120.

(3) Parte, A. C.; Sardà Carbasse, J.; Meier-Kolthoff, J. P.; Reimer, L. C.; Göker, M. List of Prokaryotic Names with Standing in Nomenclature (LPSN) Moves to the DSMZ. *International Journal of Systematic and Evolutionary Microbiology*, 2020, *70*, 5607–5612. https://doi.org/10.1099/ijsem.0.004332.

(4) Katoh, K.; Standley, D. M. MAFFT Multiple Sequence Alignment Software Version 7: Improvements in Performance and Usability. *Molecular biology and evolution* **2013**, *30* (4), 772–780. https://doi.org/10.1093/molbev/mst010.

(5) Criscuolo, A.; Gribaldo, S. BMGE (Block Mapping and Gathering with Entropy): A New Software for Selection of Phylogenetic Informative Regions from Multiple Sequence Alignments. *BMC evolutionary biology* **2010**, *10*, 210. https://doi.org/10.1186/1471-2148-10-210.

(6) Bianchini, G.; Sánchez-Baracaldo, P. TreeViewer: Flexible, Modular Software to Visualise and Manipulate Phylogenetic Trees. *Ecology and Evolution* **2024**, *14* (2), e10873. https://doi.org/10.1002/ece3.10873.

(7) Xiong, Y.; Wu, X. Deoxygenative Coupling of Alcohols with Aromatic Nitriles Enabled by Direct Visible Light Excitation. *Org. Biomol. Chem.* **2023**, *21* (47), 9316–9320. https://doi.org/10.1039/D3OB01676E.

(8) Alvarado, S. R.; Shortt, I. A.; Fan, H.-J.; Vela, J. Assessing Phosphine–Chalcogen Bond Energetics from Calculations. *Organometallics* **2015**, *34* (16), 4023–4031. https://doi.org/10.1021/acs.organomet.5b00428.

(9) Busacca, C. A.; Raju, R.; Grinberg, N.; Haddad, N.; James-Jones, P.; Lee, H.; Lorenz, J. C.; Saha, A.; Senanayake, C. H. Reduction of Tertiary Phosphine Oxides with DIBAL-H. *J. Org. Chem.* **2008**, *73* (4), 1524–1531. https://doi.org/10.1021/jo7024064.

(10) Woźnicki, P.; Korzeniowska, E.; Stankevič, M. Intramolecular Nucleophilic Substitution of ω-Haloalkylphosphine Derivatives. *J. Org. Chem.* **2017**, *82* (19), 10271–10296. https://doi.org/10.1021/acs.joc.7b01767.

(11) Denton, R. M.; An, J.; Adeniran, B.; Blake, A. J.; Lewis, W.; Poulton, A. M. Catalytic Phosphorus(V)-Mediated Nucleophilic Substitution Reactions: Development of a Catalytic Appel Reaction. *J. Org. Chem.* **2011**, *76* (16), 6749–6767. https://doi.org/10.1021/jo201085r.

(12) Kumar, R.; Kumar, S.; Pandey, M. K.; Kashid, V. S.; Radhakrishna, L.; Balakrishna, M. S. Synthesis of Phosphine Chalcogenides Under Solvent-Free Conditions Using a Rotary Ball Mill. *European Journal of Inorganic Chemistry* **2018**, *2018* (8), 1028–1037. https://doi.org/10.1002/ejic.201701414.

(13) Chen, X.; Wu, H.; Yu, R.; Zhu, H.; Wang, Z. Palladium-Catalyzed C–P(III) Bond Formation by Coupling ArBr/ArOTf with Acylphosphines. *J. Org. Chem.* **2021**, *86* (13), 8987–8996. https://doi.org/10.1021/acs.joc.1c00937.
